# Efficient preparation of unsymmetrical disulfides by nickel-catalyzed reductive coupling strategy

**DOI:** 10.1038/s41467-022-30256-0

**Published:** 2022-05-11

**Authors:** Fei Wang, Ying Chen, Weidong Rao, Lutz Ackermann, Shun-Yi Wang

**Affiliations:** 1 Key Laboratory of Organic Synthesis of Jiangsu Province, College of Chemistry, Chemical Engineering and Materials Science, Collaborative Innovation Center of Suzhou Nano Science and Technology Soochow University, Suzhou, 215123 China; 2 Key Laboratory of Biomass-based Green Fuels and Chemicals, College of Chemical Engineering, Nanjing Forestry University, Nanjing, 210037 China; 3 Institut für Organische und Biomolekulare Chemie Georg-August-Universität Göttingen Tammannstraße 2, 37077 Göttingen, Germany; 4 Wöhler Research Institute for Sustainable Chemistry Georg-August-Universität Göttingen Tammannstraße 2, 37077 Göttingen, Germany

**Keywords:** Homogeneous catalysis, Synthetic chemistry methodology

## Abstract

Disulfides are widely found in natural products and find a wide range of applications in life sciences, materials chemistry and other fields. The preparation of disulfides mainly rely on oxidative couplings of two sulfur containing compounds. This strategy has many side reactions and other shortcomings. Herein, we describe the reductive nickel-catalyzed cross-electrophile coupling of unactivated alkyl bromides with symmetrical alkyl- and aryltetrasulfides to form alkyl-alkyl and aryl-alkyl unsymmetrical disulfides. This approach for disulfide synthesis is practical, relies on easily available, unfunctionalized substrates, and is scalable. We investigated the mechanism of this transformation and found that the tetrasulfide compound does not selectively break the central S–S bond, but regio-selectively generates trisulfide intermediates.

## Abstract (web summary)

The preparation of disulfides mainly relies on oxidative couplings of two sulfur-containing compounds, a strategy which has side reactions and other shortcomings. In this work, the authors present a reductive nickel-catalyzed cross-electrophile coupling of unactivated alkyl bromides with symmetrical tetrasulfides to form unsymmetrical disulfides, proceeding via trisulfide intermediates.

## Introduction

**Fig. 1:**
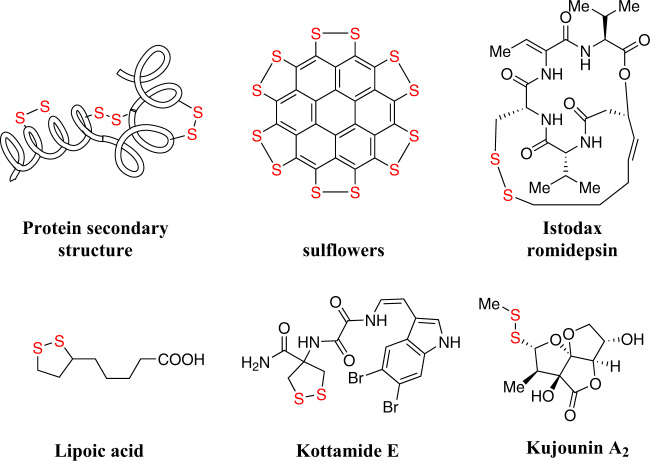
Representative biorelevant disulfide compounds. The importance of disulfides in life science, natural products, and other fields.

Disulfides are widely found in natural products and have a wide range of applications in many fields, such as life science^1–3^ and medical chemistry^4,5^. In the formation of protein tertiary structure, disulfide bonds play a central role^2,6,7^. The activity of many natural products and drugs depends on the exchange reaction of disulfide bonds in proteins^4,8,9^. The lipoic acid present in the mitochondria can increase the synthesis of ATP in the cell energy cycle^10^. Kottamide E has a five-membered ring disulfide structure, which has good anti-tumor and anti-inflammatory effects^11^. As a consequence, the efficient preparation of various functionalized disulfides is of continued interest (Fig. 1).

**Fig. 2:**
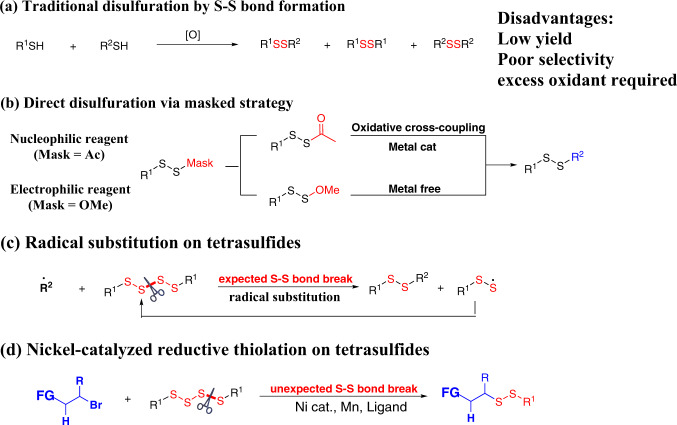
Strategies for unsymmetrical disulfide assembly. **a** Traditional disulfuration by S–S bond formation. **b** Direct disulfuration via masked strategy. **c** Radical substitution on tetrasulfides. **d** Nickel-catalyzed reductive thiolation on tetrasulfides.

Given the importance and predominance of disulfides in pharmaceuticals and bioactive compounds, chemists are continually seeking for efficient preparation strategies for multifunctional disulfides. While the preparation of symmetrical disulfide has been greatly developed^12^. Due to the limitation of the substrate structures, the preparation of unsymmetrical disulfide is still challenging. The traditional method for the construction of unsymmetrical disulfide compounds based on oxidative coupling of two different thiols (Fig. 2a)^13–17^. In view of low yields, poor selectivities, and poor substrate universality, the development of a more efficient method for preparing unsymmetrical disulfide is desirable. In recent years, chemists have designed and synthesized many prefunctionalized/activated disulfurating reagents, and successfully constructed a series of functionalized unsymmetrical disulfides. Xian^18^, Xu^19^, and Wang^20^ have independently disclosed their own disulfide reagents. In this field, Jiang’s group has made excellent achievement, by the introduction of the Mask group into the disulfide reagent, designed and synthesized a series of nucleophilic or electrophilic disulfide reagents, and efficiently constructed a series of highly functionalized polysulfide compounds (Fig. 2b)^21,22^. On the basis of these studies, they designed and synthesized a series of new bilateral disulfurating reagents, which greatly expanded the construction of unsymmetrical disulfide compounds^23,24^. In 2020, the Pratt group demonstrated that tetrasulfides undergo efficient homolytic substitution with alkyl radicals, enabling the synthesis of a broad range of unsymmetrical disulfides (Fig. 2c)^25–27^. This protocol showed mild reaction conditions and a large substrate scope.

Reductive coupling reactions of electrophiles avoid the preparation and use of organometallic reagents, have excellent compatibility with various functional groups, and provide an important method for the construction of C–C bonds^28^. In recent years, breakthroughs have been made in the research of this strategy, and a series of cross-coupling reactions involving C(sp^2^)-X and C(sp^3^)-X electrophiles have been realized^29–32^. In recent years, our group has used thiosulfonate as a sulfur source and constructed a series of highly functionalized sulfides and selenides under nickel-catalyzed reductive coupling conditions^33–35^. Inspired by Pratt’s work and combined with our previous work on reductive coupling reactions to synthesize sulfides, we reasoned that we can prepare unsymmetrical disulfides from alkyl halides through Nickel-catalyzed cross-electrophile coupling with tetrasulfide. Herein, we report on the successful development of a cross-coupling reaction of unactivated alkyl bromides with symmetrical tetrasulfides utilizing nickel-catalyzed reductive coupling strategy to construct unsymmetrical alkyl–alkyl disulfides and alkyl–aryl disulfides (Fig. 2d).

## Results

### Reaction conditions survey

Initially, we tried the reaction of 1-bromo-3-phenylpropane (**1b**) with 1,4-di-*tert*-butyltetrasulfane (**2a**), Ni(PPh_3_)_2_Cl_2_ (5 mol%), Mn (1.5 equiv), ligand (10 mol%) in (dry) DMF at 40 °C for 12 h under N_2_ atmosphere. Gratifyingly, the reaction proceeded smoothly to give a mixture products of 1-(*tert*-butyl)-2-(3-phenylpropyl)disulfane (**3b**) and 1-(*tert*-butyl)-3-(3-phenylpropyl)trisulfane (**3b**′) (2.8:1) (However, the disulfide could not be separated from the trisulfide). Surprisingly, when we extended the reaction time to 24 h, the ratio of **3b** and **3b**′ increased to 31:1. With this promising results in hand, we tried to further optimize the reaction conditions (see Supplementary Tables 1–3 for more details). As briefly illustrated in Table 1, after systematic exploring the reductive coupling with **L1** as the ligand, Ni(acac)_2_ was chosen as catalyst as its use lead to the highest yield and selectivity (Table 1, entries 1–5). Next, we conducted the ligand optimization with a series of bipyridine ligands and phenanthroline ligands. As a result, **L1** provided superior result (Table 1, entries 6–12). Then, we tried the reactions in several different solvents, such as DMF, DMA, DMSO, and MeCN. It was found that DMF was crucial for the successful transformation of **1b** (Table 1, entries 13–15). When Zn was used instead of Mn, the target product **3b** could not be obtained (Table 1, entry 16).

**Table 1:** Optimization of the reaction conditions^a,b^.

					
Entry	[Ni]	Ligand	Reductant	Solvent	3b-Yield (%)^b^
1	Ni(acac)_2_	**L1**	Mn	DMF	>99
2	Ni(PPh_3_)_2_Cl_2_	**L1**	Mn	DMF	78
3	NiCl_2_	**L1**	Mn	DMF	64
4	NiI_2_	**L1**	Mn	DMF	75
5	NiBr_2_	**L1**	Mn	DMF	85
6	Ni(acac)_2_	**L2**	Mn	DMF	>99
7	Ni(acac)_2_	**L3**	Mn	DMF	75
8	Ni(acac)_2_	**L4**	Mn	DMF	98
9	Ni(acac)_2_	**L5**	Mn	DMF	>99
10	Ni(acac)_2_	**L6**	Mn	DMF	85
11	Ni(acac)_2_	**L7**	Mn	DMF	90
12	Ni(acac)_2_	**L8**	Mn	DMF	99
13	Ni(acac)_2_	**L1**	Mn	DMA	20
14	Ni(acac)_2_	**L1**	Mn	MeCN	0
15	Ni(acac)_2_	**L1**	Mn	DMSO	0
16	Ni(acac)_2_	**L1**	Zn	DMF	Trace


^a^Reaction conditions: **1b** (0.2 mmol, 1.0 equiv.), **2a** (0.24 mmol, 1.2 equiv.), Ni(acac)_2_ (5.0 mol%), ligand (10 mol%); Mn (0.3 mmol, 1.5 equiv.), DMF (1 mL); N_2_ atmosphere; 40 °C; 24 h.

^b^Yields were determined by GC with biphenyl as the internal standard.

### Generality of protocol

**Fig. 3:**
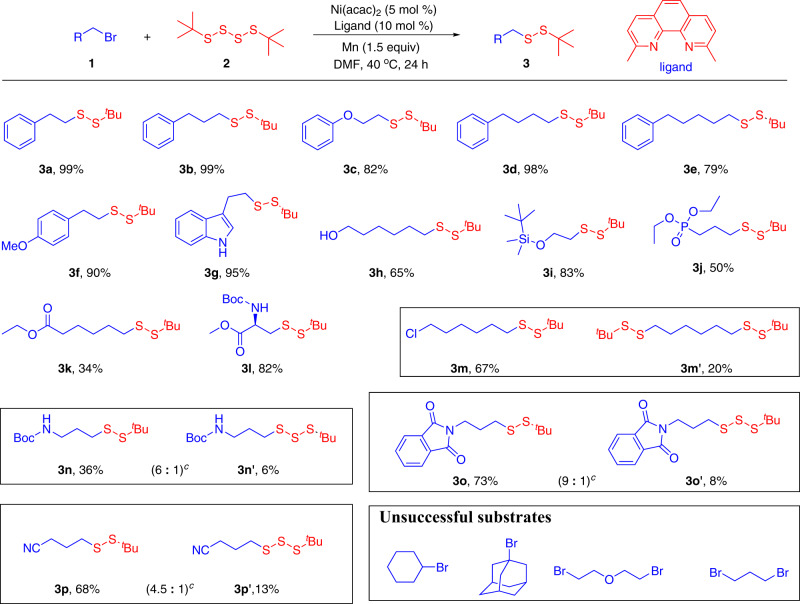
Scope of primary alkyl bromides. ^a^Reaction conditions: primary alkyl bromides **1** (0.20 mmol, 1.0 equiv.), **2a** (0.24 mmol, 1.2 equiv.), Ni(acac)_2_ (5.0 mol%); L1 (10 mol%), Mn (0.30 mmol, 1.5 equiv.), DMF (1 mL), N_2_ atmosphere, 40 °C, 24 h. ^b^Isolated yield. ^c^80 °C.

With the optimized reaction conditions in hand, we set out to assess the generality of this protocol (Fig. 3). Hence, primary alkyl bromides bearing various functional groups were employed in the reactions with 1,4-di-*tert*-butyltetrasulfane **2a**. To our delight, the length of the carbon chain had no effect on the reaction, the desired disulfide products **3a**–**3f** could be obtained in good to excellent yields. The reaction of 3-(2-bromoethyl)-1*H*-indole **1g** with 1,4-di-*tert*-butyltetrasulfane (**2a**) gave the target product **3g** in excellent yield. Satisfactorily, a wide range of substituents, including alcohol, siloxane, phosphonate were well-tolerated under this mild reaction condition. The target products **3h**–**3j** were isolated in medium to good yields. However, the reaction of ethyl 6-bromohexanoate **1k** with 1,4-di-*tert*-butyltetrasulfane (**2a**) gave the target product **3k** in only 34% yield. It should be noted that the reaction of methyl(*R*)-3-bromo-2-((*tert*-butoxycarbonyl)amino)propanoate (**1l**) with 1,4-di-*tert*-butyltetrasulfane (**2a**) furnished the desired product **3l** in 82% yield. This greatly expanded the application of this strategy in the construction of unsymmetrical disulfide compounds. Surprisingly, the reaction of 1-bromo-6-chlorohexane (**1m**) with tetrasulfane **2a** not only could delivered the expected product **3m** in 67% yield, but also led to **3m**′ in 20% yield. This demonstrated that this strategy is also applicable to alkyl chlorides. When *tert*-butyl (3-bromopropyl)carbamate (**1n**), 2-(3-bromopropyl)isoindoline-1,3-dione (**1o**) and 4-bromobutanenitrile (**1p)** were applied to the reaction respectively, a mixture of trisulfide compounds and disulfides were obtained (**3n**–**3p**, **3n**′–**3p**′). In contrast, the reaction of secondary bromide, tertiary bromide and dibromide failed to give the desired products. According to the related works of Prof. Gong^36–38^, we speculate that the possible reason is that the oxidative addition of nickel(0) and tetrasulfide furnishes nickel(II) intermediate, this intermediate is very sterically hindered due to the coordination of the ligand. Therefore, when secondary bromine and tertiary bromine participate in the reaction, the target products could not be obtained due to the steric hindrance effect.

**Fig. 4:**
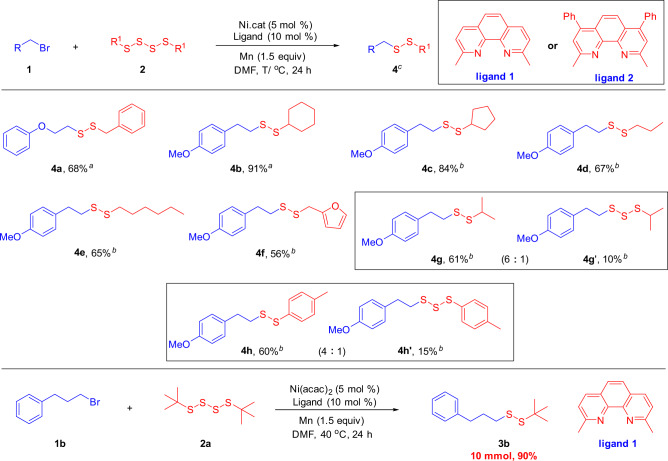
Scope of tetrasulfides 2. Reaction conditions: Primary alkyl bromides **1** (0.20 mmol, 1.0 equiv.), symmetrical tetrasulfides **2** (0.24 mmol, 1.2 equiv.), Mn (0.30 mmol, 1.5 equiv.), DMF (1 mL), N_2_ atmosphere. ^a^Ni(acac)_2_ (5.0 mol%), **L1** (10 mol%); 40 °C, 24 h. ^b^NiF_2_ (5.0 mol%), **L2** (10 mol%), 80 °C; 24 h. ^c^Isolated yield.

Encouraged by the above achievements, we turned our attention to exploring other symmetrical tetrasulfide compounds (Fig. 4). However, under the previously optimized conditions, only 1,4-dibenzyltetrasulfane and 1,4-dicyclohexyltetrasulfane gave the target products **4a** and **4b** with a high selectivity. When other tetrasulfides were applied to the reaction, a mixture products of trisulfide and tetrasulfide was obtained. Therefore, we adjusted the reaction conditions (see Supplementary Tables 4–6 for more details). Next, we reexamined the activity of various symmetric tetrasulfide substrates. It is exciting that regardless of whether it is branched liner tetrasulfides, the desired products could be obtained with high chemoselectivity (**4c**–**4e**). The reaction of 1-(2-bromoethyl)-4-methoxybenzene with 1,2-bis(furan-2-ylmethyl)disulfane gave the desired product **4f** in moderate yield. However, when 1,4-diisopropyltetrasulfane and 1,4-di-*p*-tolyltetrasulfane were applied to the reaction, respectively, a mixture product of trisulfide and disulfide were formed (**4g**–**4h**, **4g**′–**4h**′). To demonstrate the synthetic value of our method, we explored the scale-up with (3-bromopropyl)benzene (**1b**) and 1,4-di*-tert*-butyltetrasulfane (**2a**) under the otherwise identical reaction conditions. To our delight, the desired product **3b** could be obtained in 90% yield.

### Mechanistic studies

**Fig. 5:**
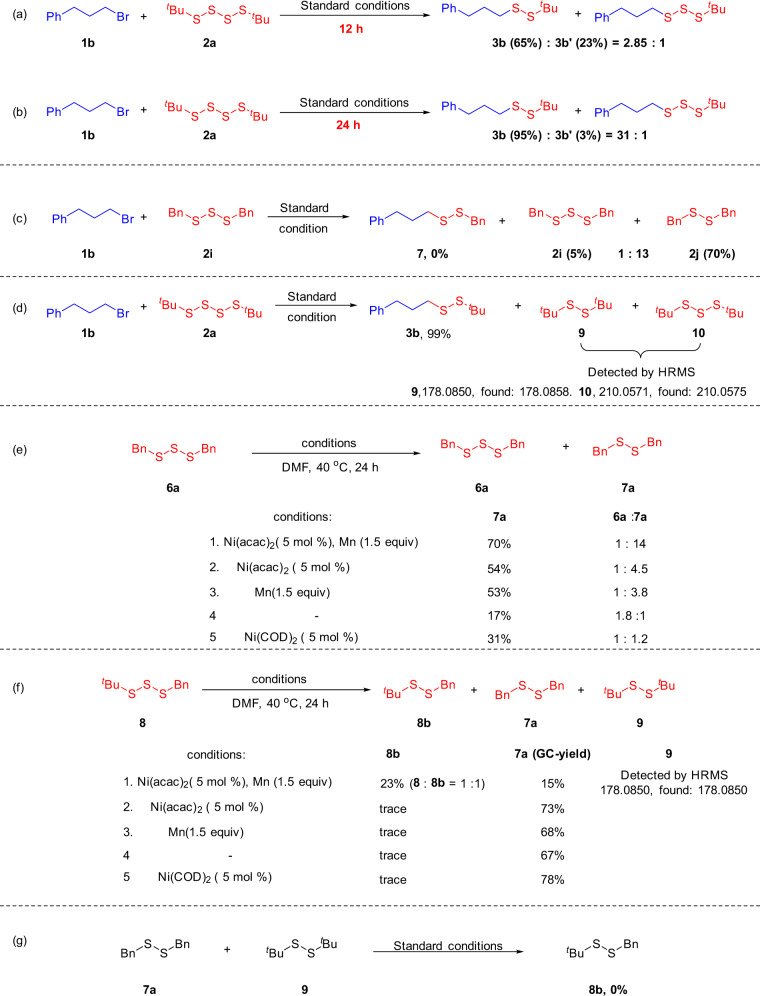
Key mechanistic findings. **a** The reaction of **1b** and **2a** under standard conditions for 12 h. **b** The reaction of **1b** and **2a** under standard conditions for 24 h. **c** The reaction of **1b** and **2j** under standard conditions for 24 h. **d** The catalytic system was analyzed by high-resolution mass spectrometry. **e** Investigation on the reaction mode of trisulfide **6a** under different conditions. **f** Investigation on the reaction mode of unsymmetrical trisulfide **8** under different conditions. **g** The reaction of 1,2-dibenzyldisulfane **7a** and 1,2-di-tert-butyldisulfane **9** under standard conditions for 24 h.

Subsequently, mechanistic studies were conducted to gain insights into the reaction pathway (see Supplementary Figs. 1–3 for more details). When Ni(PPh_3_)_2_Cl_2_ was applied as the catalyst, the reaction proceeded to give a mixture products of **3b** and **3b**′ (2.8:1) (Fig. 5a). Surprisingly, when we extended the reaction time to 24 h, the ratio of **3b** and **3b**′ changed 31:1 (Fig. 5b). This indicated that product **3b**′ might be a reaction intermediate. Therefore, we prepared the trisulfide product **2i**, regrettably, the reaction of (3-bromopropyl)benzene (**1b**) with 1,3-dibenzyltrisulfane (**2i**) did not give the target product **7**, but the trisulfide product **3i** itself was converted into disulfide compound **2j** (**2i**:**2j** = 1:13) (Fig. 5c). This finding indicated that trisulfide is more likely to transform into disulfide.

Next, we performed high-resolution mass spectrometry to analyse the catalytic system. Under the standard conditions, only trace amount of the disulfide product **9** and the trisulfide product **10** were detected by careful HRMS analysis (Fig. 5d). We likewise investigated the viability for a conversion of trisulfides to disulfides (Fig. 5e) (see Supplementary Figs. 4, 5 and Table 7 for more details). Through detailed experimentation, it was demonstrated that both the nickel catalyst and the manganese play a crucial role in the conversion of trisulfides to disulfides. According to a previous study^19^, we furthermore prepared unsymmetric trisulfide **8**. Under standard conditions, 1-benzyl-2-(tert-butyl)disulfane **8b** and 1,2-dibenzyldisulfane **7a** could be obtained in 23% and 15% yields, respectively (with a conversion of **8** being 77%). Only trace amounts of the disulfide product **9** was detected by HRMS analysis. We investigated the conditions for the conversion of trisulfide **8** to disulfide **8b**. It was found that when the reaction conditions were modified, only trace amounts of unsymmetric disulfide **8b** and symmetric disulfide **9** were detected and **7a** was formed as the main product (Fig. 5f). We further investigated the cross reaction of 1,2-dibenzyldisulfane **7a** and 1,2-di-*tert*-butyldisulfane **9** under otherwise identical reaction conditions, which notably failed to give unsymmetrical disulfide **8b**. This result provides strong support against an initial formation of two symmetrical disulfides from the trisulfide. Therefore, a subsequent cross reaction to form the unsymmetric disulfide is unlikely to be operative (Fig. 5g).

**Fig. 6:**
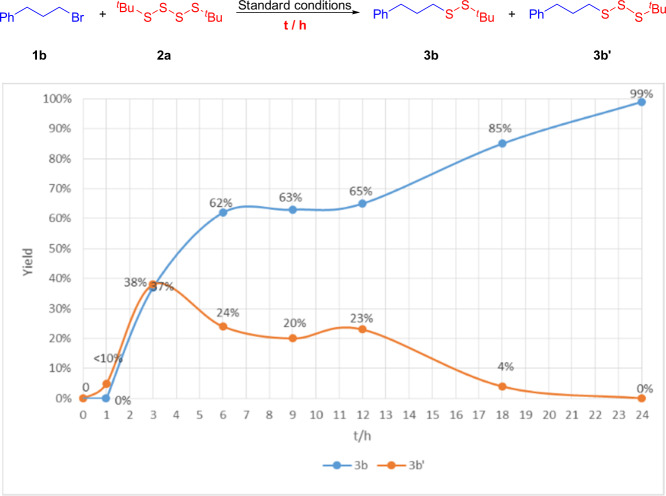
Investigation of the reaction process. The investigation of the template reaction under standard conditions at different time by GC-Ms.

Subsequently, we investigated the standard reaction at different reaction times (Fig. 6). Through detailed GC-MS analysis, we found that when the reaction proceeded for 1 h, **3b** could not be detected, while instead only the trisulfide product **3b**′ was detected. When the reaction time was prolonged to 3 h, the ratio of products **3b** and **3b**′ was close to 1:1. Thereafter, the yield of **3b′** gradually decreased. These experimental findings render the possibility of nickel inserting into the central S–S bond of the tetrasulfide unlikely. Instead our observations provide further strong support for trisulfide serving as the key intermediate.

**Fig. 7:**
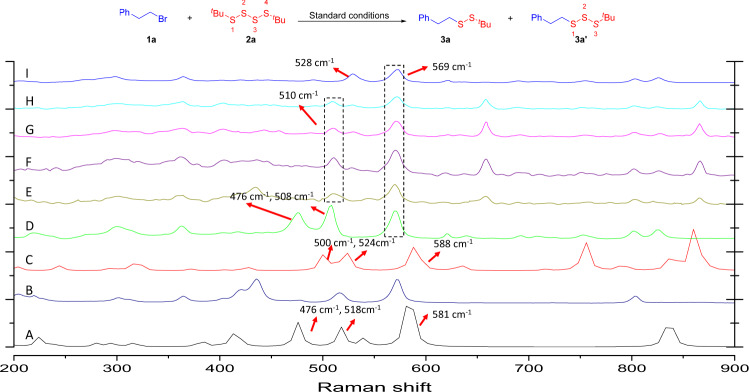
Raman study (I). Raman caculation spectra of (A) **2a** calculated data. (B) **2a**. (C) **3a**′ calculated data. (D) **3a**′. (E) **1a** with **2a** under optimized reaction conditions, *t* = 1 h, (F) **1a** with **2a** under optimized reaction conditions, *t* = 4 h, (G) **1a** with **2a** under optimized reaction conditions, *t* = 8 h, (H) **1b** with **2a** under optimized reaction conditions, *t* = 1 h. (I) **3a**.

**Fig. 8:**
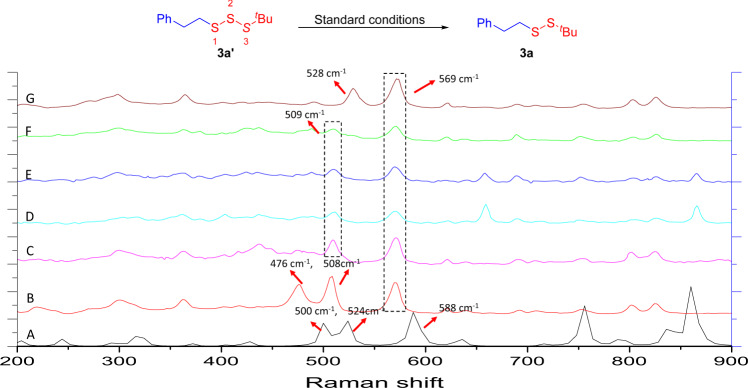
Raman study (II). Raman spectra of (A) **3a**′ calculated data, (B) **3a**′. (C) **3a**′ under optimized reaction conditions, *t* = 1 h, (D) **3a**′ under optimized reaction conditions, *t* = 4 h, (E) **3a**′ under optimized reaction conditions, *t* = 8 h, (F) **3a**′ under optimized reaction conditions, *t* = 12 h, (G) **3a**.

There is increasing literature precedence for S–S bonds in polysulfides serving as strong Raman scatterers^39^. Thus, we exploited the enormous enhancement in the Raman intensity of polysulfides in order to understand the working mode of our reductive coupling. To aid the assignment, Raman frequency calculations for **2a** (Fig. 7, A), **3a** (Fig. 7, I), and **3a**′ (Fig. 7, C) were carried out at the UB3LYP/aug-cc-pV(Q + d)Z-DK level. Thus, Raman frequency calculations for **2a** featured peaks at 476 cm^−1^, 518 cm^−1^, and 581 cm^−1^ corresponding to the S(2)–S(3), S(1)–S(2), and (^*t*^Bu)C-S stretching of tetrasulfide, respectively. As Raman frequency calculations for **3a**′, peaks at 500 cm^−1^, 524 cm^−1^, and 588 cm^−1^ correspond to the S(1)–S(2), S(2)–S(3), and (^*t*^Bu)C-S stretching of trisulfide **3a**′, respectively. As Raman frequency for **3a**′, peaks at 476 cm^−1^, 508 cm^−1^, and 569 cm^−1^ correspond to the S(1)–S(2), S(2)–S(3), and (^*t*^Bu)C-S stretching of trisulfide **3a**′, respectively. This result is most likely match with the calculated data with −24 cm^−1^ relative error. As Raman frequency for **3a**, peaks at 528 cm^−1^, and 569 cm^−1^ correspond to the S–S, and C-S stretching of disulfide **3a**, respectively. We further monitored the reaction of **1a** and **2a** under the standard reaction conditions with the Raman spectroscopy (Fig. 7, E–H). It was found that 476 cm^−1^ correspond to the S(2)–S(3) of tetrasulfide **2a** decreased and a significant increase in 528 cm^−1^ peak (*ν*_1_), which is the characteristic S–S stretching of **3a**. We also investigated the Raman spectroscopy of **3a**′ under the standard reaction conditions (Fig. 8, C–F). It was found that peak at 476 cm^−1^ and 508 cm^−1^ corresponds to the S(1)–S(2) and S(2)–S(3) of trisulfide **3a**′ decreased dramatically. This result means that S–S–S bond of trisulfide **3a**′ could be quickly activated under the reaction conditions.

**Fig. 9:**
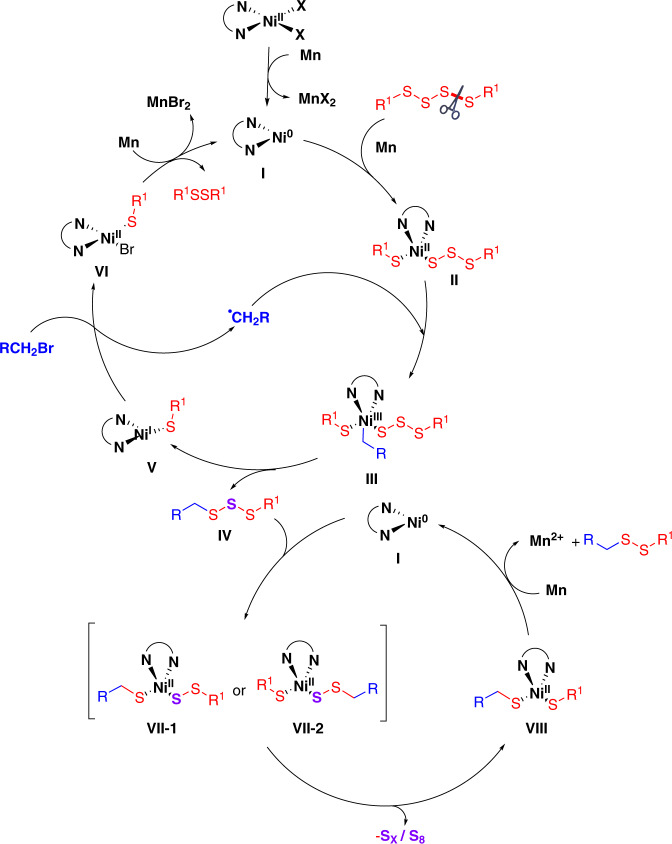
Plausible catalytic cycle. Possible reaction mechanism for nickel-catalyzed reduction of disulfide.

Based on our detailed experimental and computational findings and the literature reports^33–35,40–42^, we propose a plausible reaction mechanism in Fig. 9. The in situ reduction of nickel(II) by manganese affords Ni(0)(L)_2_ **I**. The tetrasulfide **2** could be activated by Ni(0) or Mn. The oxidative addition of **I** and tetrasulfide **2** in the presence of Ni(0) or Mn furnishes intermediate **II**, which reacts with an alkyl radical to form nickel(III)(L) intermediate **III**. The reductive elimination of **III** leads to trisulfide intermediate **IV** and the nickel(I)(L)_2_ species **V**. Next, the nickel(I) **V** reacts with alkyl bromide **1** to give the alkyl radical and nickel(II)(L) **VI**. Finally, the reduction of intermediate **VI** regenerates complex **I** and disulfide by-product. On the other hand, the oxidative addition of **I** and trisulfide **IV** in the presence of Ni(0) affords intermediate **VII** (both intermediates **VII-1** and intermediate **VII-2** are possible). **VII** is converted into intermediate **VIII** with releasing polysulfur anion and S_8_ (polysulfur anion and S_8_ were confirmed by XRD. For details, please see Supplementary Fig. 5). The reductive elimination of **VIII** furnish disulfide and complex **I**.

## Discussion

In conclusion, we have reported on a nickel-catalyzed reductive disulfideization of unactivated alkyl bromides with organic tetrasulfides. The reaction proceeded with excellent chemoselectivity under mild conditions, and enabled the efficient assembly of diversely decorated disulfides. Detailed mechanistic studies revealed that, in contrast to literature precedence, the tetrasulfide compound did not undergo scission of the central S–S bond, but selectively formed substituted trisulfides as key intermediates.

## Methods

### General procedures

#### Synthesis of 3b

In a glovebox, an oven-dried screw-capped 8 mL vial equipped with a magnetic stir bar was charged with (3-bromopropyl)benzene (**1b**, 39.6 mg, 0.2 mmol) and 1,4-di-*tert*-butyltetrasulfane (**2a**, 58.1 mg 0.24 mmol), Ni(acac)_2_ (2.6 mg, 5.0 mol%), L1 (10 mol%), Mn (1.5 equiv), DMF (1.0 mL) was added via syringe. The reaction mixture was stirred for 24 h at 40 °C. After 24 h, the crude reaction mixture was diluted with ethyl acetate (20 mL) and washed with water (20 mL × 3). The organic layer was dried over Na_2_SO_4_, filtered, and concentrated. The residue was purified by flash chromatography to afford pure product **3b** (99% yield).

#### Synthesis of 4h

In a glovebox, an oven-dried screw-capped 8 mL vial equipped with a magnetic stir bar was charged with 1-(2-bromoethyl)-4-methoxybenzene (**1f**, 42.8 mg, 0.2 mmol) and 1,4-di-*p*-tolyltetrasulfane (**2h**, 74.4 mg 0.24 mmol), NiF_2_ (1.0 mg, 5.0 mol%), L1 (10 mol%), Mn (1.5 equiv), DMF (1.0 mL) was added via syringe. The reaction mixture was stirred at 80 °C for 24 h. After 24 h, the crude reaction mixture was diluted with ethyl acetate (20 mL) and washed with water (20 mL × 3). The organic layer was dried over Na_2_SO_4_, filtered, and concentrated. The residue was purified by flash chromatography to afford products of **4h** and **4h**′.

## Supplementary information

Supplementary Information

## Data Availability

The authors declare that all other data supporting the findings of this study are available within the article and Supplementary Information files, and also are available from the corresponding author on request.
