# Supplementary material for: Efficient preparation of unsymmetrical disulfides by nickel-catalyzed reductive coupling strategy
Source: Nat Commun. 2022 May 11;13:2588. doi: 10.1038/s41467-022-30256-0 (PMC9095708; doi:10.1038/s41467-022-30256-0)
Supplement: Supplementary file 1 — Supplementary Information [file 41467_2022_30256_MOESM1_ESM.pdf]

Supplementary Information for

Efficient Preparation of Unsymmetrical Disulfides by Nickel-catalyzed Reductive Coupling Strategy

Fei Wang[a], Ying Chen[a], Weidong Rao,[b] Lutz Ackermann[c,d,]* and Shun-Yi Wang[a,]*

[a]*Key Laboratory of Organic Synthesis of Jiangsu Province, College of Chemistry, Chemical Engineering and Materials Science, Collaborative Innovation Center of Suzhou Nano Science and Technology, Soochow University, Suzhou 215123, China*
E-mail: shunyi@suda.edu.cn
[b] *Key Laboratory of Biomass-based Green Fuels and Chemicals, College of Chemical Engineering, Nanjing Forestry University, Nanjing 210037, China*
[c]*Institut für Organische und Biomolekulare Chemie Georg-August-Universität Göttingen Tammannstraße 2, 37077 Göttingen (Germany). E-mail: Lutz.Ackermann@chemie.uni-goettingen.de.*
[d]*Wöhler Research Institute for Sustainable Chemistry Georg-August-Universität Göttingen Tammannstraße 2, 37077 Göttingen (Germany).*

Supplementary Methods

1. General Methods

Unless otherwise noted, all commercially available compounds were used as provided without further purification. Solvents for chromatography were analytical grade and used without further purification. Anhydrous DMF, was purchased from Beijing InnoChem Science & Technology Co., Ltd. Analytical thin-layer chromatography (TLC) was performed on silica gel, visualized by irradiation with UV light. For column chromatography, 300-400 mesh silica gel was used. ^{1}H-NMR and ^{13}C-NMR were recorded on a BRUKER 400 MHz spectrometer in $CDCl_3$. Chemical shifts (δ) were reported referenced to an internal tetramethylsilane standard or the $CDCl_3$ residual peak (δ 7.26) for ^{1}H NMR. Chemical shifts of ^{13}C NMR are reported relative to $CDCl_3$ (δ 77.16). Data are reported in the following order: chemical shift (δ) in ppm; multiplicities are indicated s (singlet), bs (broad singlet), d (doublet), t (triplet), m (multiplet); coupling constants (J) are in Hertz (Hz). IR spectra were recorded on a BRUKER VERTEX 70 spectrophotometer and are reported in terms of frequency of absorption (cm^{-1}). HRMS spectra were obtained by using BRUKER micrOTOF-Q III instrument with ESI source. The starting materials were isolated by SepaBean machine Flash Chromatography, which was purchased from Santai Technologies Inc.

2. Synthesis of Substrates

General procedure for the synthesis of Di-*alkyl*-butyltetrasulfide.[1]

A solution of S_2Cl_2 (2.21 mL, 27.7 mmol) in dry ether (70 mL) is cooled to -78 °C in a dry ice/acetone bath. A solution of alkylthiol (6.25 mL, 55.4 mmol) and Et_3N (7.68 mL, 55.4 mmol) in dry ether (70 mL) is added dropwise over 1 hour. After the addition is complete, the solution is stirred at -78 °C for an additional 30 minutes after which is warmed to room temperature and quenched with water. The organic layer was separated and washed with water, Na_2CO_3 (sat.) and brine, dried over $MgSO_4$, filtered and concentrated in vacuo. The crude yellow oil was purified by column chromatography using hexanes as the eluent to yield the product as a yellow oil.

General Procedure for the synthesis of aryl tetrasulfides [2]**.**

The desired thiol (1.0 equiv.) and pyridine (1.0 equiv.) were added to anhydrous solvent (30 mL) in an oven-dried round bottom flask under nitrogen. The solution was cooled to -78 °C for 1 hour, after which sulfur monochloride (0.6 equiv.) was added dropwise. A white precipitate formed within seconds, and the reaction was stirred for 2 hours and then allowed to warm to room temperature. The reaction was quenched with deionized water (30 mL), and the aqueous layer was discarded. The organic layer was washed with deionized water (30 mL) and brine (30 mL). The organic layer was dried over sodium sulfate, filtered, and evaporated under reduced pressure to afford the pure product.

Supplementary Notes

1. Optimize reaction conditions for the synthesis of 3b.

Supplementary table 1. Optimization of Nickel-catalysts for the synthesis of **3b**[a,b]

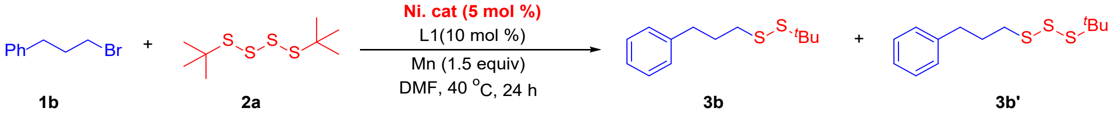

entry	cat（5 mol %）	Internal standard：3b：3b’
1[c]	$Ni(PPh_3)_2Cl_2$	55.78 : 32.58 : 11.64 (2.8:1)
2	$Ni(PPh_3)_2Cl_2$	53.18 : 45.36 : 1.46 (31:1)
3	$NiCl_2$	54.63 : 38.70 : 6.66 (6.0:1)
4	NiI_2	49.08 : 50.50 : 0.42 (120:1)
5	$NiBr_2$	45.10 : 54.75: 0.15 (384:1)
6	$NiCl_2{\cdot}DME$	49.92 : 48.25 : 1.83 (27:1)
7	$Ni(acac)_2$	47.72 : 52.28 : 0 (100%)(99%)[d]

[a] Reaction conditions: **1b** (0.2 mmol, 1.0 equiv.); **2a** (0.24 mmol, 1.2 equiv.); [Ni] (5.0 mol %); ligand (10 mol %); Mn (0.3 mmol, 1.5 equiv.); DMF (1 mL); N_2 atmosphere; 40 °C; 24 h. [b]Yields were determined by GC with Biphenyl as the internal standard. [c]12h. [d] Isolated Yield.

Supplementary table 2. Optimization of ligands for the synthesis of **3b**[a,b]

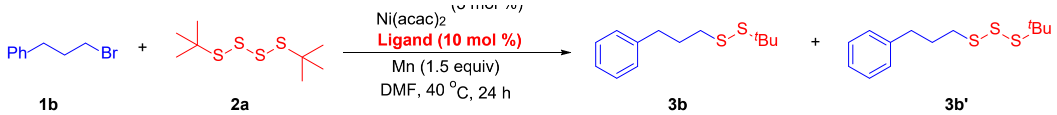

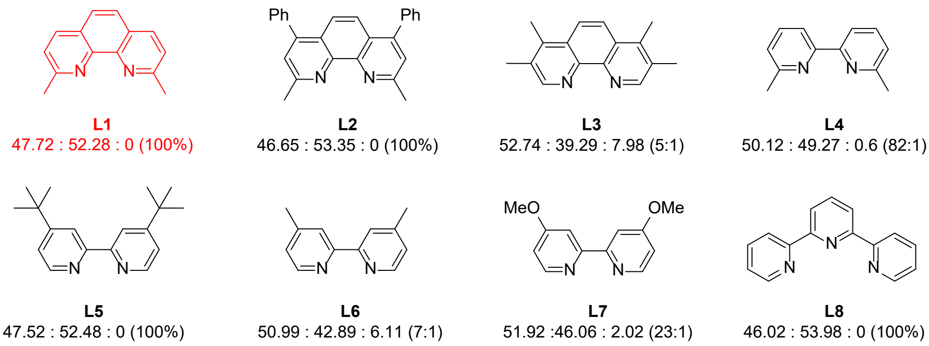

[a] Reaction conditions: **1b** (0.2 mmol, 1.0 equiv.); **2a** (0.24 mmol, 1.2 equiv.); $Ni(acac)_2$ (5.0 mol %); ligand (10 mol %); Mn (0.3 mmol, 1.5 equiv.); DMF (1 mL); N_2 atmosphere; 40 °C; 24 h. [b]Yields were determined by GC with Biphenyl as the internal standard.

Supplementary table 3. Control experiments of **3b**

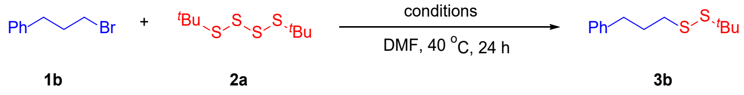

entry	conditions	3b
**1**	Ni(acac)$_2$ (5 mol %), L1 (10 mol %)	trace
**2**	Ni(acac)$_2$ (5 mol %), Mn (1.5 equiv)	trace
**3**	L1 (10 mol %), Mn (1.5 equiv)	trace
**4**	Ni(acac)$_2$ (5 mol %)	trace
**5**	L1 (10 mol %)	n.r
**6**	Mn (1.5 equiv)	trace

2. Optimize reaction conditions for the synthesis of 4h.

Supplementary table 4. Optimization of Nickel-catalysts for the synthesis of **4h**[a,b]

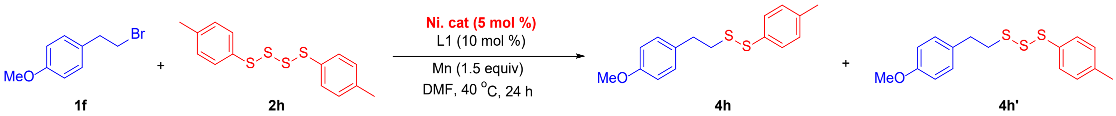

entry	cat（5 mol%）	Internal standard：4h：4h’
**1**	Ni(PPh$_3$)$_2$Cl$_2$	44.91 : 44.36 : 10.73 (4.1:1)
**2**	NiCl$_2$	49.39 : 39.66 : 10.96 (3.6:1)
**3**	NiI$_2$	48.85 : 40.46 : 10.68 (3.8:1)
**4**	NiBr$_2$	51.12 : 39.78: 9.100 (4.4:1)
**5**	Ni(ClO$_4$)$_2$·6H$_2$O	51.01 : 39.98 : 9.01 (4.4:1)
**6**	NiF$_2$	52.50 : 39.82 : 7.68 (5.2:1)

[a] Reaction conditions: **1f** (0.2 mmol, 1.0 equiv.); **2h** (0.24 mmol, 1.2 equiv.); [Ni] (5.0 mol %); L1 (10 mol %); Mn (0.3 mmol, 1.5 equiv.); DMF (1 mL); N_2 atmosphere; 40 °C; 24 h. [b]Yields were determined by GC with Biphenyl as the internal standard.

Supplementary table 5. Optimization of ligands for the synthesis of **4h**[a,b]

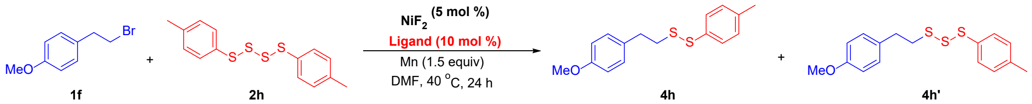

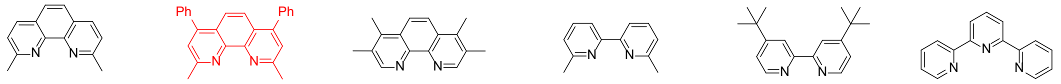

L1	L2	L3	L4	L5	L6
52.50 : 39.82 : 7.68 (5.2:1)	50.10 : 42.50 : 6.81 (6.2:1)	52.81 : 37.98 : 9.21 (4.1:1)	49.95 : 39.55 : 10.50(3.8:1)	52.05 : 37.65 : 10.30 (3.7:1)	51.52 : 37.88 : 10.61 (3.6:1)

[a]Reaction conditions: **1f** (0.2 mmol, 1.0 equiv.); **2h** (0.24 mmol, 1.2 equiv.); NiF_2 (5.0 mol %); Ligand (10 mol %); Mn (0.3 mmol, 1.5 equiv.); DMF (1 mL); N_2 atmosphere; 40 °C; 24 h. [b]Yields were determined by GC with Biphenyl as the internal standard.

Supplementary table 6. Optimization of solvents for the synthesis of **4h**[a,b]

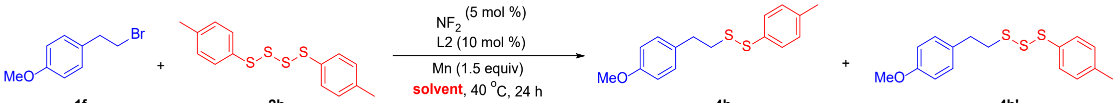

entry	solvent	Internal standard：4h：4h’
1	DMF	50.10 : 42.50 : 6.81 (6.2:1)
2	DMA	42.74 : 42.22 : 15.05 (2.8:1)
3	DMSO	51.67 : 38.56 : 9.77 (3.9:1)
4	MeCN	N.R
5	DCE	N.R
6[c]	DMF	60% : 15% (4:1)

[a] Reaction conditions: **1f** (0.2 mmol, 1.0 equiv.); **2h** (0.24 mmol, 1.2 equiv.); NiF_2 (5.0 mol %); Ligand (10 mol %); Mn (0.3 mmol, 1.5 equiv.); solvent (1 mL); N_2 atmosphere; 40 °C; 24 h. [b]Yields were determined by GC with Biphenyl as the internal standard. [c] 80 °C; Isolated Yield.

Note 1: After optimization of the conditions, we found that the selectivity of products 4h and 4h’ is the best when the reaction is at 80 °C. However, when investigating the universality of the substrates, we found that the selectivities of alkyl tetrasulfides are not good when reacting at 40 °C. Happily, the disulfide products could be obtained with a single selectivity at 80 °C, Therefore, we chose to investigate the universality of the substrates at 80 °C.

Note 2: For product 3b’, we also tried to optimize the reaction conditions. Unfortunately, the yield and selectivity are not good. Therefore, we abandoned the investigation of product 3b’.

3. General Procedure and Product Characterization

3.1 General Procedure A

A representative procedure synthesis of 1-(tert-butyl)-2-(3-phenylpropyl)disulfane (**3b**) is shown below.

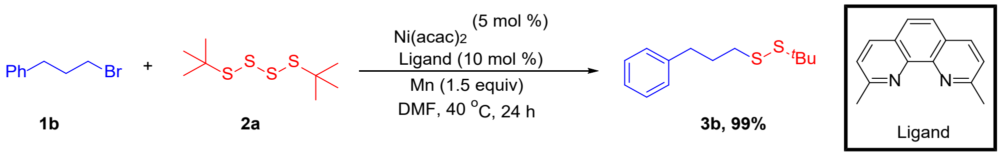

In glovebox, an oven-dried screw-capped 8 mL vial equipped with a magnetic stir bar was charged with (3-bromopropyl)benzene **1b** (39.6 mg, 0.2 mmol) and 1,4-di-*tert*-butyltetrasulfane **2a** (58.1 mg 0.24 mmol), Ni(acac)$_2$ (2.6 mg, 5.0 mol %), Ligand (10 mol %), Mn (1.5 equiv), DMF (1.0 mL) was added via syringe. The reaction mixture was stirred for 24 h at 40 °C. After 24 h, the crude reaction mixture was diluted with ethyl acetate (20 mL) and washed with water (20 mL × 3). The organic layer was dried over Na_2SO_4, filtered, and concentrated. The residue was purified by flash chromatography to afford pure product **3b** (99% yield).

A representative procedure synthesis of 1-(4-methoxyphenethyl)-2-(p-tolyl)disulfane (**4h**) is shown below.

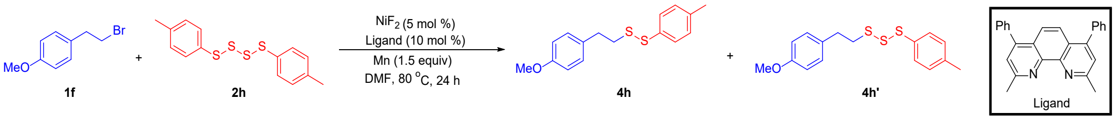

In glovebox, an oven-dried screw-capped 8 mL vial equipped with a magnetic stir bar was charged with 1-(2-bromoethyl)-4-methoxybenzene **1f** (42.8 mg, 0.2 mmol) and 1,4-di-*p*-tolyltetrasulfane **2h** (74.4 mg 0.24 mmol), NiF_2 (1.0 mg, 5.0 mol %), Ligand (10 mol %), Mn (1.5 equiv), DMF (1.0 mL) was added via syringe. The reaction mixture was stirred at 80 °C for 24 h. After 24 h, the crude reaction mixture was diluted with ethyl acetate (20 mL) and washed with water (20 mL × 3). The organic layer was dried over Na_2SO_4, filtered, and concentrated. The residue was purified by flash chromatography to afford mixture products of **4h** and **4h'**.

3.2 General Procedure B

The procedure scale-up synthesis of **3b** is shown below.

In glovebox, An oven-dried screw-capped 50-mL vial equipped with a magnetic stir bar was charged with (3-bromopropyl)benzene **1b** (10 mmol) and 1,4-di-*tert*-butyltetrasulfane **2a** (12 mmol**)**, Ni(acac)$_2$ (5.0 mol %), Ligand (10 mol %), Mn (1.5 equiv), DMF was added via syringe. The reaction mixture was stirred for 24 h at 40 °C. After 24 h, the crude reaction mixture was diluted with ethyl acetate (20 mL) and washed with water (20 mL × 3). The organic layer was dried over Na_2SO_4, filtered, and concentrated. The residue was purified by flash chromatography to afford pure product (90% yield).

Supplementary Discussion

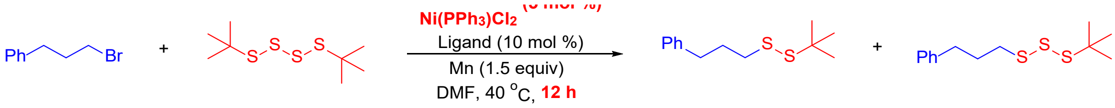

1a **2a** **3b : 3b' = 2.85 : 1**

3b, HRMS (ESI^{+}, MeCN) m/z calcd for $C_{13}H_{21}S_2$ (M+H)+: 241.1085, found 241.1085

3b', HRMS (CI) m/z (M^{+}) calcd for $C_{13}H_{21}S_3$: 272.0727, found 272.0727

NMR of a mixture compound of **3b** and **3b'** (400 MHz, room temperature, CDCl3).

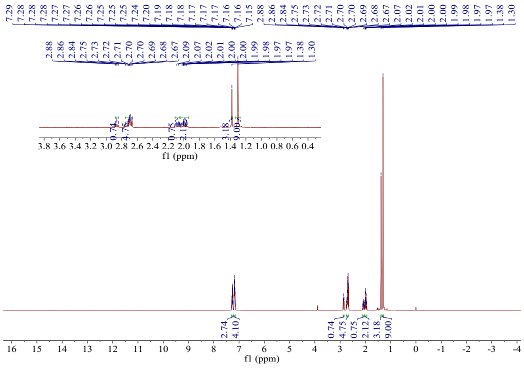

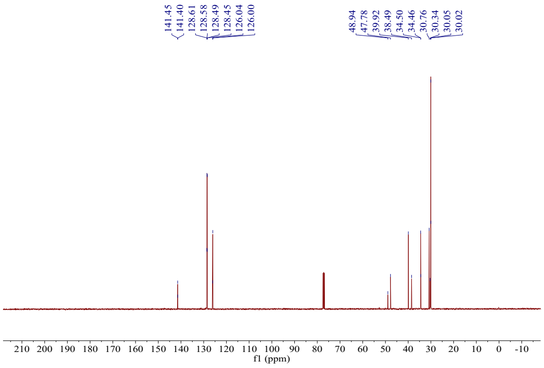

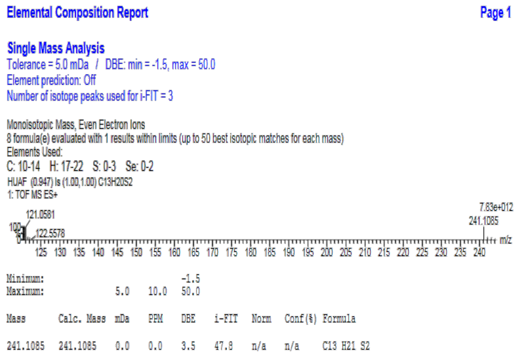
Elemental Composition Report

Page 1

Single Mass Analysis
Tolerance = 5.0 mDa / DBE: min = -1.5, max = 50.0
Element prediction: Off
Number of isotope peaks used for i-FIT = 3

Monoisotopic Mass, Even Electron Ions
8 formula(e) evaluated with 1 results within limits (up to 50 best isotopic matches for each mass)
Elements Used:
C: 10-14 H: 17-22 S: 0-3 Se: 0-2
HUAF (0.947) Is (1.00,1.00) C13H20S2
1: TOF MS ES+

Minimum: -1.5
Maximum: 5.0 10.0 50.0

Mass	Calc. Mass	mDa	PPM	DBE	i-FIT	Norm	Conf(%)	Formula
241.1085	241.1085	0.0	0.0	3.5	47.8	n/a	n/a	C13 H21 S2

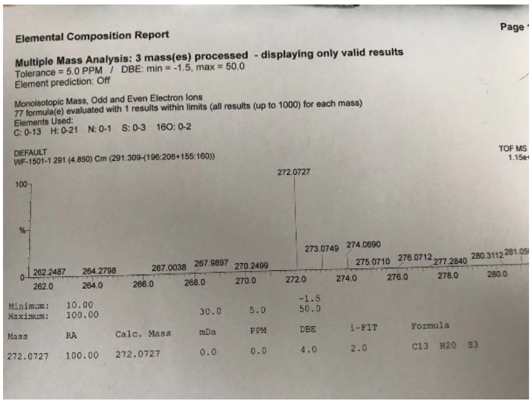
Elemental Composition Report

Multiple Mass Analysis: 3 mass(es) processed - displaying only valid results
Tolerance = 5.0 PPM / DBE: min = -1.5, max = 50.0
Element prediction: Off

Monoisotopic Mass, Odd and Even Electron Ions
77 formula(e) evaluated with 1 results within limits (all results (up to 1000) for each mass)
Elements Used:
C: 0-13 H: 0-21 N: 0-1 S: 0-3 16O: 0-2

Minimum: 10.00 -1.5
Maximum: 100.00 30.0 5.0 50.0

Mass	RA	Calc. Mass	mDa	PPM	DBE	i-FIT	Formula
272.0727	100.00	272.0727	0.0	0.0	4.0	2.0	C13 H20 S3

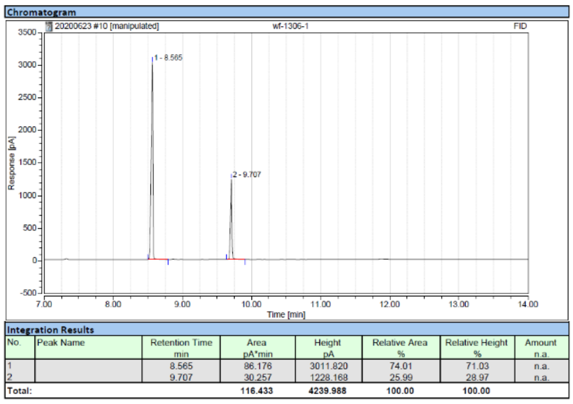

Integration Results

No.	Peak Name	Retention Time min	Area pA*min	Height pA	Relative Area %	Relative Height %	Amount n.a.
1		8.565	86.176	3011.820	74.01	71.03	n.a.
2		9.707	30.257	1228.168	25.99	28.97	n.a.
Total:			116.433	4239.988	100.00	100.00	

Supplementary figure 1. (3-bromopropyl)benzene **1b** (39.6 mg, 0.2 mmol), 1,4-di-*tert*-butyltetrasulfane **2a** (58.1 mg 0.24 mmol), $Ni(PPh_3)_2Cl_2$ (2.6 mg, 5.0 mol %), Ligand (10 mol %), Mn (1.5 equiv), DMF (1.0 mL). **12 h**, 40 ºC. After **12 h**, a mixture of product **3b** and **3b' (2.85 : 1)** was obtained. **(The disulfide could not be separated from the trisulfide, they were an inseparable mixture and that the yields are by NMR.)**

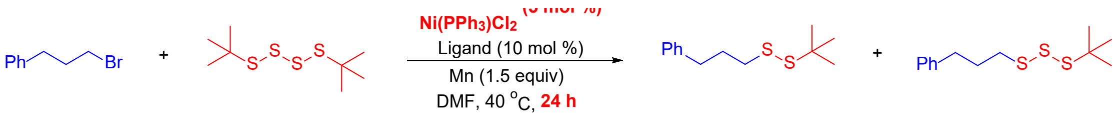

1a **2a** **3b : 3b' = 31 : 1**

NMR of compound **3b** (400 MHz, room temperature, CDCl3).

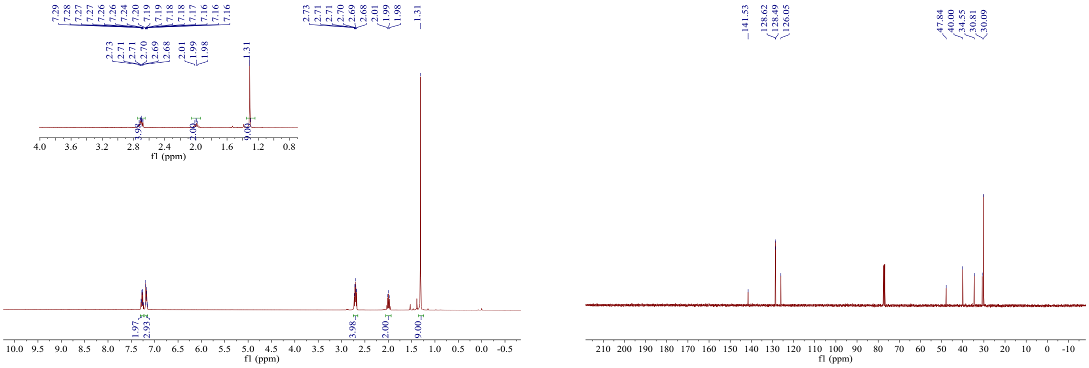

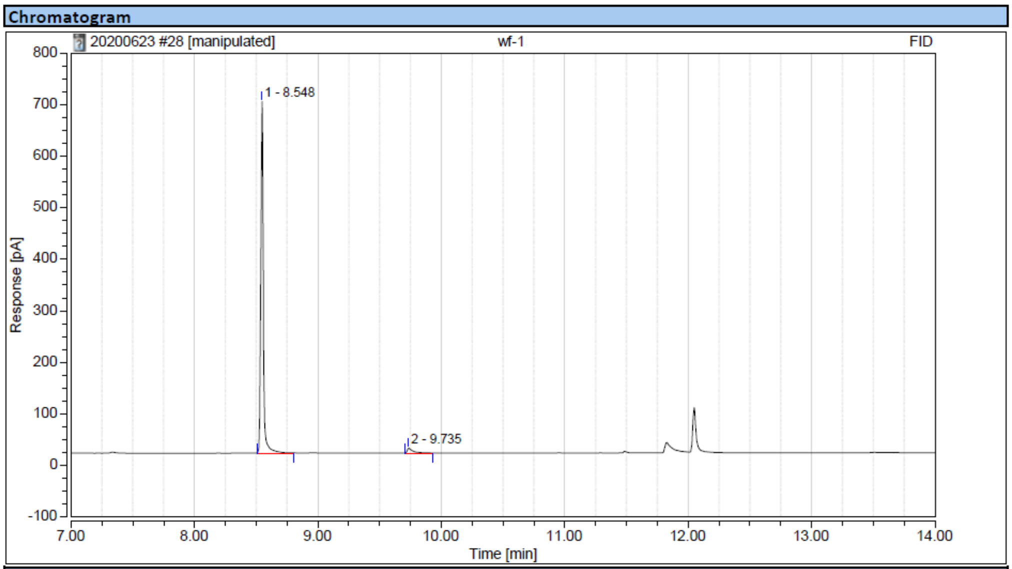

Integration Results

No.	Peak Name	Retention Time min	Area pA*min	Height pA	Relative Area %	Relative Height %	Amount n.a.
1		8.548	16.421	681.969	96.88	98.55	n.a.
2		9.735	0.529	10.018	3.12	1.45	n.a.
**Total:**			**16.950**	**691.987**	**100.00**	**100.00**	

Supplementary figure 2. (3-bromopropyl)benzene **1b** (39.6 mg, 0.2 mmol) and 1,4-di-*tert*-butyltetrasulfane **2a** (58.1 mg 0.24 mmol), $Ni(PPh_3)_2Cl_2$ (2.6 mg, 5.0 mol %), Ligand (10 mol %), Mn (1.5 equiv), DMF (1.0 mL). **24 h**, 40 ºC. After **24 h**, pure product **3b** and **3b' (31 : 1)** was otained.

Conclusion: The above experiments indicate that trisulfide 3b' may be a reaction intermediate.

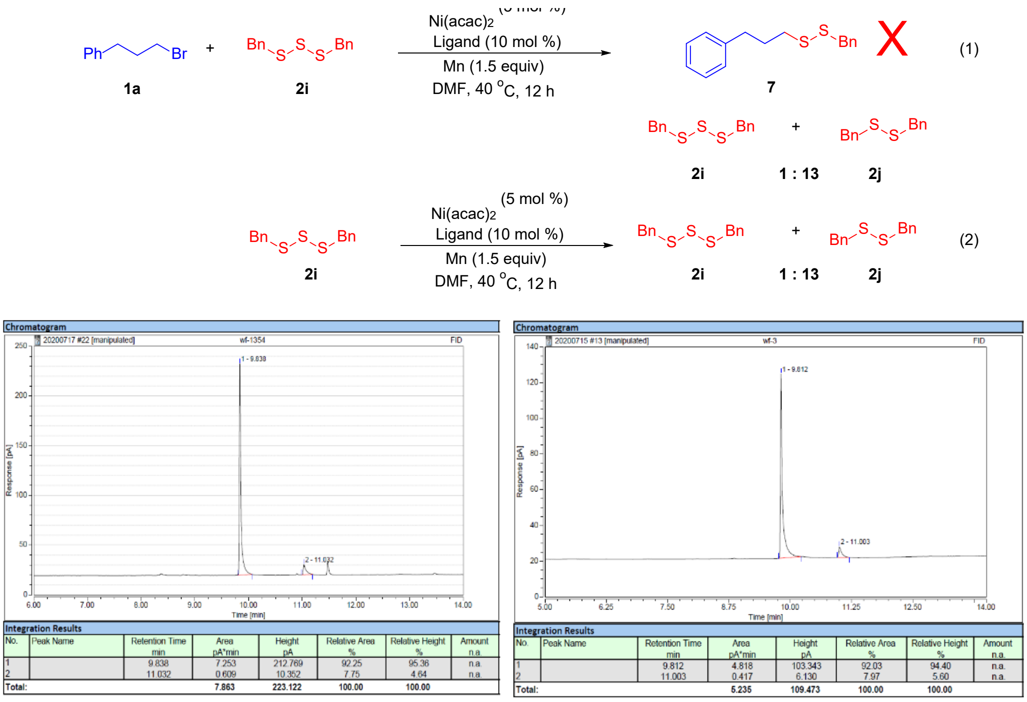

Supplementary figure 3. (1) (3-bromopropyl)benzene **1b** (0.2 mmol), 1,3-dibenzyltrisulfane **2i** (0.24 mmol), Ni(acac)$_2$ (5.0 mol %), Ligand (10 mol %), Mn (1.5 equiv), DMF (1.0 mL). 12 h, 40 ºC. The target product **7** could not be obtained instead of a mixture of product of **2i** and **2j** (1:13). (2) 1,3-dibenzyltrisulfane **2i** (0.24 mmol), Ni(acac)$_2$ (5.0 mol %), Ligand (10 mol %), Mn (1.5 equiv), DMF (1.0 mL). 12 h, 40 ºC. The mixture product of **2i** and **2j** could be obtained (1:13).

Conclusion: The above experiments show that under standard conditions, trisulfide is more likely to transform into disulfide by self-reaction.

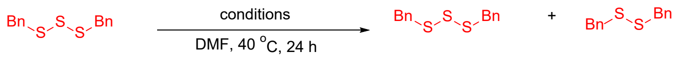

entry	conditions	6a : 7a
1	Ni(acac)$_2$ (5 mol %), Mn (1.5 equiv)	1 : 13
2	Ni(acac)$_2$ (5 mol %)	1 : 4.3
3	Mn (1.5 equiv)	1 : 3.8
4	-	1.8 : 1
5	Ni(COD)$_2$ (5 mol %)	1 : 1.2

Supplementary table 7. According to the experimental results, it was found that in this strategy, the complete conversion of trisulfide compounds to disulfide compounds, nickel catalyst and manganese both play a role.

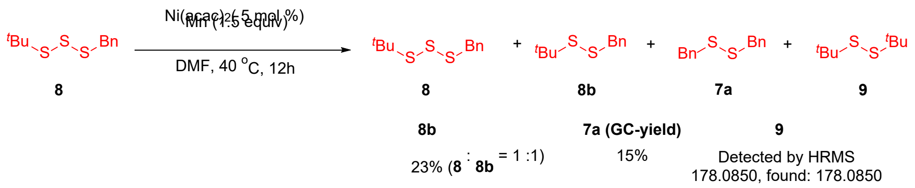

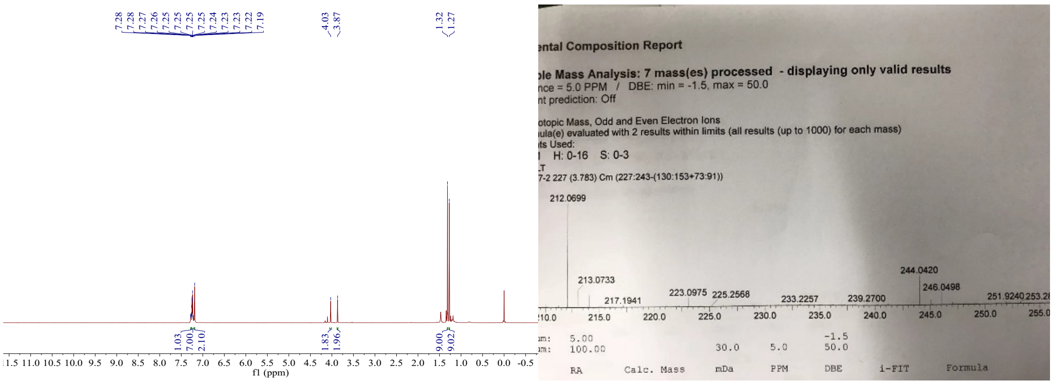

Supplementary figure 4. We investigated the conditions for the conversion of trisulfide **8** to disulfide **8b**. It was found that a mixture product of trisulfide **8** and disulfide **8b** could be obtained under standard conditions. The target product **8b** could be obtained in 23% yield. At the same time, we successfully isolated the symmetric benzyl disulfide product **7a** and successfully detected the *tert*-butyl disulfide product **9** by HRMS.

XPS spectra of S_8

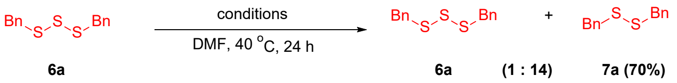

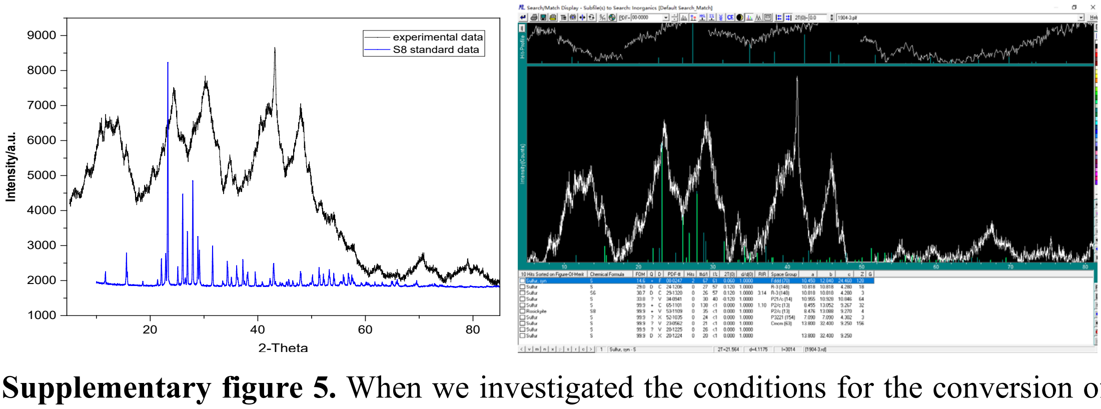

Supplementary figure 5. When we investigated the conditions for the conversion of trisulfide to disulfide. It was found that a large amount of precipitate was produced after the reaction. The black precipitate is poorly soluble in organic solvents. By X'Pert-Pro MPD (XRD) characterization, and Compared with the standard spectrum of S8, we could confirm the existence of S_8.

Product Characterization

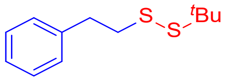

1-(*tert*-butyl)-2-phenethyldisulfane (3a)

Yield: 99% (44.8mg). Pale yellow oil. **IR** (neat, ν, cm^{-1}): 2959, 1496, 1453, 1361, 1164, 747, 697. **^{1}H NMR** (400 MHz, $CDCl_3$) δ 7.31 – 7.25 (m, 2H), 7.22 – 7.16 (m, 3H), 2.99 – 2.89 (m, 4H), 1.33 (s, 9H). **^{13}C NMR** (100 MHz, $CDCl_3$) δ 140.3, 128.7, 128.6, 126.4, 48.0, 42.0, 35.9, 30.1. **HRMS** (CI) m/z (M^+) calcd for $C_{12}H_{18}S_2$: 226.0850, found 226.0845.

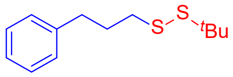

1-(*tert*-butyl)-2-(3-phenylpropyl)disulfane (3b)

Yield: 99% (47.5mg). Pale yellow oil. **IR** (neat, ν, cm^{-1}): 2958, 2858, 1496, 1454, 1361, 1164, 742, 698. **^{1}H NMR** (400 MHz, $CDCl_3$) δ 7.31 – 7.25 (m, 2H), 7.21 – 7.14 (m, 3H), 2.70 (td, *J* = 7.4, 5.4 Hz, 4H), 2.04 – 1.94 (m, 2H), 1.31 (s, 9H). **^{13}C NMR** (100 MHz, $CDCl_3$) δ 141.5, 128.6, 128.5, 126.0, 47.8, 40.0, 34.5, 30.8, 30.1. **HRMS** (ESI^+, MeCN) m/z calcd for $C_{13}H_{21}S_2$ $(M+H)^+$: 241.1085, found 241.1085.

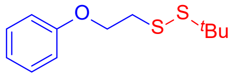

1-(*tert*-butyl)-2-(2-phenoxyethyl)disulfane (3c)

Yield: 82% (39.7mg). Pale yellow oil. **IR** (neat, ν, cm^{-1}): 2960, 1599, 1495, 1362, 1239, 1165, 1032, 750, 690. **^{1}H NMR** (400 MHz, $CDCl_3$) δ 7.29 – 7.25 (m, 2H), 6.92 (ddt, *J* = 8.7, 7.8, 1.0 Hz, 3H), 4.20 (t, *J* = 7.0 Hz, 2H), 3.04 (t, *J* = 7.0 Hz, 2H), 1.35 (s, 9H). **^{13}C NMR** (100 MHz, $CDCl_3$) δ 158.5, 129.6, 121.1, 114.7, 66.6, 48.1, 39.1, 30.0. **HRMS** (ESI^+, MeCN) m/z calcd for $C_{12}H_{18}ONaS_2$ $(M+Na)^+$: 265.0697, found 265.0689.

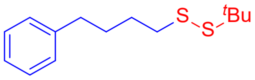

1-(*tert*-butyl)-2-(4-phenylbutyl)disulfane (3d)

Yield: 98% (49.8mg). Pale yellow oil. **IR** (neat, ν, cm^{-1}): 2934, 1454, 1361, 1165, 745, 697. **^{1}H NMR** (400 MHz, $CDCl_3$) δ 7.29 – 7.23 (m, 2H), 7.19 – 7.13 (m, 3H), 2.78 – 2.68 (m, 2H), 2.66 – 2.57 (m, 2H), 1.70 (p, *J* = 3.2 Hz, 4H), 1.31 (s, 9H). **^{13}C NMR** (100 MHz, $CDCl_3$) δ 142.2, 128.5, 128.4, 125.8, 47.7, 40.8, 35.6, 30.4, 30.1, 29.0. **HRMS** (CI) m/z (M^+) calcd for $C_{14}H_{22}S_2$: 254.1163, found 254.1161.

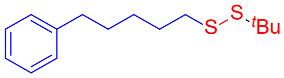

1-(*tert*-butyl)-2-(5-phenylpentyl)disulfane (3e)

Yield: 79% (42.4mg). Pale yellow oil. **IR** (neat, ν, cm^{-1}): 2928, 2856, 1454, 1361, 1165, 745, 697. **^{1}H NMR** (400 MHz, $CDCl_3$) δ 7.29 – 7.23 (m, 2H), 7.18 – 7.13 (m, 3H), 2.71 – 2.65 (m, 2H), 2.63 – 2.57 (m, 2H), 1.72 – 1.58 (m, 4H), 1.45 – 1.38 (m, 2H), 1.32 (s, 9H). **^{13}C NMR** (100 MHz, $CDCl_3$) δ 142.6, 128.5, 128.4, 125.8, 47.7, 40.9, 35.9, 31.2, 30.1, 29.3, 28.3. **HRMS** (CI) m/z (M^+) calcd for $C_{15}H_{24}S_2$: 268.1319, found 268.1317.

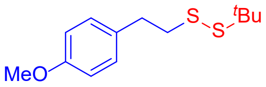

1-(*tert*-butyl)-2-(4-methoxyphenethyl)disulfane (3f)

Yield: 90% (46.1mg). Pale yellow oil. **IR** (neat, ν, cm^{-1}): 2903, 1510, 1455, 1175, 801, 754. **^{1}H NMR** (400 MHz, $CDCl_3$) δ 7.14 – 7.08 (m, 2H), 6.85 – 6.80 (m, 2H), 3.77 (s, 3H), 2.90 (s, 4H), 1.33 (s, 9H). **^{13}C NMR** (100 MHz, $CDCl_3$) δ 158.2, 132.4, 129.6, 114.0, 55.3, 47.9, 42.3, 34.9, 30.1. **HRMS** (CI) m/z (M^+) calcd for $C_{13}H_{20}OS_2$: 256.0956, found 256.0954.

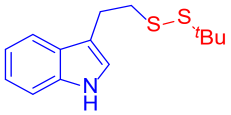

3-(2-(*tert*-butyldisulfanyl)ethyl)-1H-indole (3g)

Yield: 95% (50.4mg). Pale yellow oil. **IR** (neat, ν, cm^{-1}): 3412, 2958, 1720, 1455, 1360, 1163, 1091, 1010, 738, 581. **^{1}H NMR** (400 MHz, $CDCl_3$) δ 7.98 (s, 1H), 7.71 – 7.65 (m, 1H), 7.39 (dt, J = 8.1, 1.0 Hz, 1H), 7.29 – 7.24 (m, 1H), 7.20 (ddd, J = 8.0, 7.0, 1.2 Hz, 1H), 7.07 – 7.03 (m, 1H), 3.24 – 3.17 (m, 2H), 3.10 (ddd, J = 8.2, 6.7, 1.2 Hz, 2H), 1.43 (s, 9H). **^{13}C NMR** (100 MHz, $CDCl_3$) δ 136.3, 127.3, 122.1, 121.9, 119.4, 118.8, 114.6, 111.3, 47.9, 41.2, 30.1, 25.5. **HRMS** (ESI^+, MeCN) m/z calcd for $C_{14}H_{19}NNaS_2$ $(M+Na)^+$: 288.0857, found 288.0855.

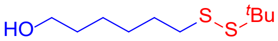

6-(*tert*-butyldisulfanyl)hexan-1-ol (3h)

Yield: 65% (28.9mg). Pale yellow oil. **IR** (neat, ν, cm^{-1}): 2928, 2857, 1455, 1361, 1165, 1053, 726. **^{1}H NMR** (400 MHz, $CDCl_3$) δ 3.63 (t, J = 6.6 Hz, 2H), 2.76 – 2.65 (m, 2H), 1.86 (s, 1H), 1.71 – 1.63 (m, 2H), 1.62 – 1.53 (m, 2H), 1.40 (tdd, J = 5.8, 3.6, 2.3 Hz, 4H), 1.33 (s, 9H). **^{13}C NMR** (100 MHz, $CDCl_3$) δ 62.9, 47.8, 40.9, 32.6, 30.0, 29.3, 28.4, 25.4. **HRMS** (CI) m/z (M^+) calcd for $C_{10}H_{22}OS_2$: 222.1112, found 222.1109.

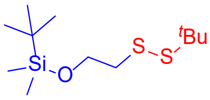

***tert*-butyl(2-(tert-butyldisulfanyl)ethoxy)dimethylsilane (3i)**

Yield: 83% (46.5mg). Pale yellow oil. **IR** (neat, ν, cm^{-1}): 2956, 2928, 2857, 1471, 1361, 1254, 1088, 834, 775. ^{1}**H NMR** (400 MHz, $CDCl_3$) δ 3.82 (t, *J* = 6.9 Hz, 2H), 2.81 (t, *J* = 6.9 Hz, 2H), 1.33 (s, 9H), 0.90 (s, 9H), 0.07 (s, 6H). ^{13}**C NMR** (100 MHz, $CDCl_3$) δ 62.4, 47.9, 43.1, 30.0, 26.1, 18.5, -5.1. **HRMS** (CI) m/z (M^+) calcd for $C_{12}H_{28}OSiS_2$: 280.1351, found 280.1358.

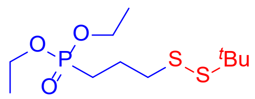

diethyl (3-(*tert*-butyldisulfanyl)propyl)phosphonate (3j)

Yield: 50% (30.0 mg). Pale yellow oil. **IR** (neat, ν, cm^{-1}): 2963, 1713, 1362, 1221, 1165, 1025, 957, 781, 529. ^{1}**H NMR** (400 MHz, $CDCl_3$) δ 4.16 – 4.05 (m, 4H), 2.80 – 2.72 (m, 2H), 2.05 – 1.92 (m, 2H), 1.91 – 1.78 (m, 2H), 1.37 – 1.27 (m, 15H). ^{13}**C NMR** (100 MHz, $CDCl_3$) δ 61.7(*J*=6.14 Hz), 47.9, 40.8, 40.6, 30.0, 25.0, 23.6, 22.2(*J*=5.3 Hz), 16.5(*J*=5.8 Hz). **HRMS** (CI) m/z (M^+) calcd for $C_{11}H_{25}O_3PS_2$: 300.0983, found 300.0987.

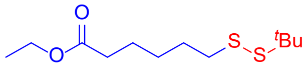

ethyl 6-(*tert*-butyldisulfanyl)hexanoate (3k)

Yield: 34% (18.0 mg). Pale yellow oil. **IR** (neat, ν, cm^{-1}): 2926, 2859, 1734, 1456, 1362, 1253, 1165, 1031. ^{1}**H NMR** (400 MHz, $CDCl_3$) δ 4.13 (q, *J* = 7.1 Hz, 2H), 2.70 (t, *J* = 7.3 Hz, 2H), 2.30 (t, *J* = 7.5 Hz, 2H), 1.72 – 1.61 (m, 4H), 1.46 – 1.38 (m, 2H), 1.33 (s, 9H), 1.26 (t, *J* = 7.1 Hz, 3H). ^{13}**C NMR** (100 MHz, $CDCl_3$) δ 173.7, 60.4, 47.8, 40.7, 34.3, 30.1, 29.0, 28.1, 24.7, 14.4. **HRMS** (ESI^+, MeCN) m/z calcd for $C_{12}H_{24}O_2S_2$ $(M+Na)^+$: 287.1115, found 287.1099.

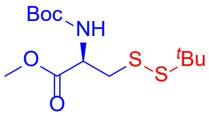

methyl *N*-(*tert*-butoxycarbonyl)-S-(*tert*-butylthio)-*L*-cysteinate (3l)

Yield: 82% (53.0 mg). Pale yellow oil. **IR** (neat, ν, cm^{-1}): 2968, 1746, 1714, 1499, 1363, 1159, 1050, 1015. ^{1}**H NMR** (400 MHz, $CDCl_3$) δ 5.44 (d, *J* = 8.1 Hz, 1H), 4.72 – 4.47 (m, 1H), 3.77 (s, 3H), 3.16 (t, *J* = 5.4 Hz, 1H), 1.45 (s, 9H), 1.33 (s, 9H). ^{13}**C NMR** (100 MHz, $CDCl_3$) δ 171.3, 155.1, 80.1, 53.2, 52.5, 48.1, 42.7, 29.8, 28.3. **HRMS** (ESI^+, MeCN) m/z calcd for $C_{13}H_{25}O_4NS_2$ $(M+Na)^+$: 346.1123, found 346.1129.

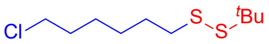

1-(*tert*-butyl)-2-(6-chlorohexyl)disulfane (3m)

Yield: 67% (32.2mg). Pale yellow oil. **IR** (neat, ν, cm^{-1}): 2956, 2931, 2857, 1455, 1361,

1165, 727, 651. **^{1}H NMR** (400 MHz, $CDCl_3$) δ 3.54 (td, *J* = 6.7, 2.0 Hz, 2H), 2.74 – 2.68 (m, 2H), 1.82 – 1.75 (m, 2H), 1.72 – 1.63 (m, 2H), 1.48 – 1.40 (m, 4H), 1.33 (s, 9H). **^{13}C NMR** (100 MHz, $CDCl_3$) δ 47.8, 45.1, 40.7, 32.5, 30.1, 29.2, 27.8, 26.6. **HRMS** (CI) m/z (M^+) calcd for $C_{10}H_{21}ClS_2$: 240.0773, found 240.0774.

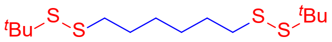

1,6-bis(*tert*-butyldisulfanyl)hexane (3m')

Yield: 20% (13.0mg). Pale yellow oil. **IR** (neat, ν, cm^{-1}): 2957, 2924, 2856, 1455, 1361, 1165, 726. **^{1}H NMR** (400 MHz, $CDCl_3$) δ 2.73 – 2.68 (m, 4H), 1.69 – 1.62 (m, 4H), 1.43 – 1.38 (m, 4H), 1.33 (s, 18H). **^{13}C NMR** (100 MHz, $CDCl_3$) δ 47.8, 40.9, 30.1, 29.3, 28.3. **HRMS** (CI) m/z (M^+) calcd for $C_{14}H_{30}S_4$: 326.1230, found 326.1228.

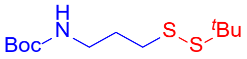

3n, 36% (6 : 1)

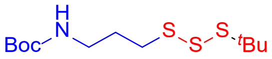

3n', 6%

***tert*-butyl (3-(*tert*-butyldisulfanyl)propyl)carbamate (3n), *tert*-butyl (3-(*tert*-butyltrisulfanyl)propyl)carbamate (3n').**

Yield: **3n** (36%), **3n'** (6%). Yellow oil. **IR** (neat, ν, cm^{-1}): 2964, 1687, 1511, 1363, 1347, 1162, 985. **^{1}H NMR** (400 MHz, $CDCl_3$) δ 4.68 (s, 1H), 4.50 (s, 0H), 3.21 (d, *J* = 6.5 Hz, 2H), 2.90 (t, *J* = 7.1 Hz, 0H), 2.72 (t, *J* = 7.2 Hz, 2H), 1.97 (dd, *J* = 13.7, 6.9 Hz, 0H), 1.86 (p, *J* = 7.0 Hz, 2H), 1.44 (s, 11H), 1.39 (s, 1H), 1.33 (s, 9H). **^{13}C NMR** (100 MHz, $CDCl_3$) δ 156.1, 79.3, 49.1, 48.0, 39.4, 37.8, 30.2, 30.1, 29.7, 28.5.
HRMS (ESI^+, MeCN) m/z calcd for $C_{15}H_{25}NO_2S_2$ $(M+Na)^+$: 302.1224, found 302.1213.
HRMS (ESI^+, MeCN) m/z calcd for $C_{15}H_{25}NO_2S_3$ $(M+Na)^+$: 334.0945, found 334.0938.

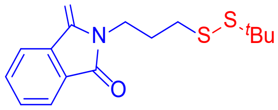

3o, 73% (9 : 1)

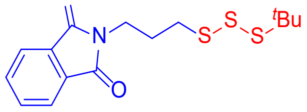

3o', 8%

2-(3-(*tert*-butyldisulfanyl)propyl)isoindoline-1,3-dione (3o), 2-(3-(tert-butyltrisulfanyl)propyl)isoindoline-1,3-dione (3o').

Yield: **3o** (73%), **3o'** (8%). Pale yellow oil. **IR** (neat, ν, cm^{-1}): 1774, 1709, 1397, 1363, 1166, 1008, 713, 529. **^{1}H NMR** (400 MHz, $CDCl_3$) δ 7.84 (dd, *J* = 5.4, 3.1 Hz, 2H), 7.72 (dd, *J* = 5.4, 3.1 Hz, 2H), 3.83 (d, *J* = 6.8 Hz, 0H), 3.78 (t, *J* = 7.0 Hz, 2H), 2.91 (t, *J* = 7.2 Hz, 0H), 2.76 – 2.69 (m, 2H), 2.20 – 2.15 (m, 0H), 2.07 (p, *J* = 7.1 Hz, 2H), 1.37 (s, 1H), 1.31 (s, 9H). **^{13}C NMR** (100 MHz, $CDCl_3$) δ 168.3, 134.0, 132.1, 123.3, 49.0, 47.8, 37.7, 36.9, 36.8, 36.3, 30.0, 29.9, 28.3, 27.8.
HRMS (ESI^+, MeCN) m/z calcd for $C_{15}H_{19}NO_2S_2$ $(M+Na)^+$: 333.0833, found 333.0800.
HRMS (ESI^+, MeCN) m/z calcd for $C_{15}H_{19}NO_2S_3$ $(M+Na)^+$: 364.0476, found 364.0474.

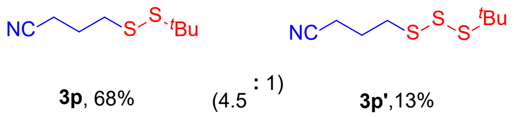

4-(*tert*-butyldisulfanyl)butanenitrile (3p), 4-(*tert*-butyltrisulfanyl)butanenitrile (3p')

Yield: **3p** (68%), **3p'** (13%). Pale yellow oil. **IR** (neat, ν, cm^{-1}): 2961, 2247, 1736, 1455, 1362, 1242, 1164, 1046. **^{1}H NMR** (400 MHz, $CDCl_3$) δ 2.99 (t, *J* = 6.7 Hz, 0H), 2.79 (t, *J* = 6.8 Hz, 2H), 2.57 (d, *J* = 7.1 Hz, 1H), 2.51 (t, *J* = 7.1 Hz, 2H), 2.20 – 2.15 (m, 1H), 2.06 (p, *J* = 6.9 Hz, 2H), 1.39 (s, 2H), 1.34 (s, 9H). **^{13}C NMR** (100 MHz, $CDCl_3$) δ 119.1, 119.1, 49.3, 48.2, 38.1, 37.1, 30.1, 30.0, 29.9, 24.8, 24.2, 15.8, 15.8.
HRMS (CI) m/z (M^+) calcd for $C_8H_{15}NS_2$: 189.0646, found 189.0641.
HRMS (CI) m/z (M^+) calcd for $C_8H_{15}NS_3$: 221.0367, found 221.0372.

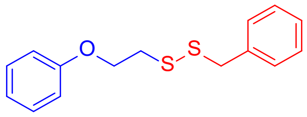

1-benzyl-2-(2-phenoxyethyl)disulfane (4a)

Yield: 68% (37.5mg). Pale yellow oil. **IR** (neat, ν, cm^{-1}): 1599, 1584, 1493, 1453, 1238, 1171, 1013, 752, 690. **^{1}H NMR** (400 MHz, $CDCl_3$) δ 7.40 – 7.36 (m, 4H), 7.36 – 7.32 (m, 3H), 7.02 (m, *J* = 7.4, 1.1 Hz, 1H), 6.96 – 6.89 (m, 2H), 4.14 (t, *J* = 6.7 Hz, 2H), 3.97 (s, 2H), 2.82 (t, *J* = 6.7 Hz, 2H). **^{13}C NMR** (100 MHz, $CDCl_3$) δ 158.4, 137.3, 129.6, 129.4, 128.7, 127.6, 121.1, 114.7, 66.1, 43.8, 37.4. **HRMS** (ESI^+, MeCN) m/z calcd for $C_{15}H_{16}ONaS_2$ $(M+Na)^+$: 299.0540, found 299.0534.

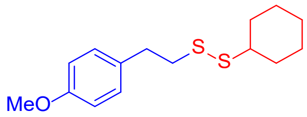

1-cyclohexyl-2-(4-methoxyphenethyl)disulfane (4b)

Yield: 91% (51.3mg). Pale yellow oil. **IR** (neat, ν, cm^{-1}): 2926, 2850, 1611, 1510, 1445, 1243, 1176, 1035, 818, 518. **^{1}H NMR** (400 MHz, $CDCl_3$) δ 7.14 – 7.07 (m, 2H), 6.86 – 6.79 (m, 2H), 3.77 (s, 3H), 2.94 – 2.84 (m, 4H), 2.75 – 2.66 (m, 1H), 2.06 – 1.95 (m, 2H), 1.77 (dt, *J* = 12.2, 3.6 Hz, 2H), 1.64 – 1.57 (m, 1H), 1.39 – 1.19 (m, 5H). **^{13}C NMR** (100 MHz, $CDCl_3$) δ 158.2, 132.3, 129.6, 113.9, 55.3, 49.7, 41.7, 34.9, 33.0, 26.2, 25.7. **HRMS** (CI) m/z (M^+) calcd for $C_{15}H_{22}OS_2$: 282.1112, found 282.1112.

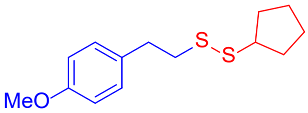

1-cyclopentyl-2-(4-methoxyphenethyl)disulfane (4c)

Yield: 84% (45.0mg). Pale yellow oil. **IR** (neat, ν, cm^{-1}): 2952, 1611, 1510, 1441, 1243, 1176, 1035, 819, 518. **^{1}H NMR** (400 MHz, $CDCl_3$) δ 7.15 – 7.09 (m, 2H), 6.86 – 6.81

(m, 2H), 3.78 (s, 3H), 3.28 (tt, J = 7.1, 5.6 Hz, 1H), 2.91 (d, J = 2.3 Hz, 4H), 2.04 – 1.89 (m, 2H), 1.79 – 1.55 (m, 6H). **^{13}C NMR** (100 MHz, $CDCl_3$) δ 158.2, 132.4, 129.7, 114.0, 55.4, 50.4, 41.0, 35.0, 33.2, 24.8. **HRMS** (CI) m/z (M^+) calcd for $C_{14}H_{20}OS_2$: 268.0956, found 268.0955.

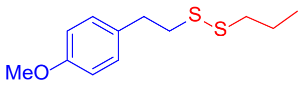

1-(4-methoxyphenethyl)-2-propyldisulfane (4d)

Yield: 67% (32.4mg). Pale yellow oil. **IR** (neat, ν, cm^{-1}): 2958, 1611, 1510, 1461, 1243, 1176, 1035, 819, 518. **^{1}H NMR** (400 MHz, $CDCl_3$) δ 7.15 – 7.09 (m, 2H), 6.87 – 6.80 (m, 2H), 3.79 (s, 3H), 2.97 – 2.84 (m, 4H), 2.71 – 2.63 (m, 2H), 1.71 (h, J = 7.3 Hz, 2H), 1.00 (t, J = 7.3 Hz, 3H). **^{13}C NMR** (100 MHz, $CDCl_3$) δ 158.3, 132.3, 129.7, 114.0, 55.4, 41.3, 40.7, 35.0, 22.7, 13.3. **HRMS** (ESI^+, MeCN) m/z calcd for $C_{12}H_{18}ONaS_2$ $(M+Na)^+$: 265.0697, found 265.0694.

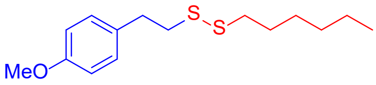

1-hexyl-2-(4-methoxyphenethyl)disulfane (4e)

Yield: 65% (36.9mg). Pale yellow oil. **IR** (neat, ν, cm^{-1}): 2925, 1611, 1511, 1463, 1244, 1176, 1036, 819, 519. **^{1}H NMR** (400 MHz, $CDCl_3$) δ 7.15 – 7.08 (m, 2H), 6.87 – 6.80 (m, 2H), 3.78 (s, 3H), 2.96 – 2.85 (m, 4H), 2.72 – 2.66 (m, 2H), 1.74 – 1.62 (m, 2H), 1.44 – 1.35 (m, 2H), 1.33 – 1.25 (m, 4H), 0.93 – 0.83 (m, 3H). **^{13}C NMR** (100 MHz, $CDCl_3$) δ 158.3, 132.4, 129.7, 114.0, 55.4, 40.7, 39.3, 35.0, 31.6, 29.4, 28.3, 22.7, 14.2. **HRMS** (ESI^+, MeCN) m/z calcd for $C_{15}H_{24}OS_2$ $(M+Na)^+$: 307.1166, found 307.1172.

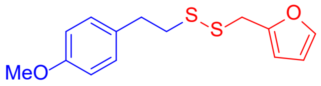

2-(((4-methoxyphenethyl)disulfanyl)methyl)furan (4f)

Yield: 56% (31.4mg). Black oil. **IR** (neat, ν, cm^{-1}): 2922, 1610, 1510, 1243, 1176, 1033, 1009, 934, 736, 597. **^{1}H NMR** (400 MHz, $CDCl_3$) δ 7.38 (m, J = 1.9, 0.9 Hz, 1H), 7.08 – 7.00 (m, 2H), 6.85 – 6.78 (m, 2H), 6.33 (dd, J = 3.2, 1.9 Hz, 1H), 6.26 (dd, J = 3.3, 0.8 Hz, 1H), 3.90 (s, 2H), 3.78 (s, 3H), 2.82 (dd, J = 9.2, 6.4 Hz, 2H), 2.67 – 2.58 (m, 2H). **^{13}C NMR** (100 MHz, $CDCl_3$) δ 158.3, 150.6, 142.6, 132.2, 129.7, 114.0, 111.0, 109.0, 55.4, 40.2, 36.2, 34.6. **HRMS** (CI) m/z (M^+) calcd for $C_{14}H_{16}O_2S_2$: 280.0592, found 280.0592.

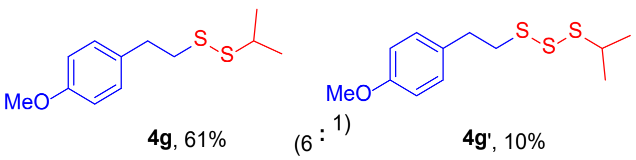

4g, 61% (6 : 1) **4g'**, 10%

1-isopropyl-2-(4-methoxyphenethyl)disulfane (4g), 1-isopropyl-3-(4-methoxyphenethyl)trisulfane(4g')

Yield: **4g** (61%), **4g'** (10%). Pale yellow oil. **IR** (neat, ν, cm^{-1}): 2957, 1611, 1510, 1440, 1243, 1176, 1035, 819, 518. **^{1}H NMR** (400 MHz, $CDCl_3$) δ 7.16 – 7.10 (m, 2H), 6.86 – 6.81 (m, 2H), 3.78 (d, J = 1.3 Hz, 3H), 3.24 – 3.18 (m, 0H), 3.11 – 3.08 (m, 0H), 3.04 – 2.97 (m, 1H), 2.96 – 2.86 (m, 4H), 1.37 (d, J = 6.7 Hz, 1H), 1.31 (d, J = 6.8 Hz, 6H). **^{13}C NMR** (100 MHz, $CDCl_3$) δ 158.3, 158.3, 132.4, 129.8, 129.7, 114.0, 55.4, 41.9, 41.6, 41.3, 40.5, 35.0, 34.5, 22.8, 22.6.

HRMS (CI) m/z (M^+) calcd for $C_{12}H_{18}OS_2$: 242.0799, found 242.0807.

HRMS (CI) m/z (M^+) calcd for $C_{12}H_{18}OS_3$: 274.0520, found 274.0520.

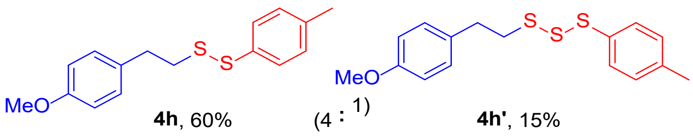

4h, 60% (4 : 1) **4h'**, 15%

1-(4-methoxyphenethyl)-2-(p-tolyl)disulfane (4h), 1-(4-methoxyphenethyl)-3-(p-tolyl)trisulfane (4h')

Yield: **4h** (60%), **4h'** (15%). Pale yellow oil. **IR** (neat, ν, cm^{-1}): 1610, 1510, 1488, 1243, 1176, 1034, 803, 486. **^{1}H NMR** (400 MHz, $CDCl_3$) δ 7.53 – 7.49 (m, 1H), 7.42 (d, J = 8.0 Hz, 2H), 7.13 (dd, J = 12.7, 7.9 Hz, 3H), 7.07 – 7.03 (m, 2H), 7.00 (d, J = 8.5 Hz, 1H), 6.82 – 6.78 (m, 3H), 3.76 (d, J = 2.0 Hz, 4H), 3.05 – 2.89 (m, 6H), 2.34 (s, 1H), 2.32 (s, 3H). **^{13}C NMR** (100 MHz, $CDCl_3$) δ 158.3, 158.3, 138.8, 137.2, 134.0, 133.8, 132.1, 131.9, 131.3, 130.8, 130.1, 130.1, 129.9, 129.7, 128.7, 114.0, 113.9, 55.3, 40.3, 40.2, 34.6, 34.4, 21.3, 21.2.

HRMS (CI) m/z (M^+) calcd for $C_{16}H_{18}OS_2$: 290.0799, found 290.0798.

HRMS (CI) m/z (M^+) calcd for $C_{16}H_{18}OS_3$: 322.0520, found 322.0525.

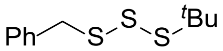

Prepared according to literature[3]. 1-benzyl-3-(tert-butyl)trisulfane (**8**). **^{1}H NMR** (400 MHz, $CDCl_3$) δ 7.25 (m, J = 4.5 Hz, 4H), 7.23 – 7.19 (m, 1H), 4.02 (s, 2H), 1.31 (s, 9H). **HRMS** (CI) m/z (M^+) calcd for $C_{11}H_{16}S_3$: 244.0414, found 244.0420. The values of the ^{1}H NMR spectrum are accordance with reported literature data. [3]

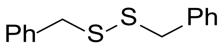

1,2-dibenzyldisulfane (**7a**). ^{1}H NMR (400 MHz, $CDCl_3$) δ 7.23 (m, J = 8.5, 6.7, 1.8 Hz, 4H), 7.20 – 7.15 (m, 6H), 3.53 (s, 4H). **HRMS** (CI) m/z (M^+) calcd for $C_{14}H_{14}S_2$: 246.0537, found 246.0539. The values of the ^{1}H NMR spectrum are accordance with reported literature data.[4]

NMR Spectroscopic Data

^{1}H NMR Spectra of **3a** (400 MHz, room temperature, $CDCl_3$).

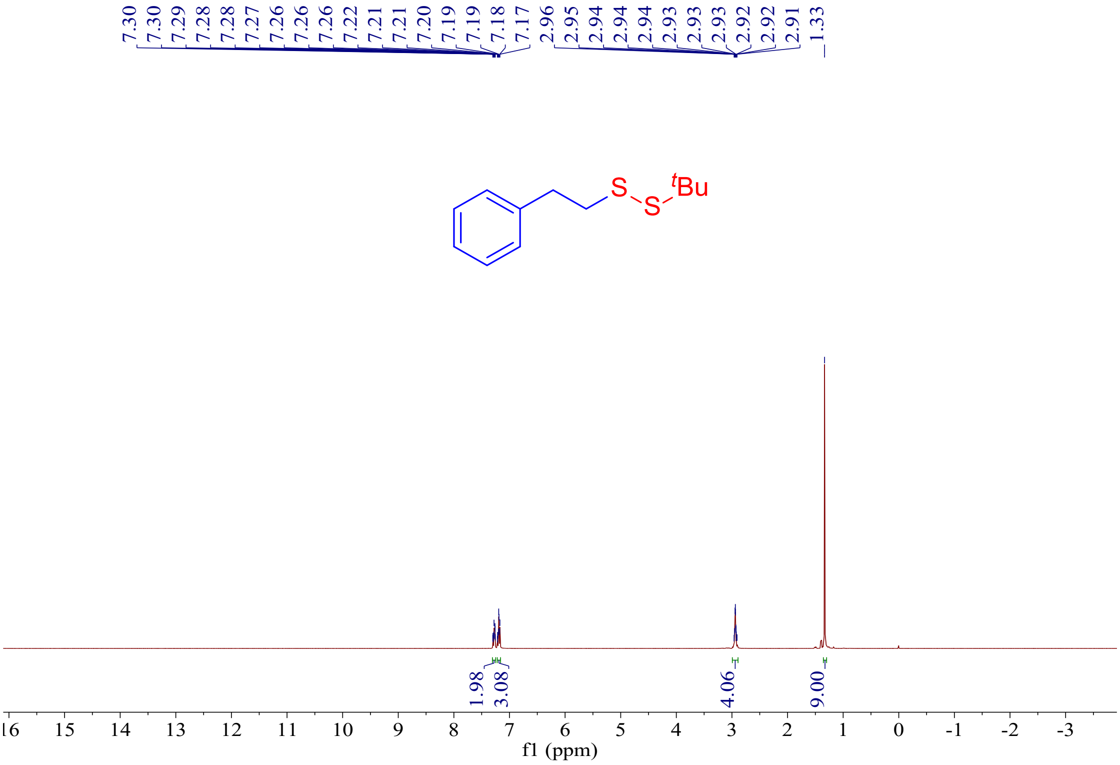

Supplementary Figure 6. ^{1}H NMR spectrum of **3a**.

^{13}C NMR Spectra of **3a** (100 MHz, room temperature, $CDCl_3$)

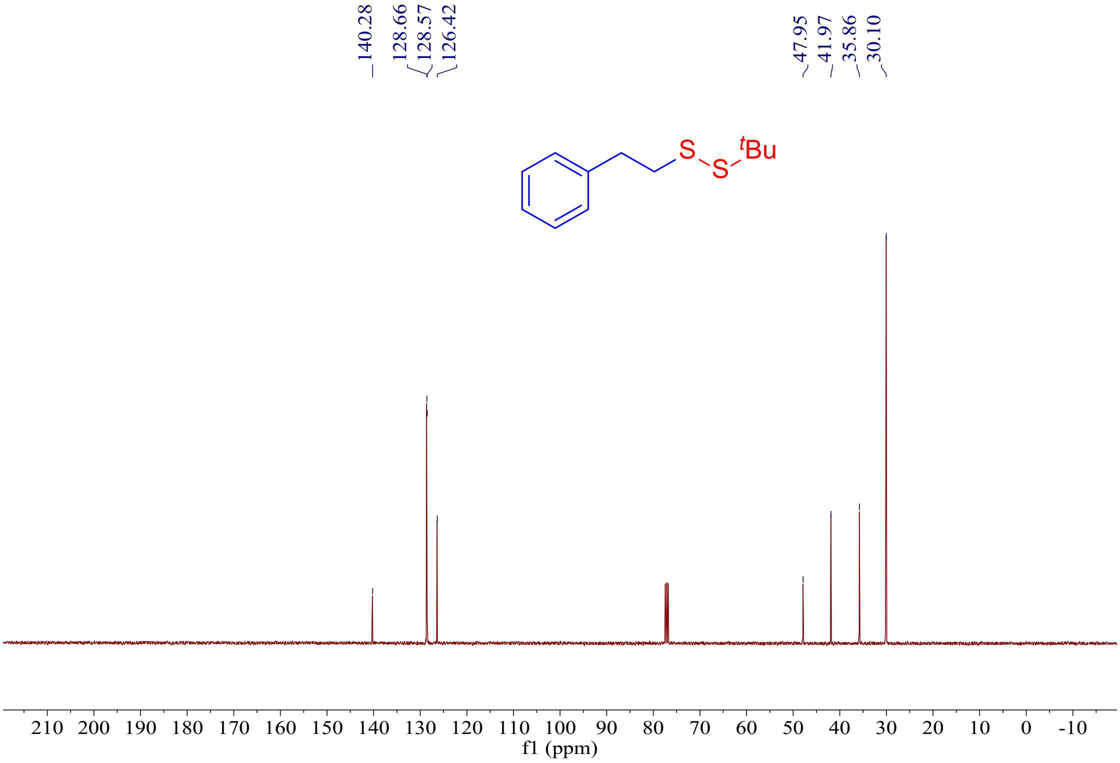

Supplementary Figure 7. ^{13}C NMR spectrum of **3a**.

^{1}H NMR Spectra of **3b** (400 MHz, room temperature, $CDCl_3$)

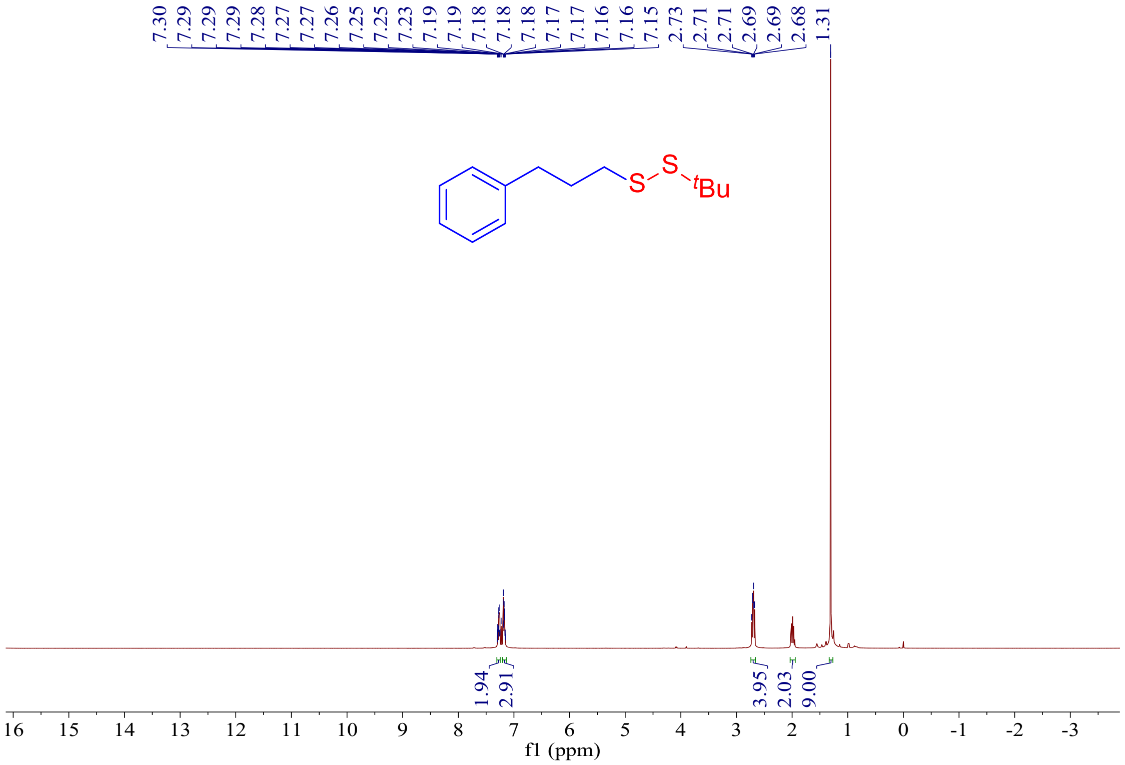

Supplementary Figure 8. ^{1}H NMR spectrum of **3b**.

^{13}C NMR Spectra of **3b** (100 MHz, room temperature, $CDCl_3$)

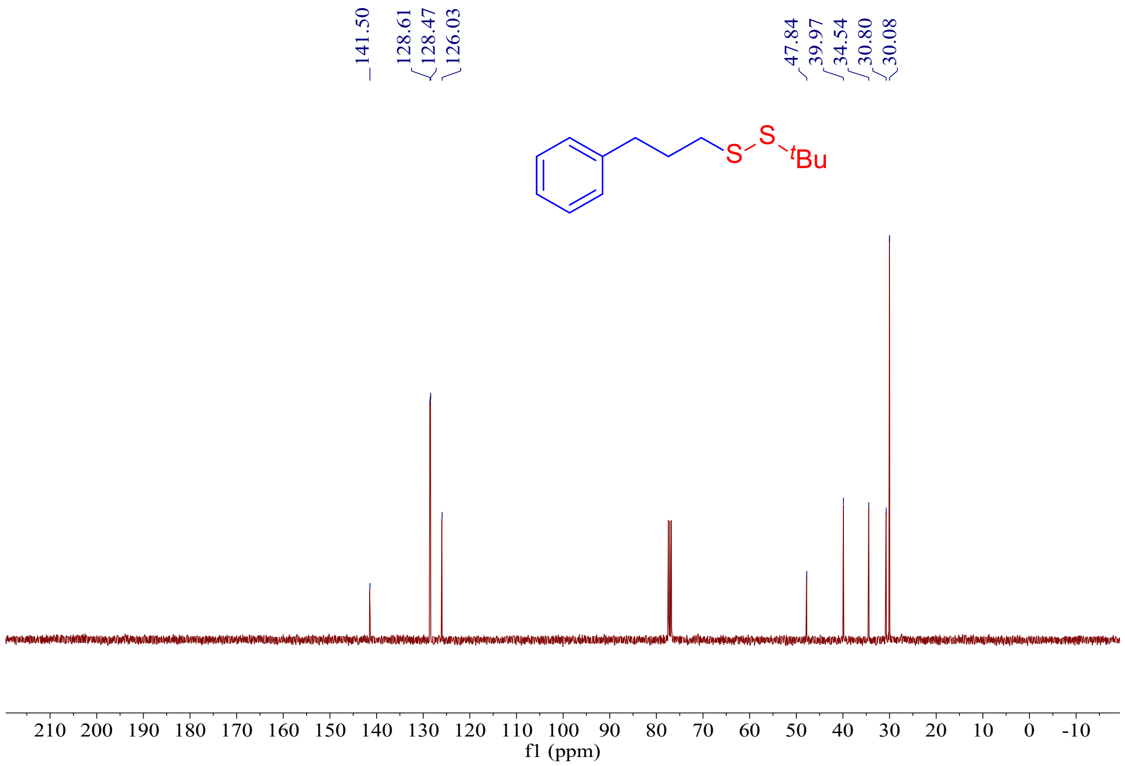

Supplementary Figure 9. ^{13}C NMR spectrum of **3b**.

^{1}H NMR Spectra of **3c** (400 MHz, room temperature, $CDCl_3$)

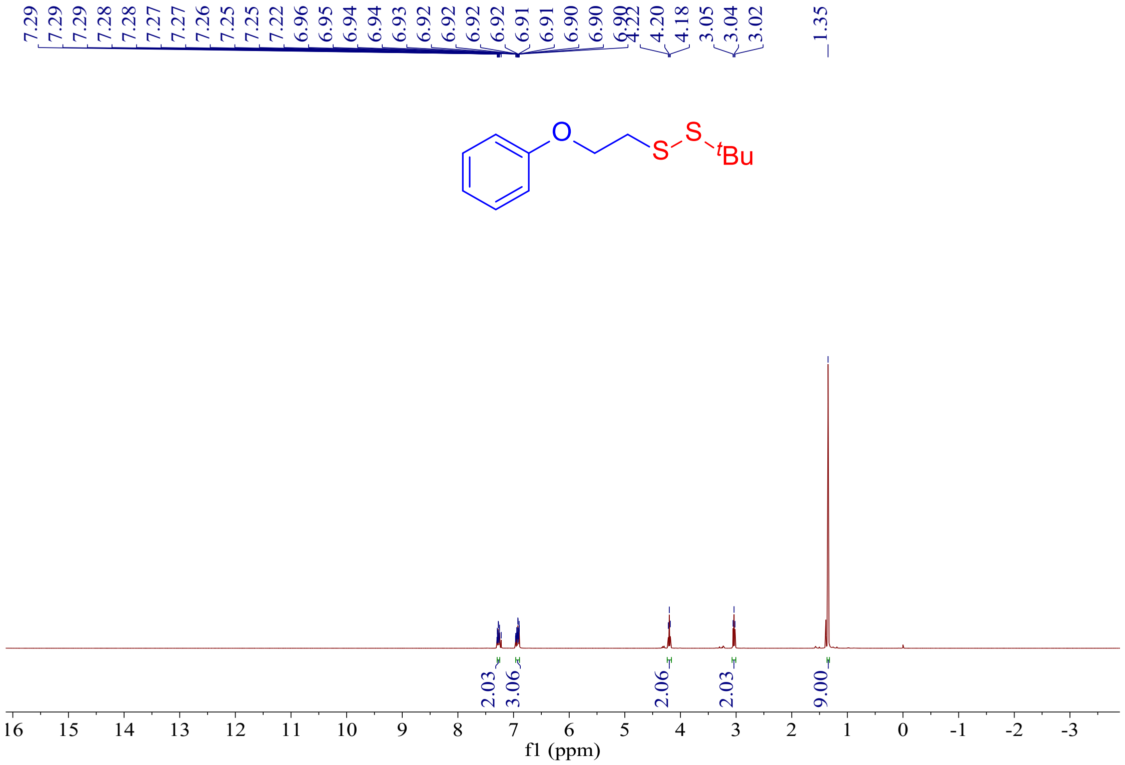

Supplementary Figure 10. ^{1}H NMR spectrum of **3c**.

^{13}C NMR Spectra of **3c** (100 MHz, room temperature, $CDCl_3$)

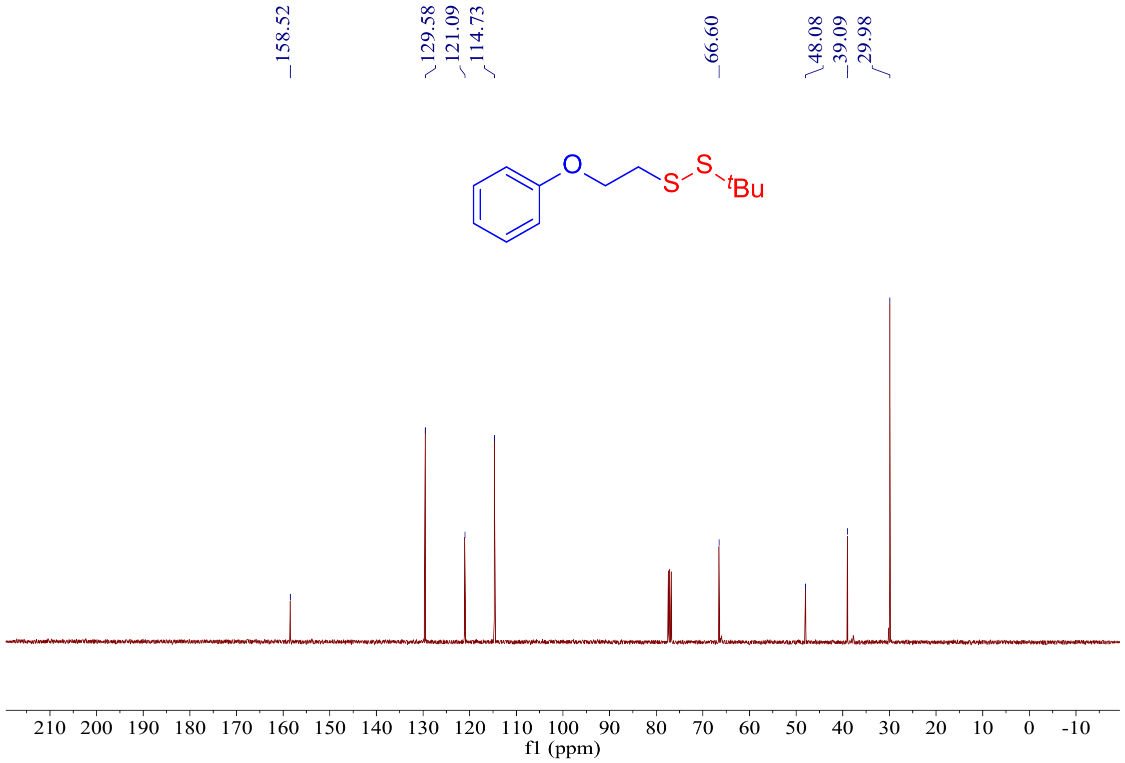

Supplementary Figure 11. ^{13}C NMR spectrum of **3c**.

^{1}H NMR Spectra of **3d** (400 MHz, room temperature, $CDCl_3$)

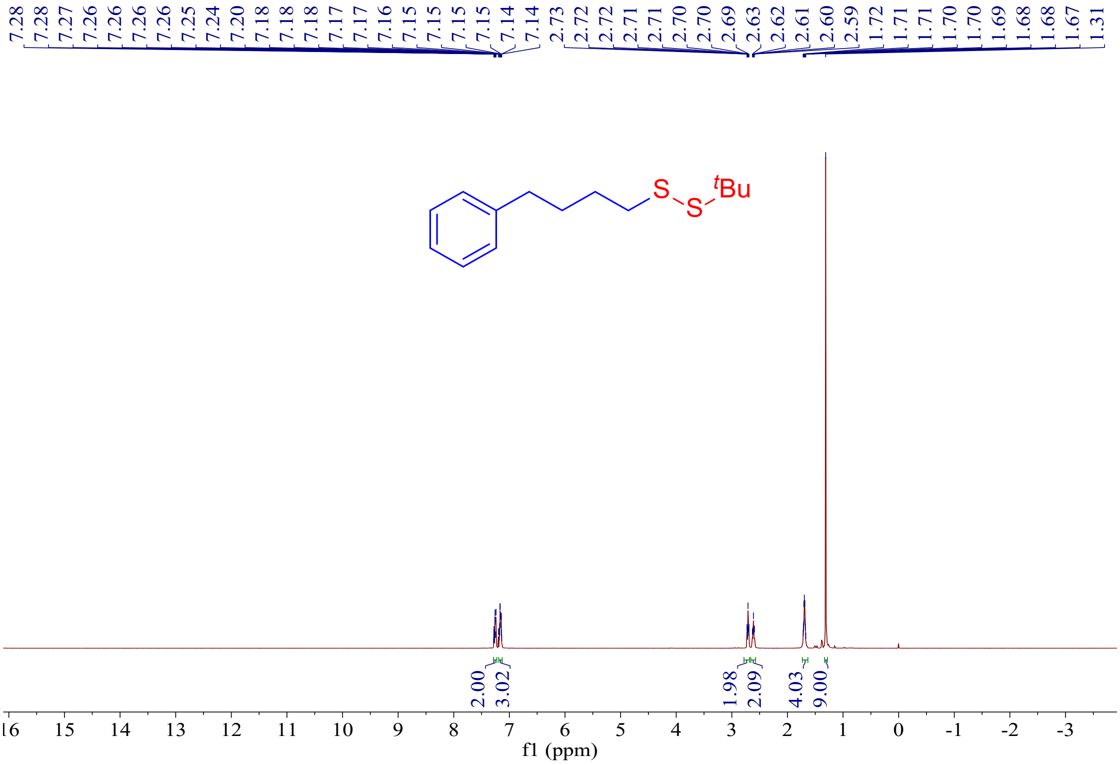

Supplementary Figure 12. ^{1}H NMR spectrum of **3d**.

^{13}C NMR Spectra of **3d** (100 MHz, room temperature, $CDCl_3$)

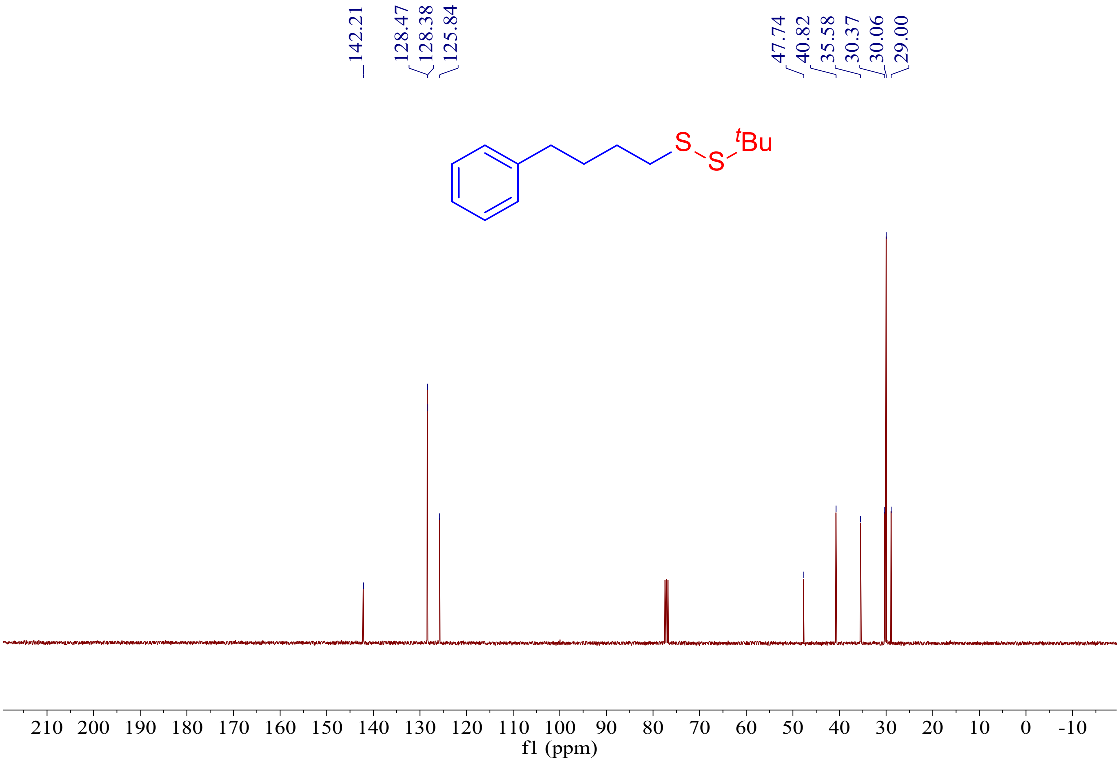

Supplementary Figure 13. ^{13}C NMR spectrum of **3d**.

^{1}H NMR Spectra of **3e** (400 MHz, room temperature, $CDCl_3$)

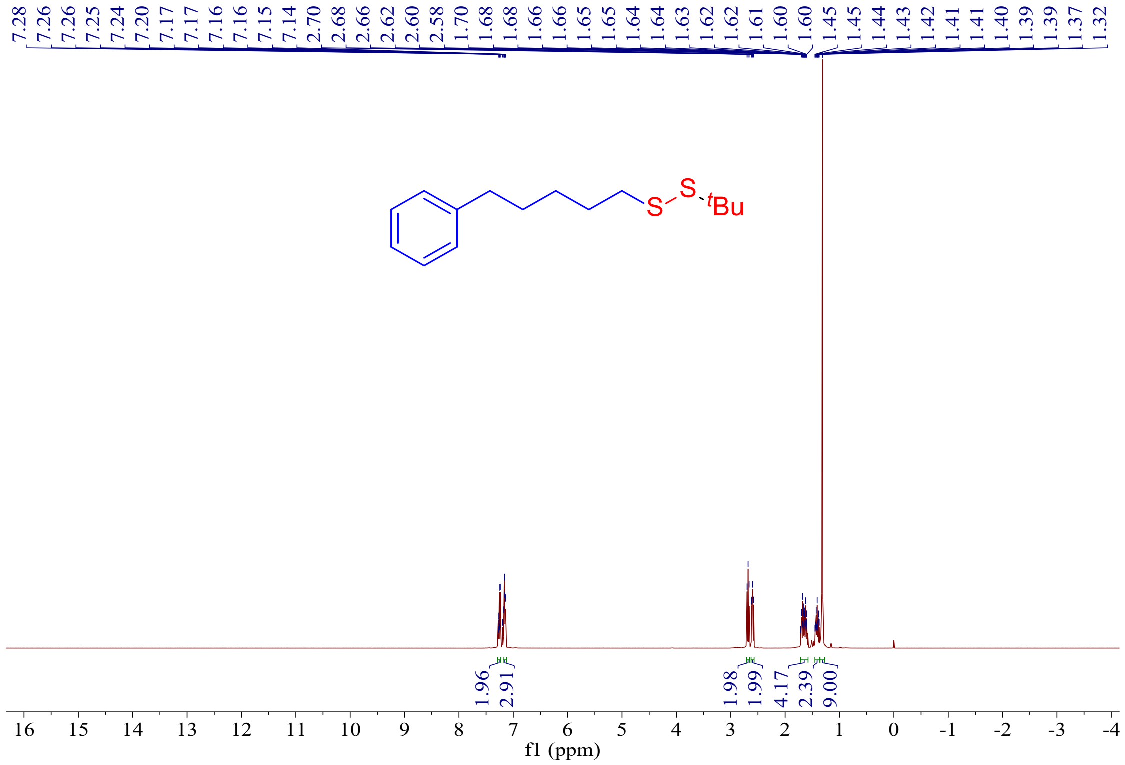

Supplementary Figure 14. ^{1}H NMR spectrum of **3e**.

^{13}C NMR Spectra of **3e** (100 MHz, room temperature, $CDCl_3$)

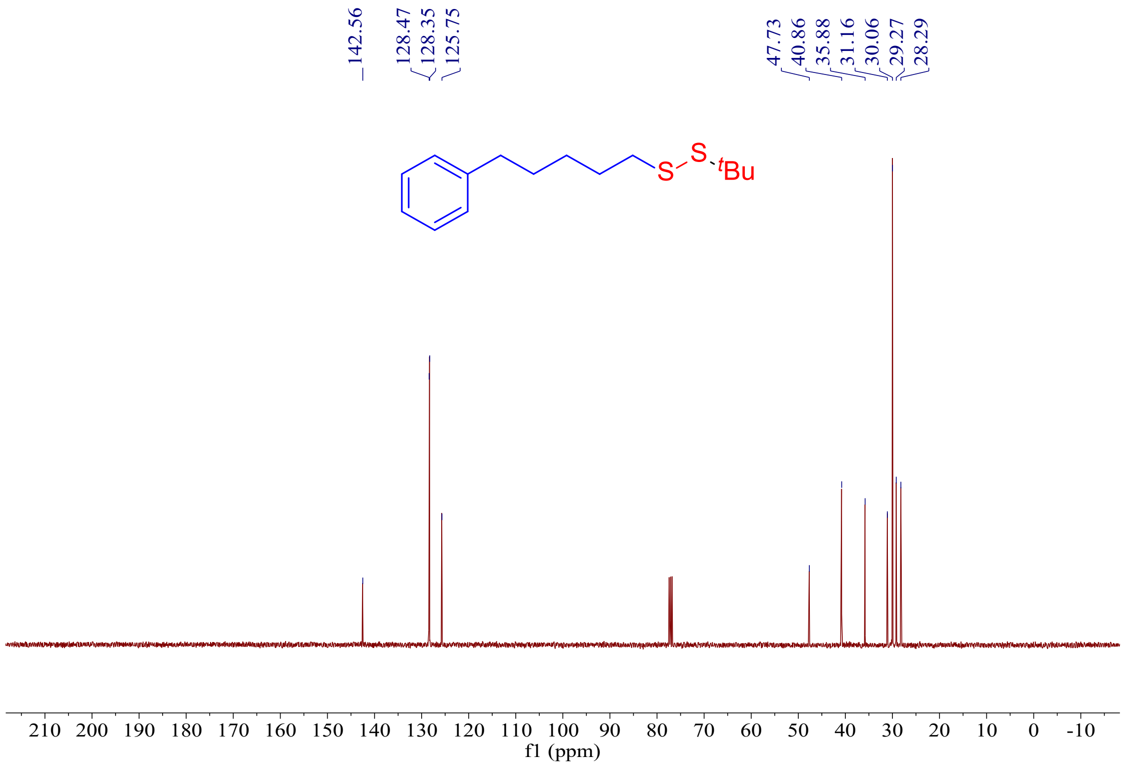

Supplementary Figure 15. ^{13}C NMR spectrum of **3e**.

^{1}H NMR Spectra of **3f** (400 MHz, room temperature, $CDCl_3$)

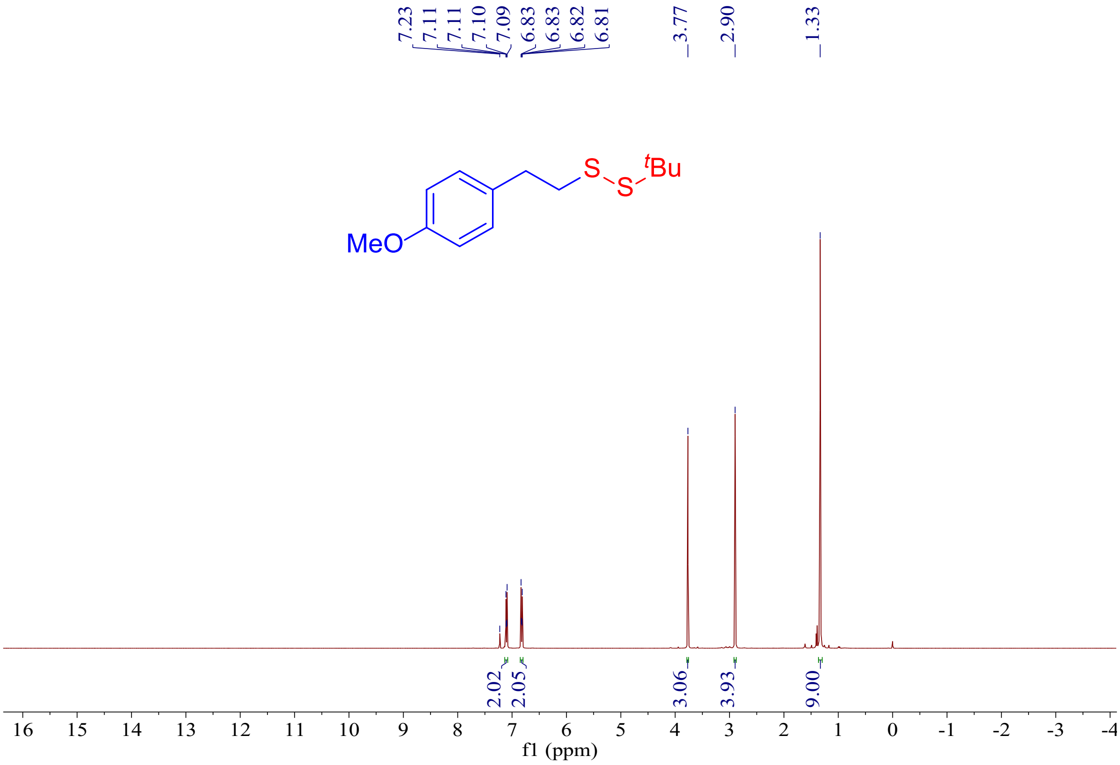

Supplementary Figure 16. ^{1}H NMR spectrum of **3f**.

^{13}C NMR Spectra of **3f** (100 MHz, room temperature, $CDCl_3$)

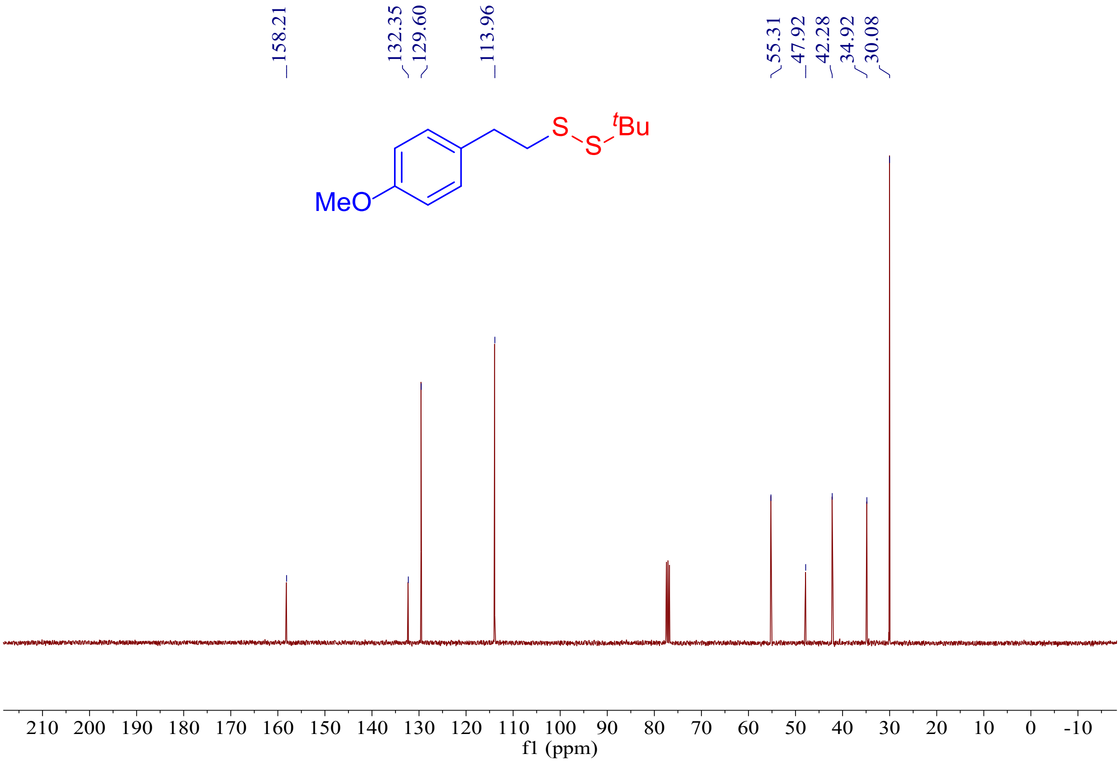

Supplementary Figure 17. ^{13}C NMR spectrum of **3f**.

^{1}H NMR Spectra of **3g** (400 MHz, room temperature, $CDCl_3$)

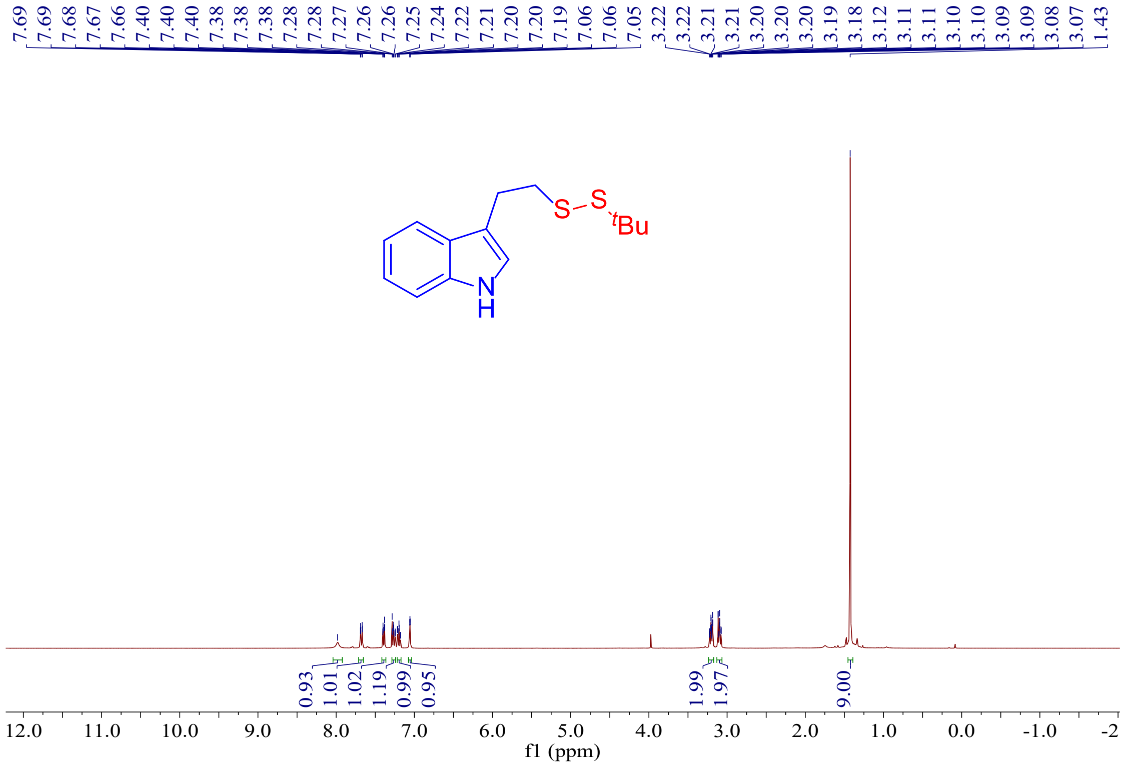

Supplementary Figure 18. ^{1}H NMR spectrum of **3g**.

^{13}C NMR Spectra of **3g** (100 MHz, room temperature, $CDCl_3$)

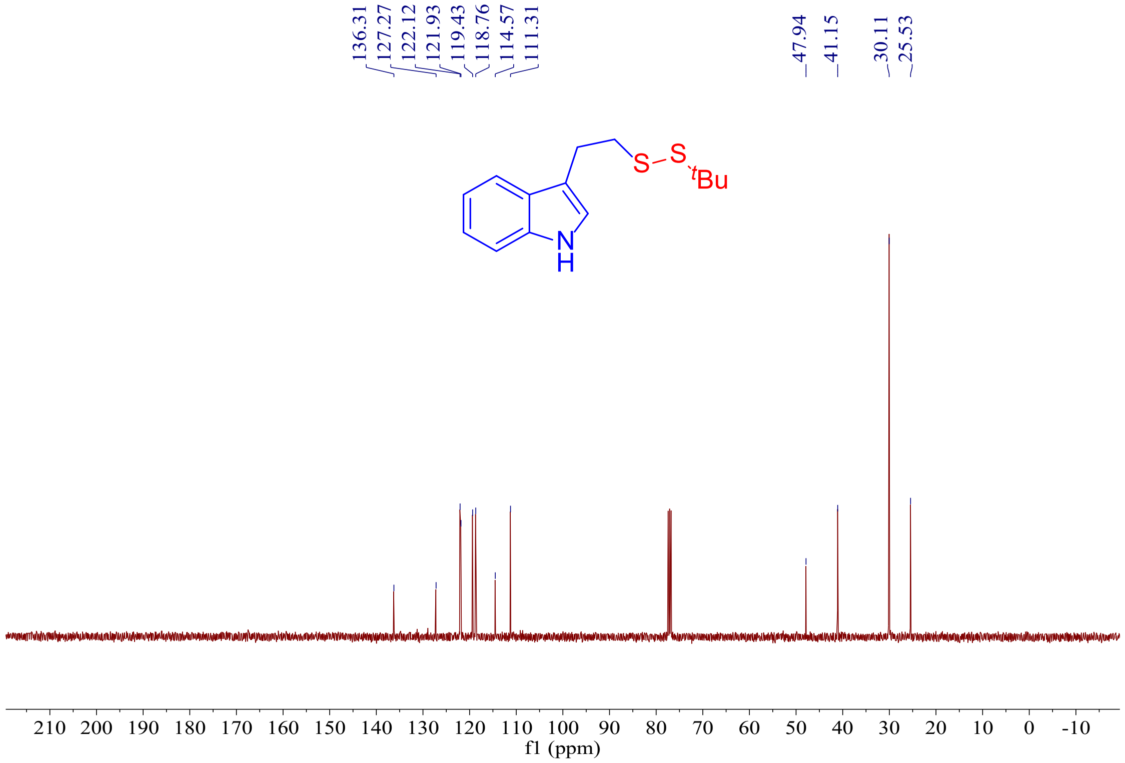

Supplementary Figure 19. ^{13}C NMR spectrum of **3g**.

^{1}H NMR Spectra of **3h** (400 MHz, room temperature, $CDCl_3$)

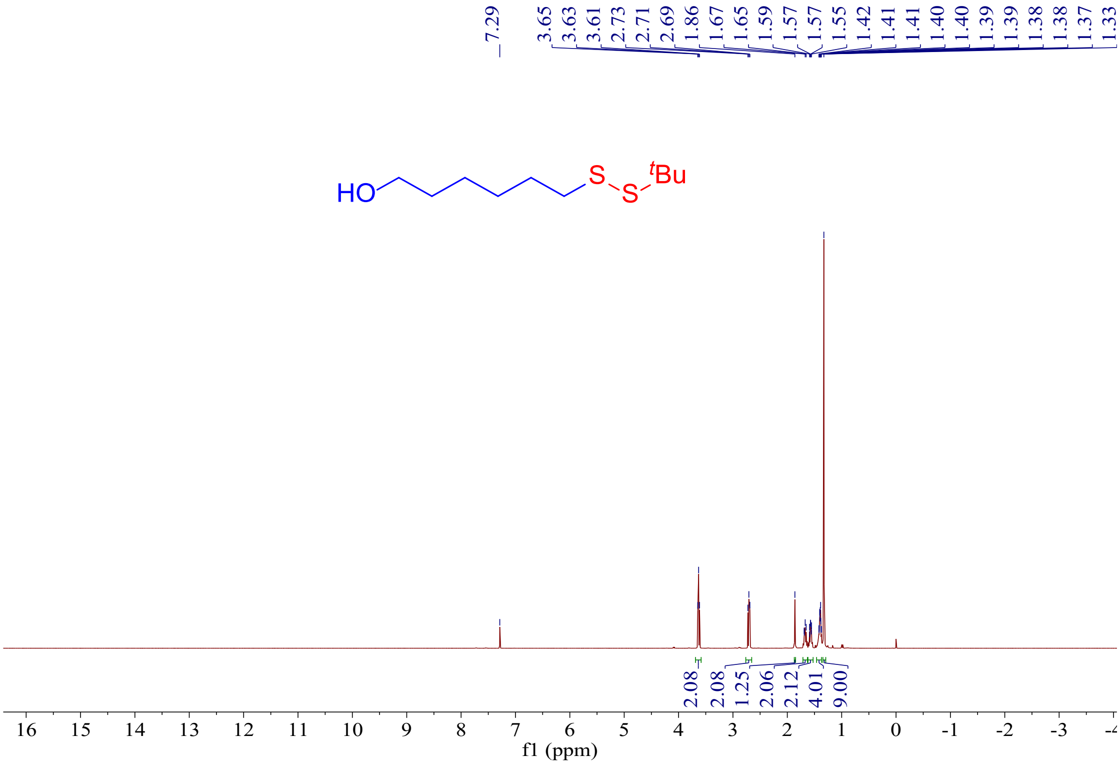

Supplementary Figure 20. ^{1}H NMR spectrum of **3h**.

^{13}C NMR Spectra of **3h** (100 MHz, room temperature, $CDCl_3$)

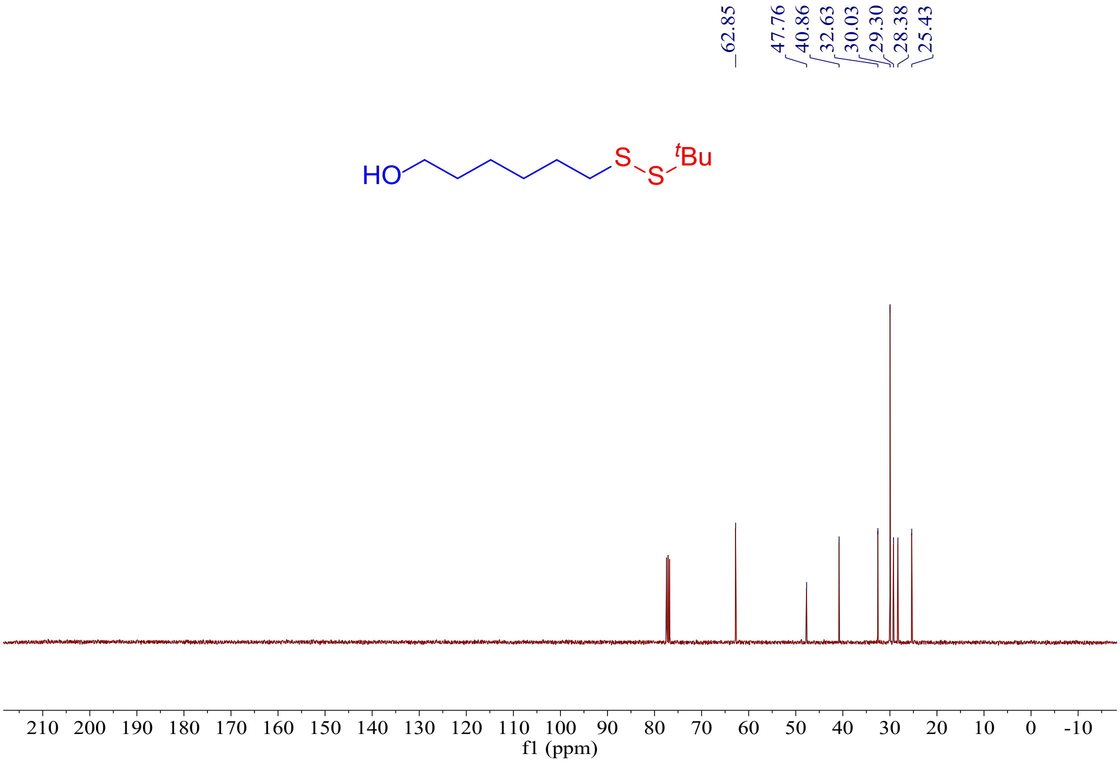

Supplementary Figure 21. ^{13}C NMR spectrum of **3h**.

^1H NMR Spectra of **3i** (400 MHz, room temperature, $CDCl_3$)

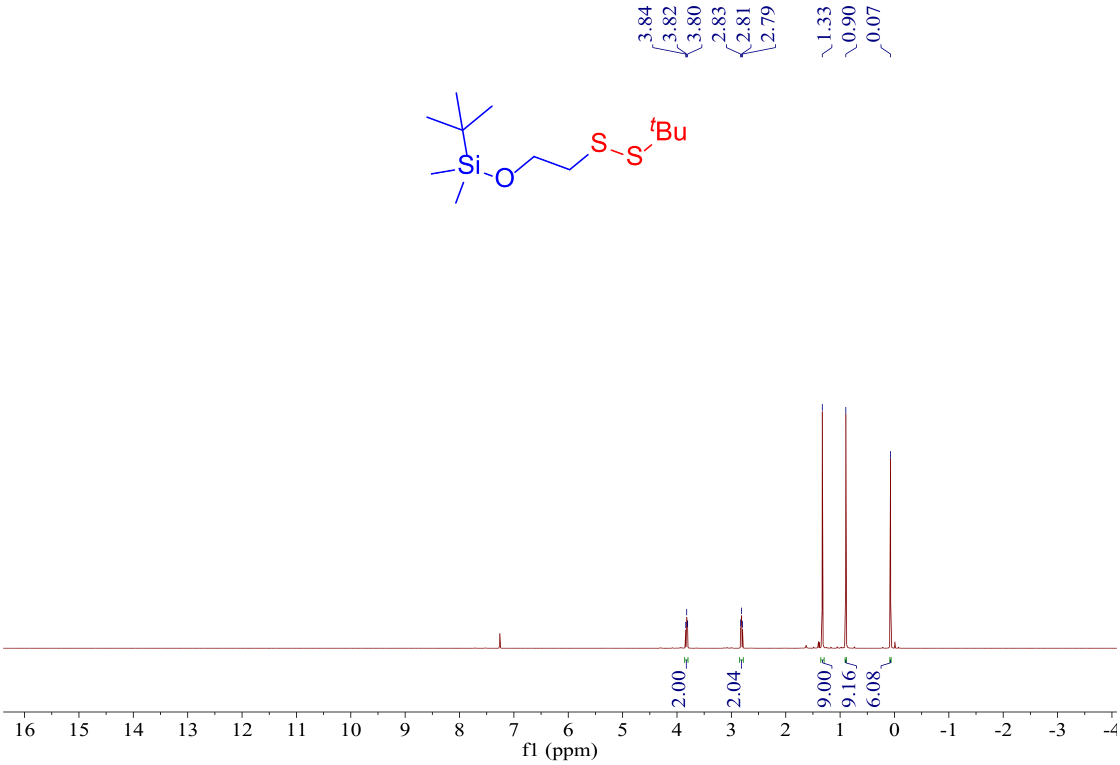

Supplementary Figure 22. ^1H NMR spectrum of **3i**.

^{13}C NMR Spectra of **3i** (100 MHz, room temperature, $CDCl_3$)

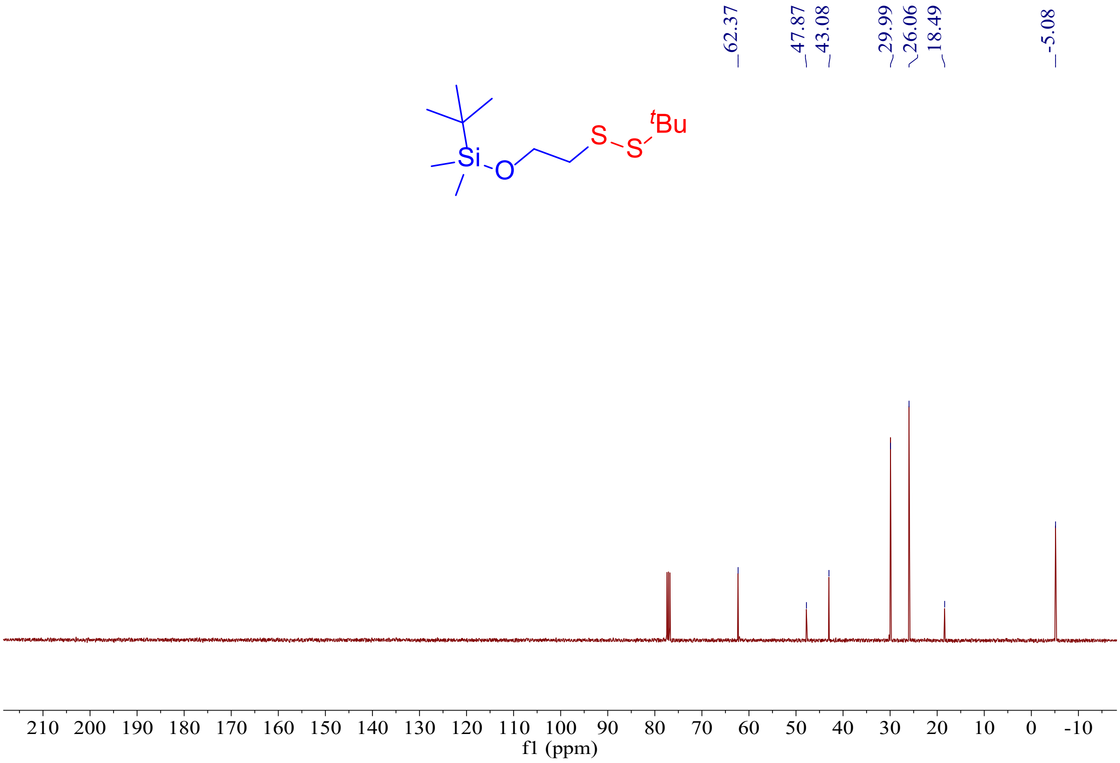

Supplementary Figure 23. ^{13}C NMR spectrum of **3i**.

^{1}H NMR Spectra of **3j** (400 MHz, room temperature, $CDCl_3$)

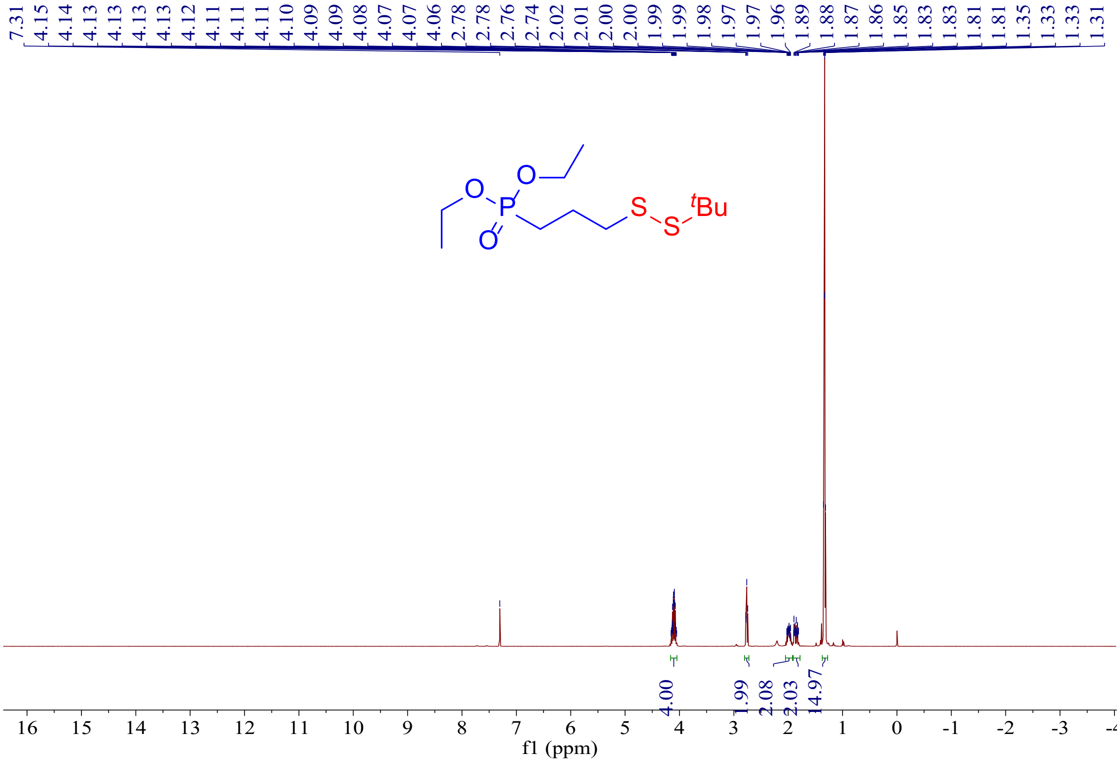

Supplementary Figure 24. ^{1}H NMR spectrum of **3j**.

^{13}C NMR Spectra of **3j** (100 MHz, room temperature, $CDCl_3$)

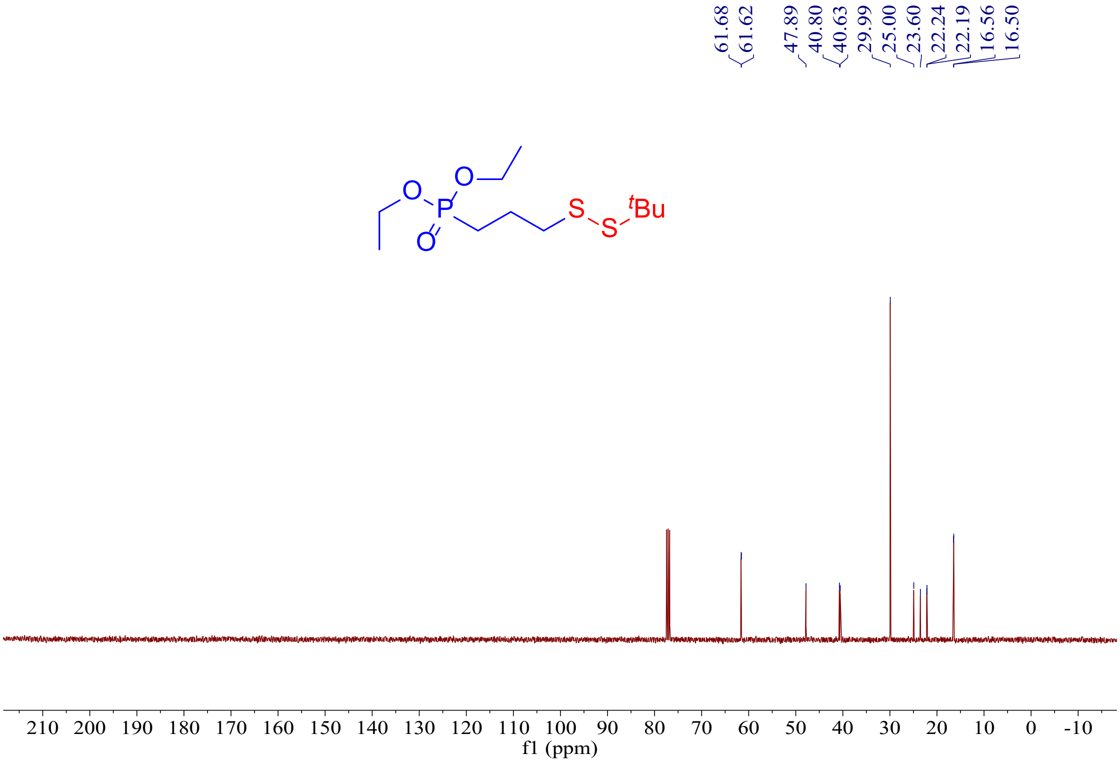

Supplementary Figure 25. ^{13}C NMR spectrum of **3j**.

^{1}H NMR Spectra of **3k** (400 MHz, room temperature, $CDCl_3$)

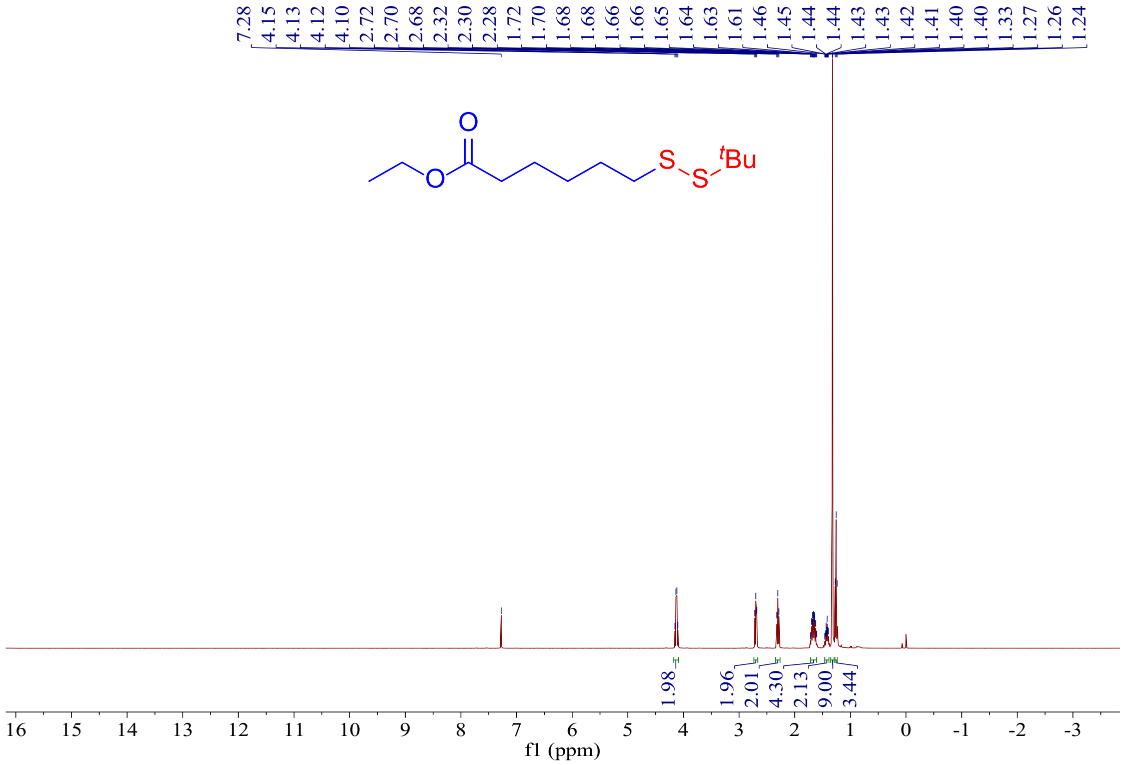

Supplementary Figure 26. ^{1}H NMR spectrum of **3k**.

^{13}C NMR Spectra of **3k** (100 MHz, room temperature, $CDCl_3$)

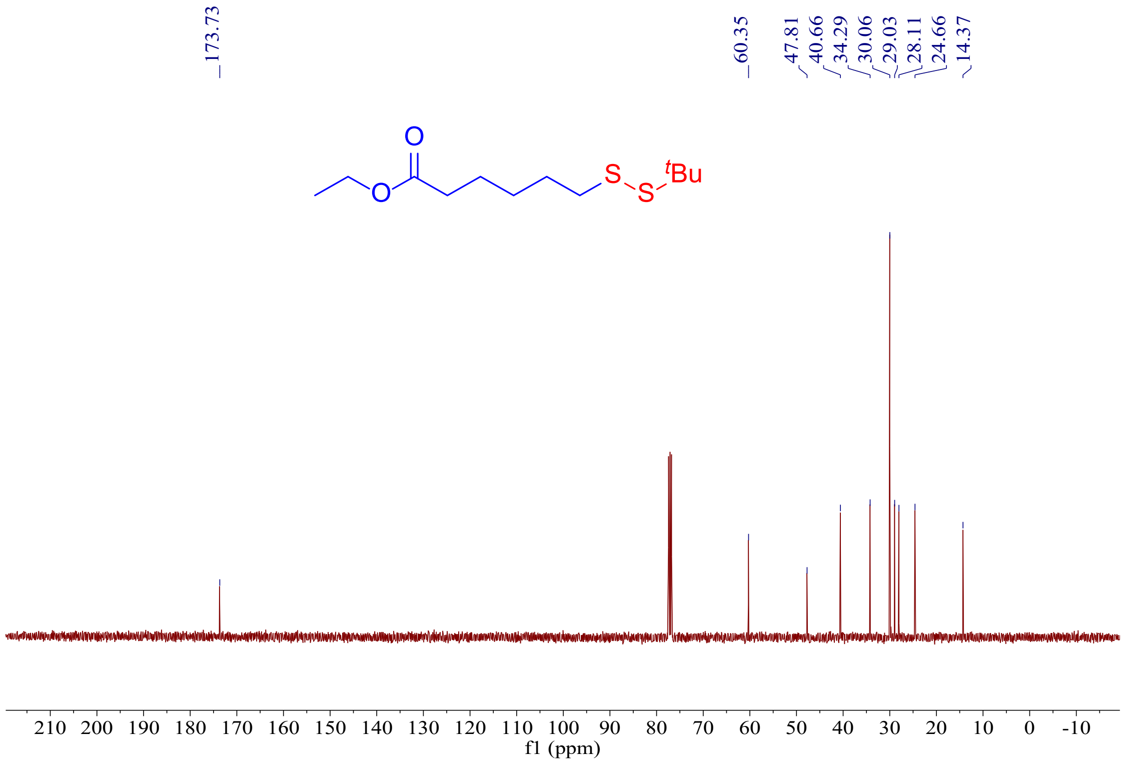

Supplementary Figure 27. ^{13}C NMR spectrum of **3k**.

^{1}H NMR Spectra of **3l** (400 MHz, room temperature, $CDCl_3$)

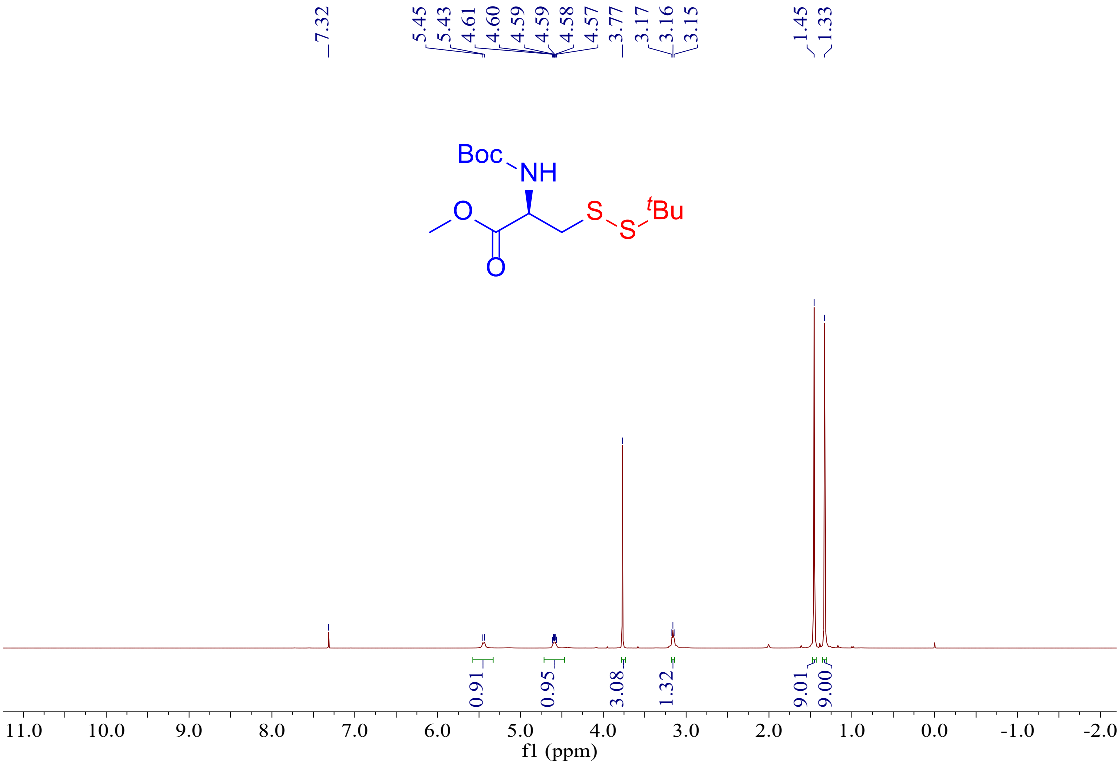

Supplementary Figure 28. ^{1}H NMR spectrum of **3l**.

^{13}C NMR Spectra of **3l** (100 MHz, room temperature, $CDCl_3$)

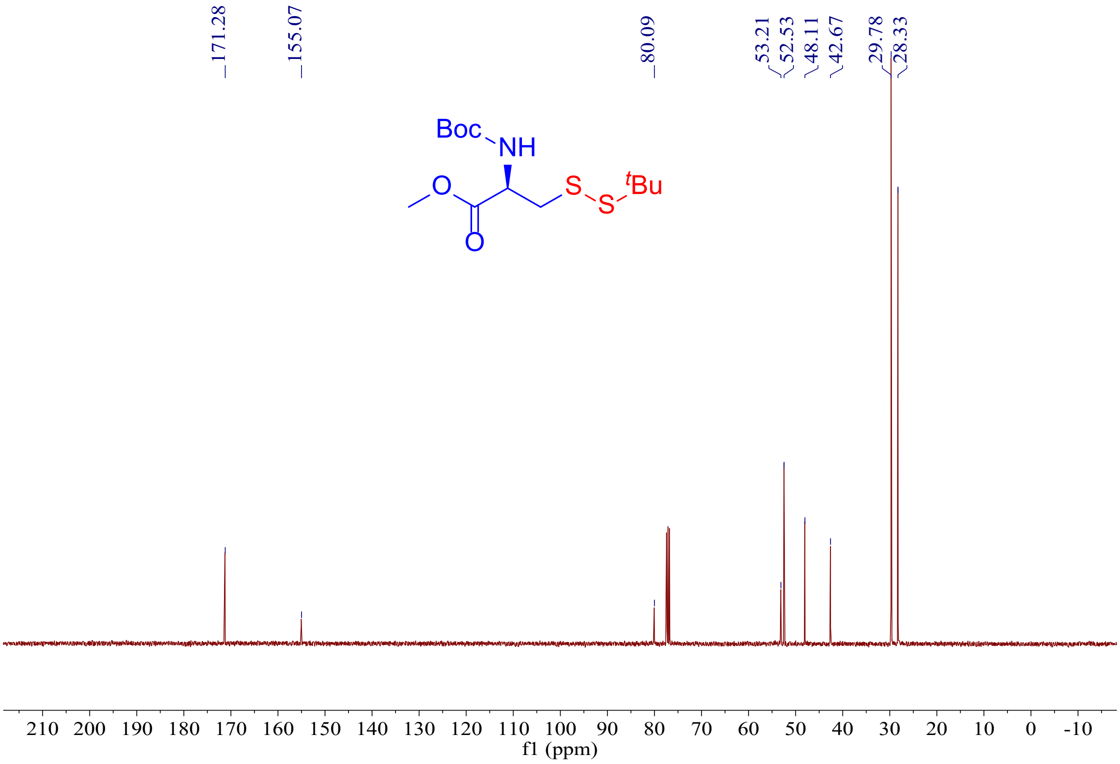

Supplementary Figure 29. ^{13}C NMR spectrum of **3l**.

^{1}H NMR Spectra of **3m** (400 MHz, room temperature, $CDCl_3$)

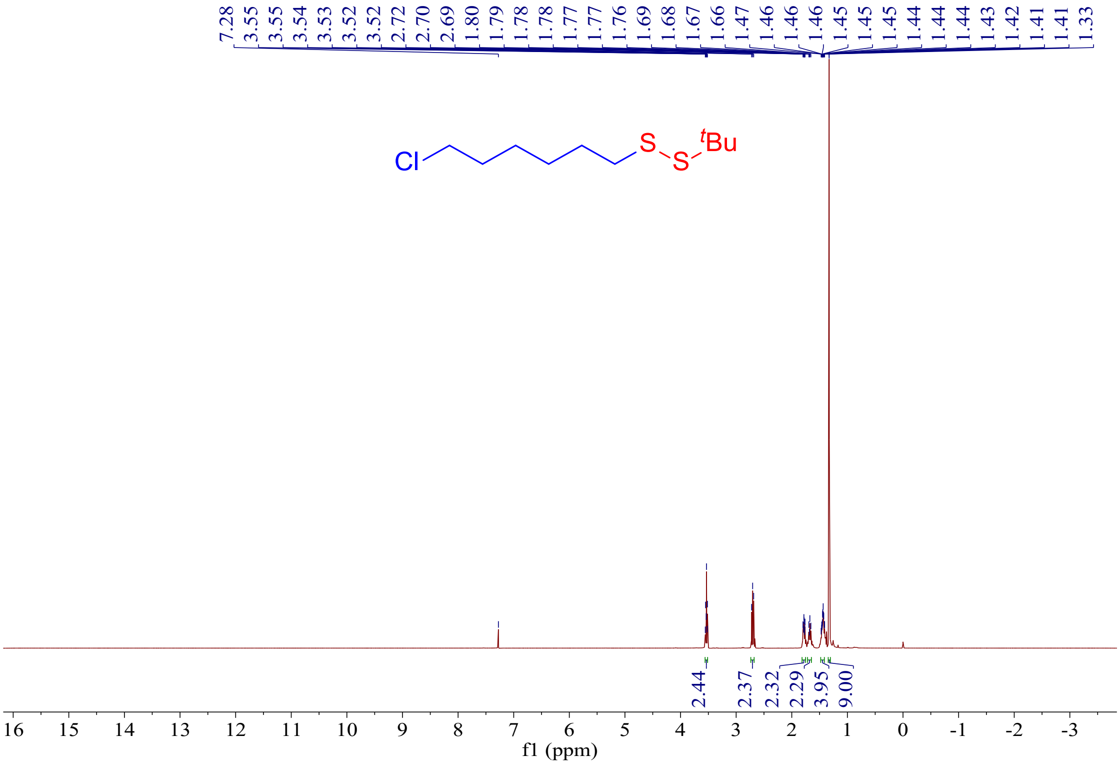

Supplementary Figure 30. ^{1}H NMR spectrum of **3m**.

^{13}C NMR Spectra of **3m** (100 MHz, room temperature, $CDCl_3$)

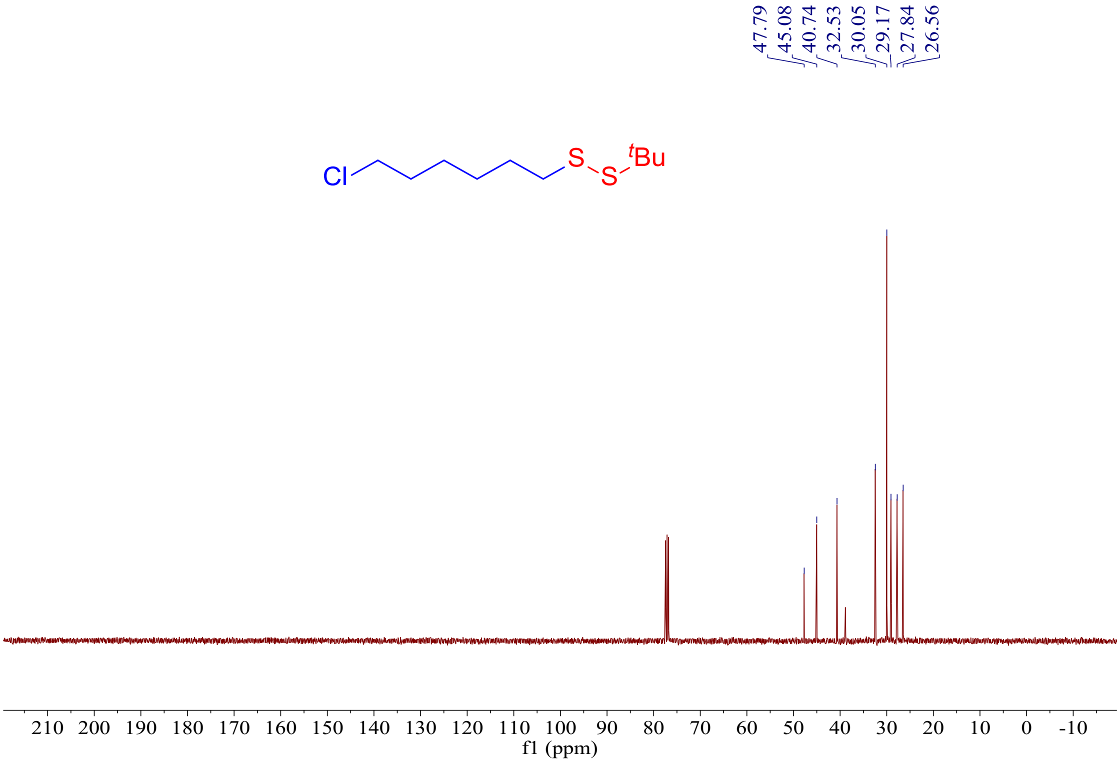

Supplementary Figure 31. ^{13}C NMR spectrum of **3m**.

^{1}H NMR Spectra of **3m'** (400 MHz, room temperature, $CDCl_3$)

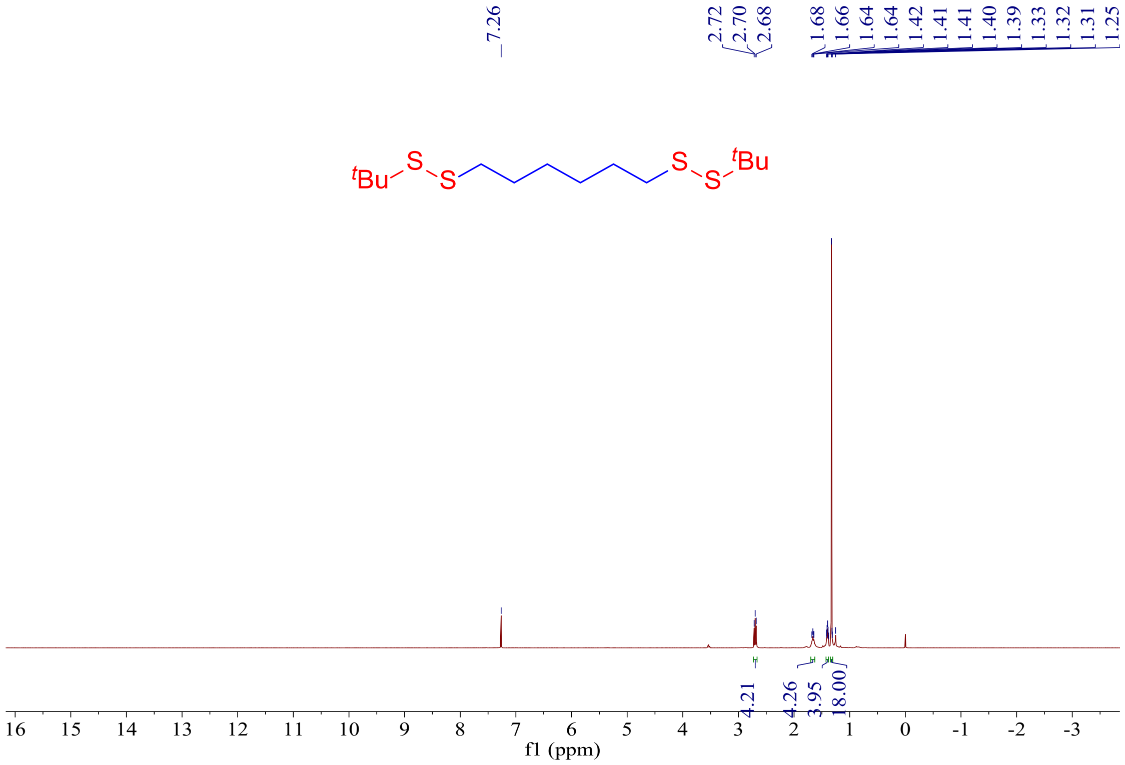

Supplementary Figure 32. ^{1}H NMR spectrum of **3m'**.

^{13}C NMR Spectra of **3m'** (100 MHz, room temperature, $CDCl_3$)

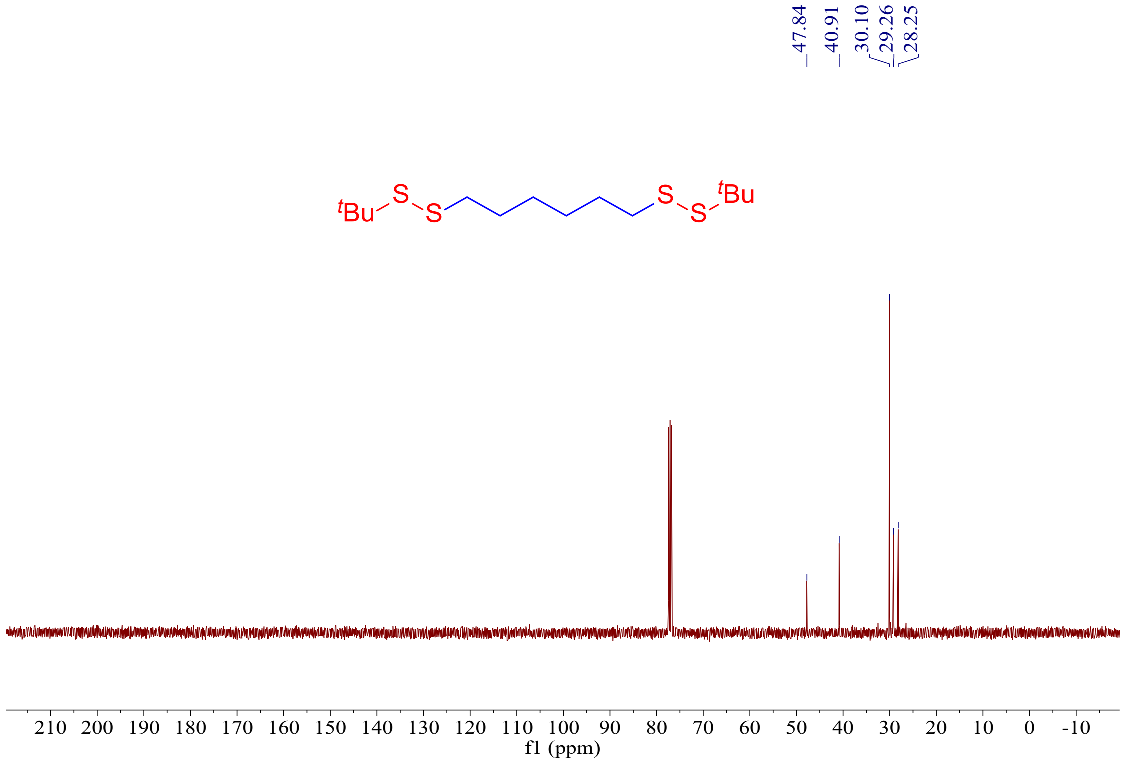

Supplementary Figure 33. ^{13}C NMR spectrum of **3m'**.

^{1}H NMR Spectra of **3n + 3n'** (400 MHz, room temperature, $CDCl_3$)

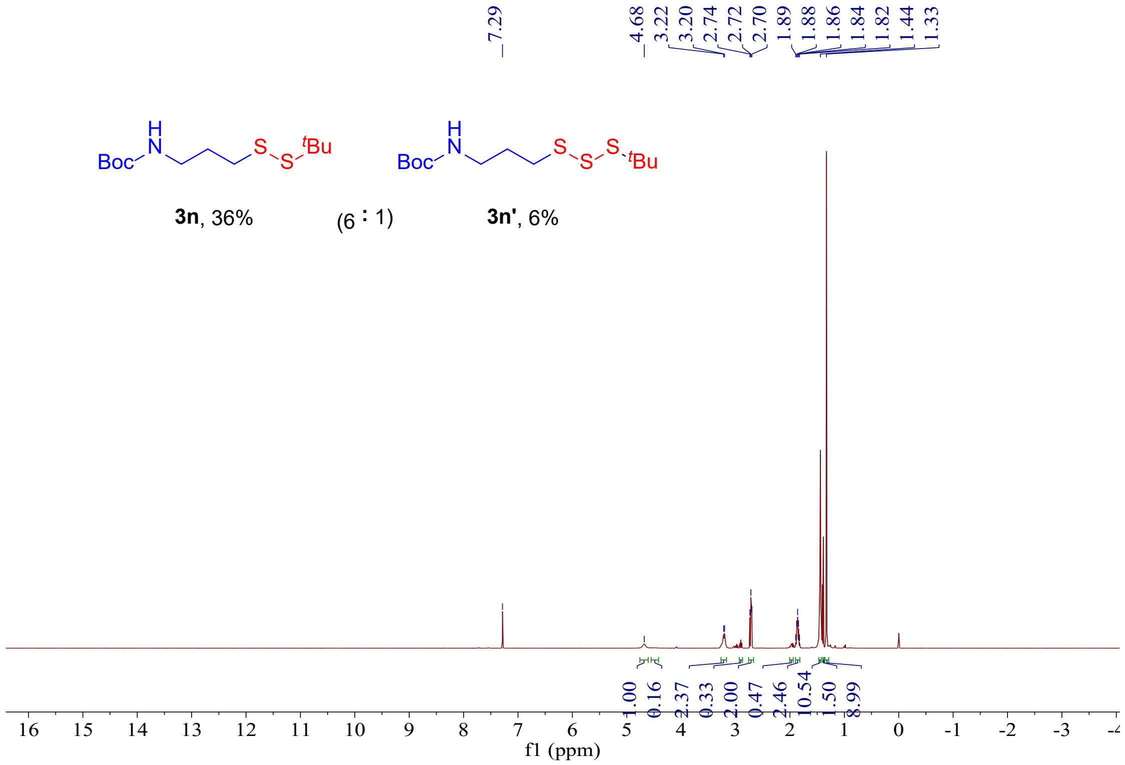

Supplementary Figure 34. ^{1}H NMR spectrum of **3n + 3n'**.

^{13}C NMR Spectra of **3n + 3n'** (100 MHz, room temperature, $CDCl_3$)

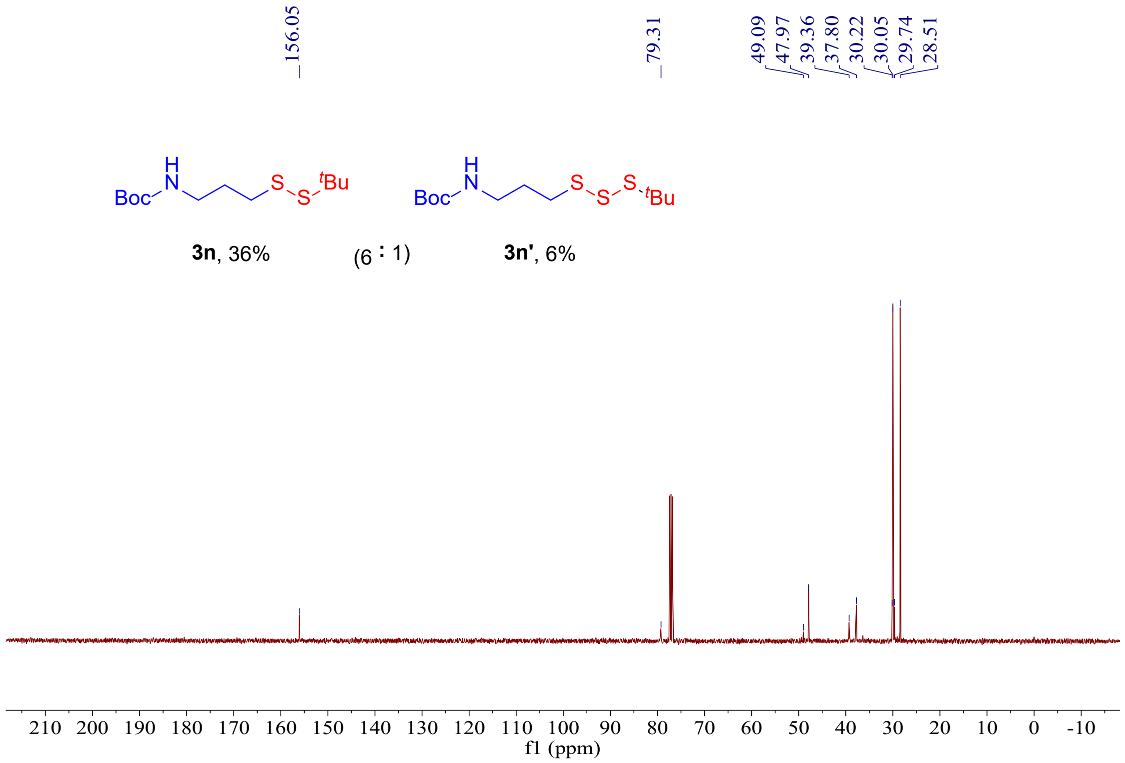

Supplementary Figure 35. ^{13}C NMR spectrum of **3n + 3n'**.

^{1}H NMR Spectra of **3o + 3o’** (400 MHz, room temperature, $CDCl_3$)

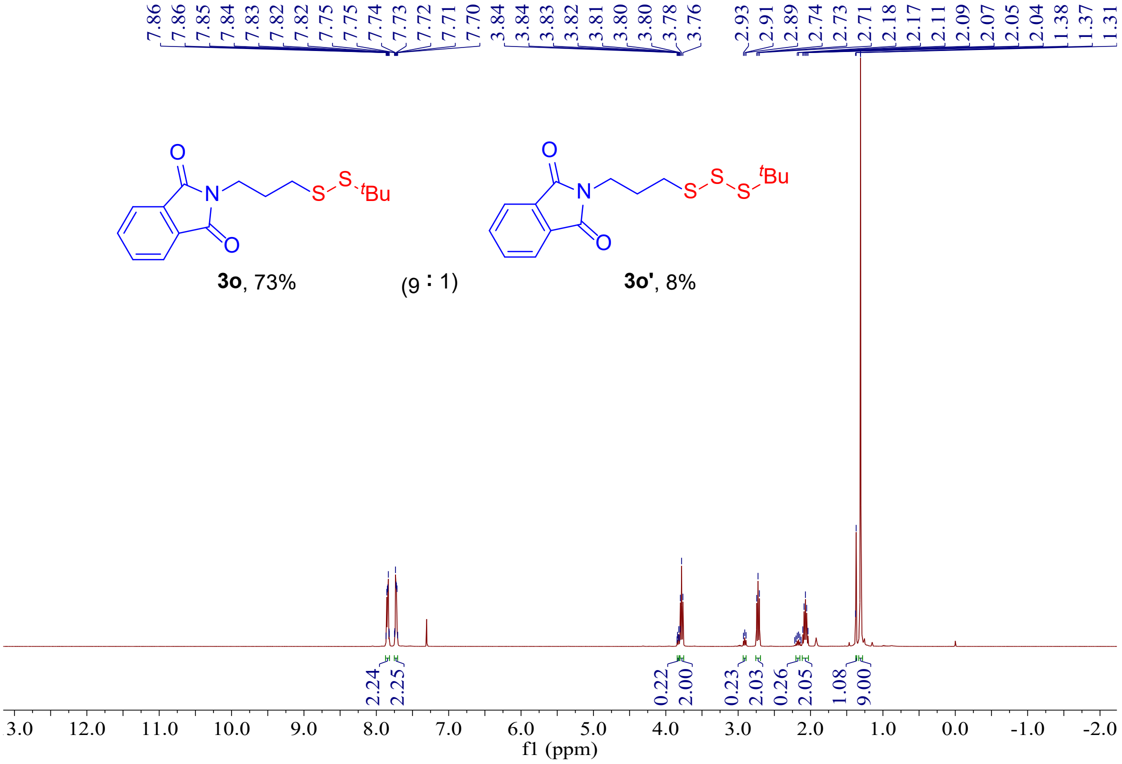

Supplementary Figure 36. ^{1}H NMR spectrum of **3o + 3o’**.

^{13}C NMR Spectra of **3o + 3o’** (100 MHz, room temperature, $CDCl_3$)

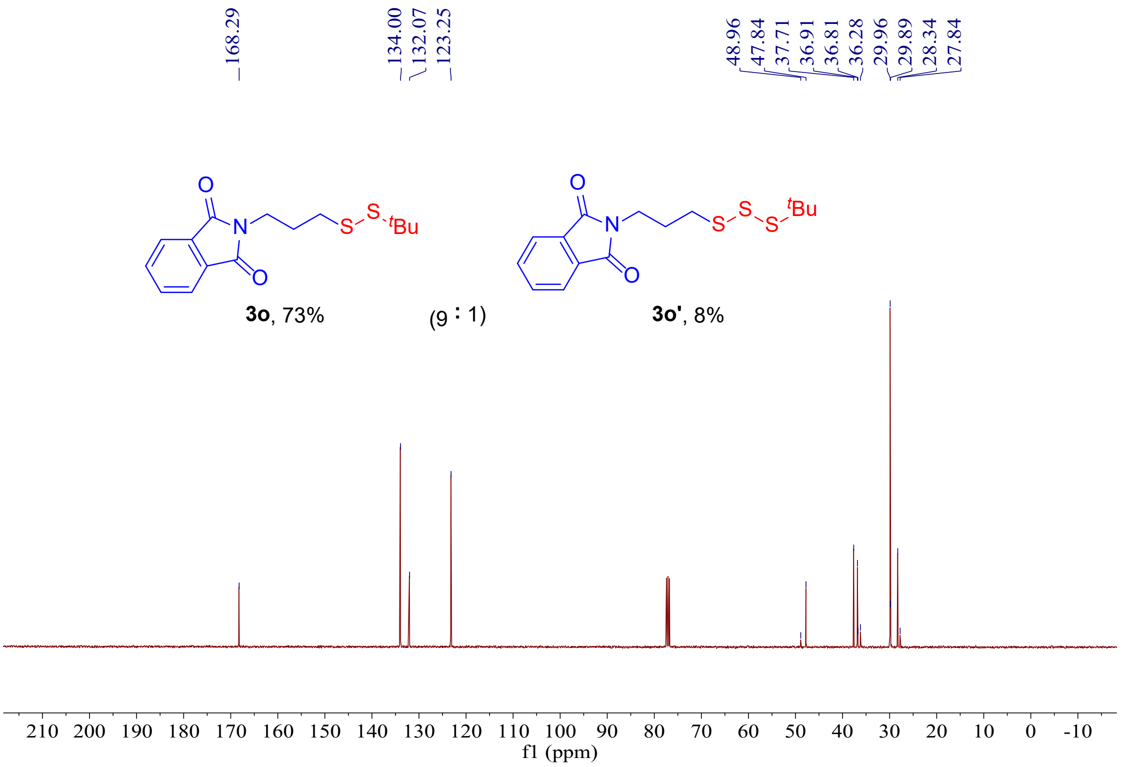

Supplementary Figure 37. ^{13}C NMR spectrum of **3o + 3o’**.

^{1}H NMR Spectra of **3p** (400 MHz, room temperature, $CDCl_3$)

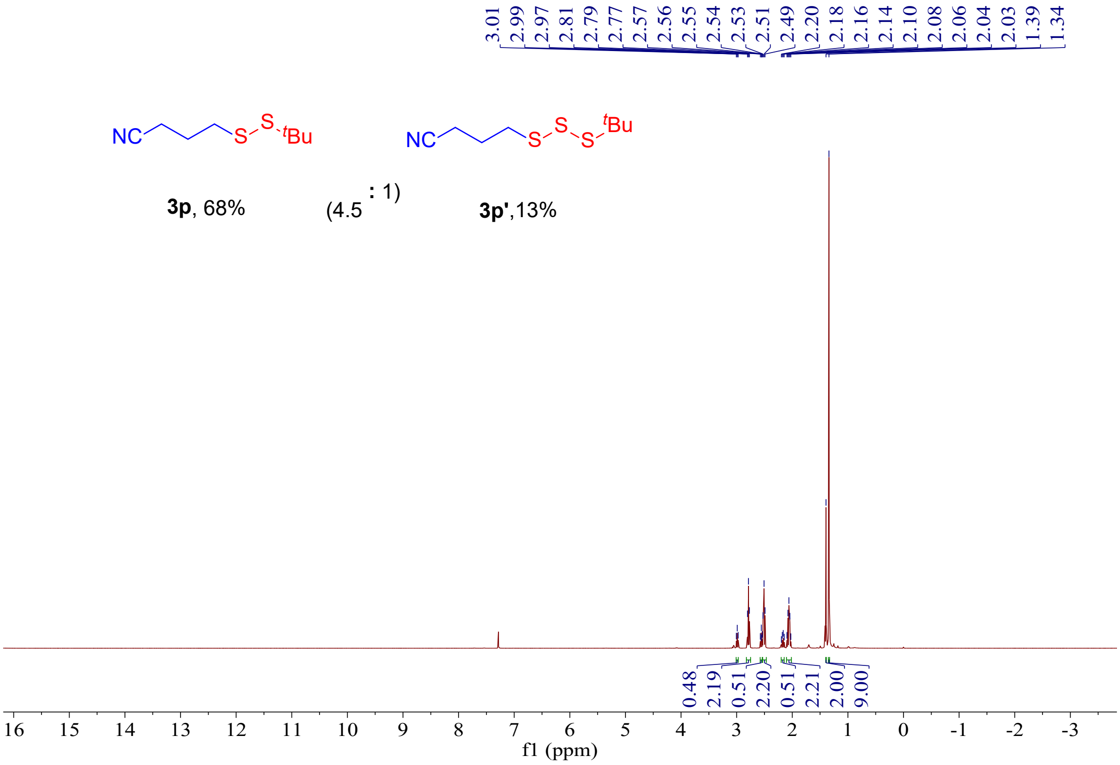

Supplementary Figure 38. ^{1}H NMR spectrum of **3p + 3p'**.

^{13}C NMR Spectra of **3p** (100 MHz, room temperature, $CDCl_3$)

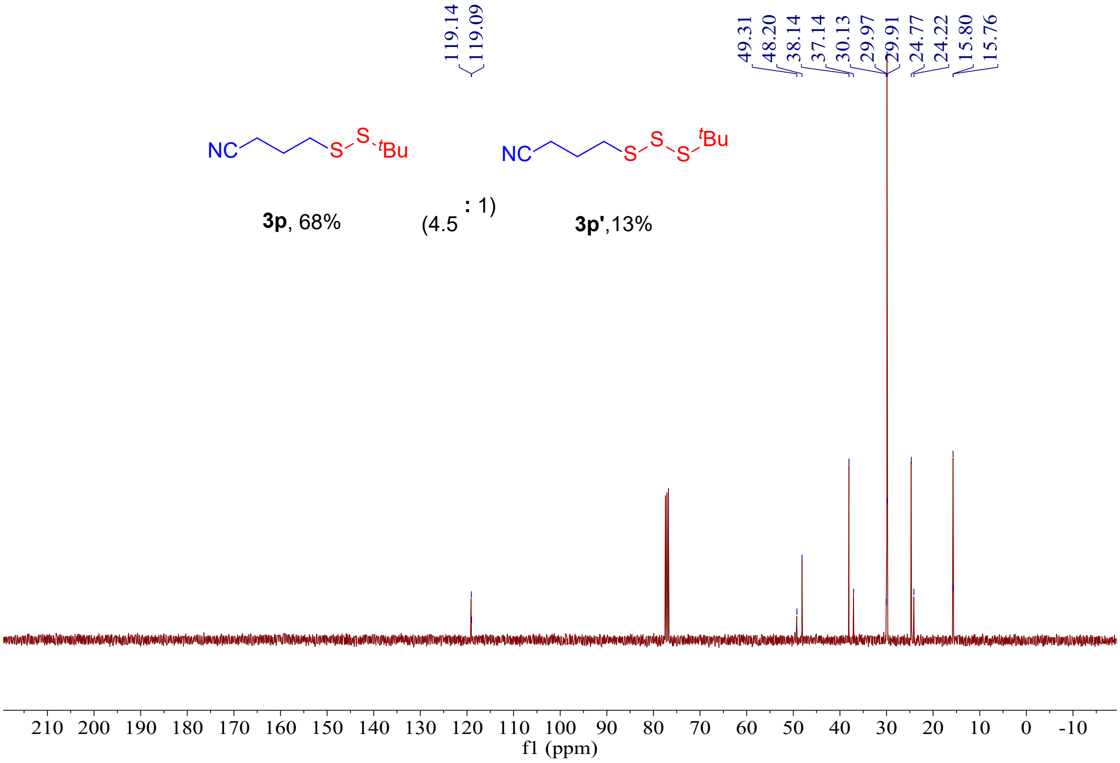

Supplementary Figure 39. ^{13}C NMR spectrum of **3p + 3p'**.

^{1}H NMR Spectra of **4a** (400 MHz, room temperature, $CDCl_3$)

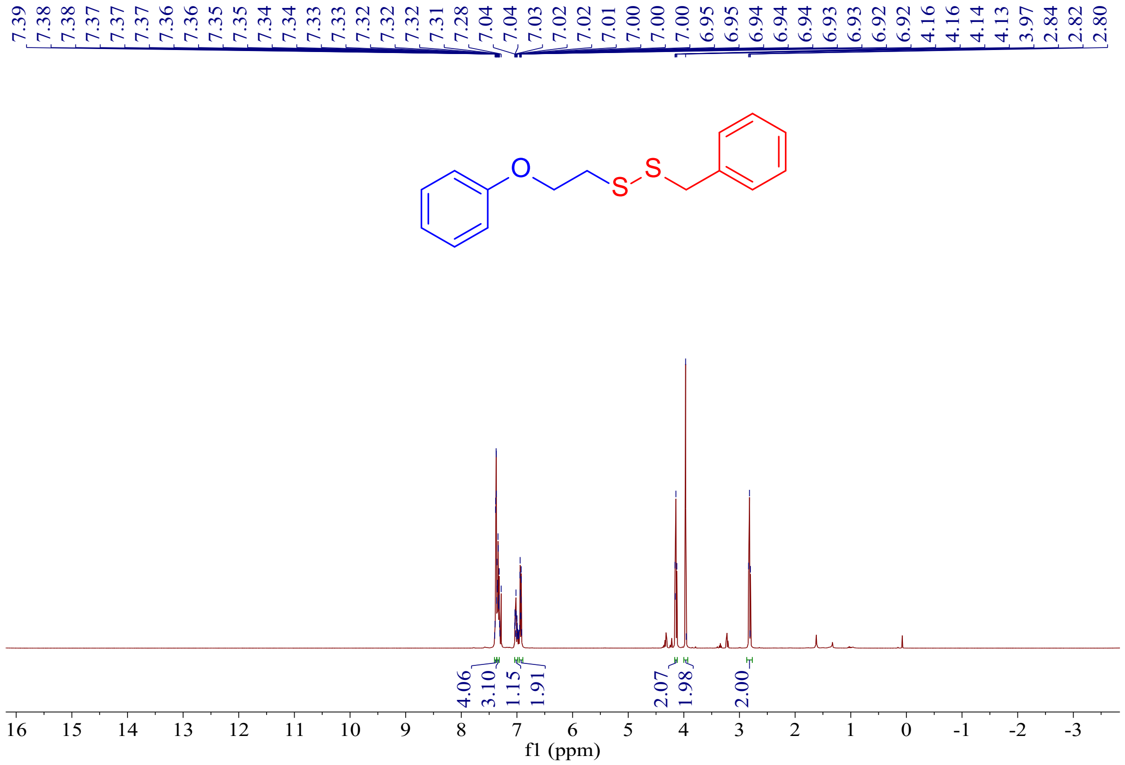

Supplementary Figure 40. ^{1}H NMR spectrum of **4a**.

^{13}C NMR Spectra of **4a** (100 MHz, room temperature, $CDCl_3$)

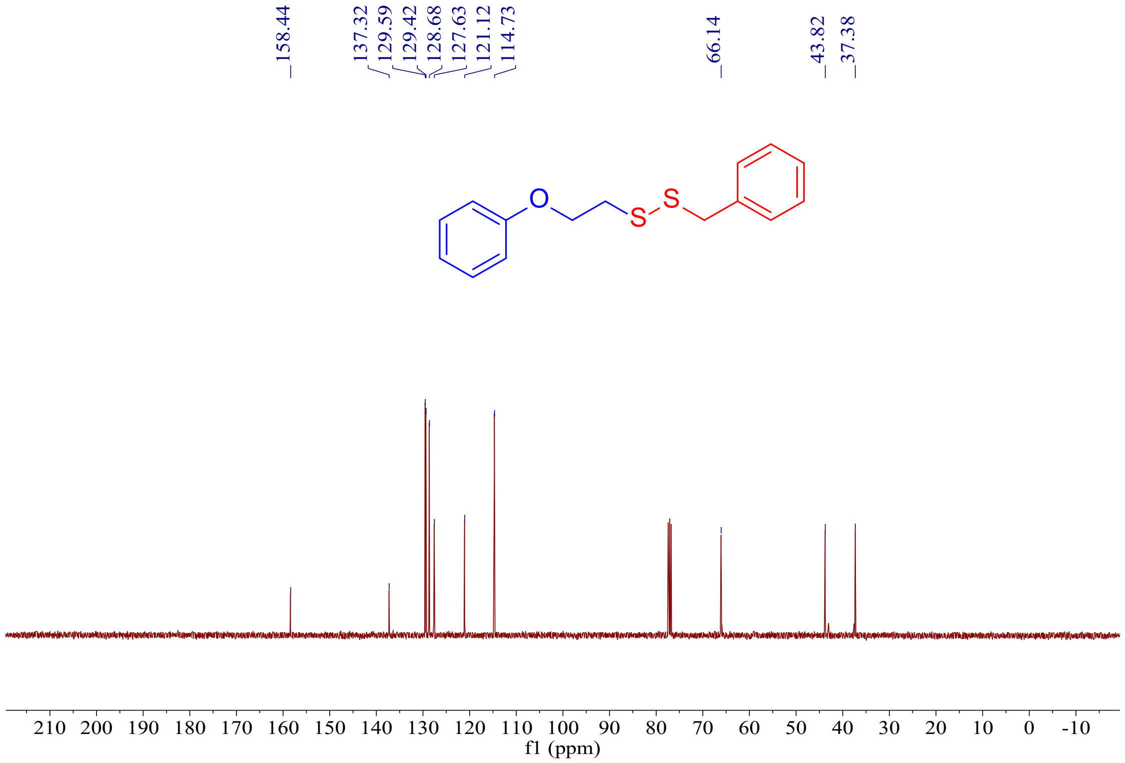

Supplementary Figure 41. ^{13}C NMR spectrum of **4a**.

^{1}H NMR Spectra of **4b** (400 MHz, room temperature, $CDCl_3$)

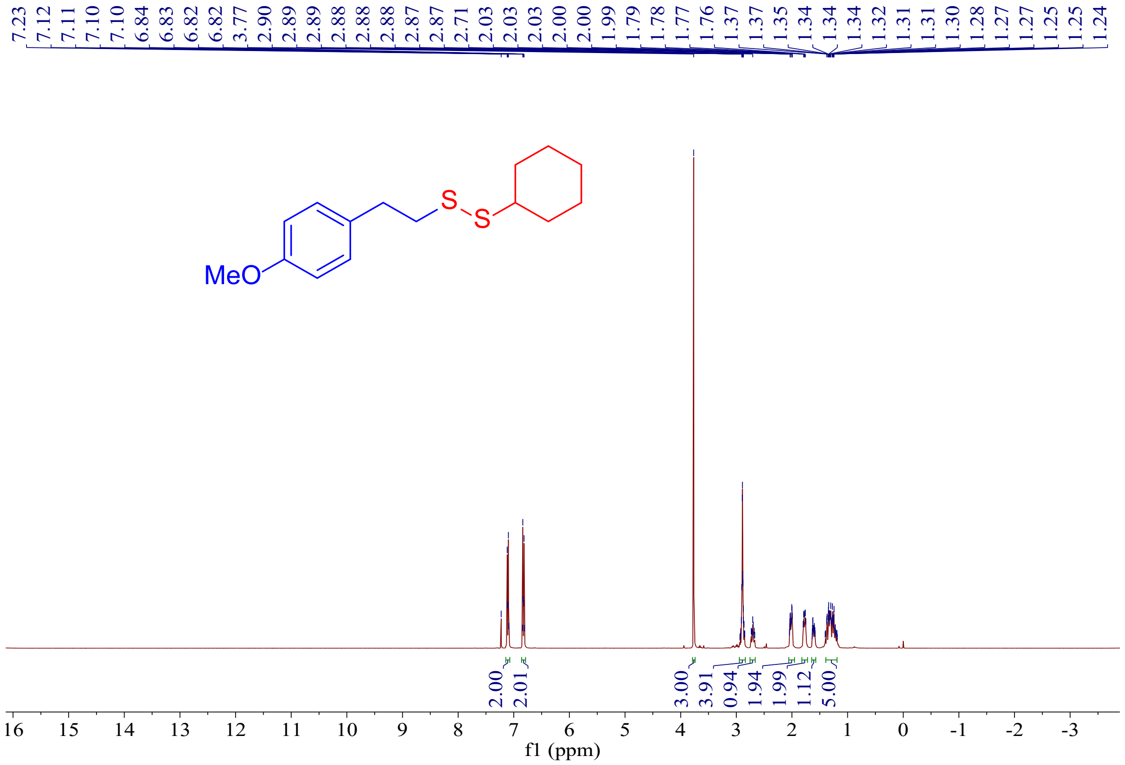

Supplementary Figure 42. ^{1}H NMR spectrum of **4b**.

^{13}C NMR Spectra of **4b** (100 MHz, room temperature, $CDCl_3$)

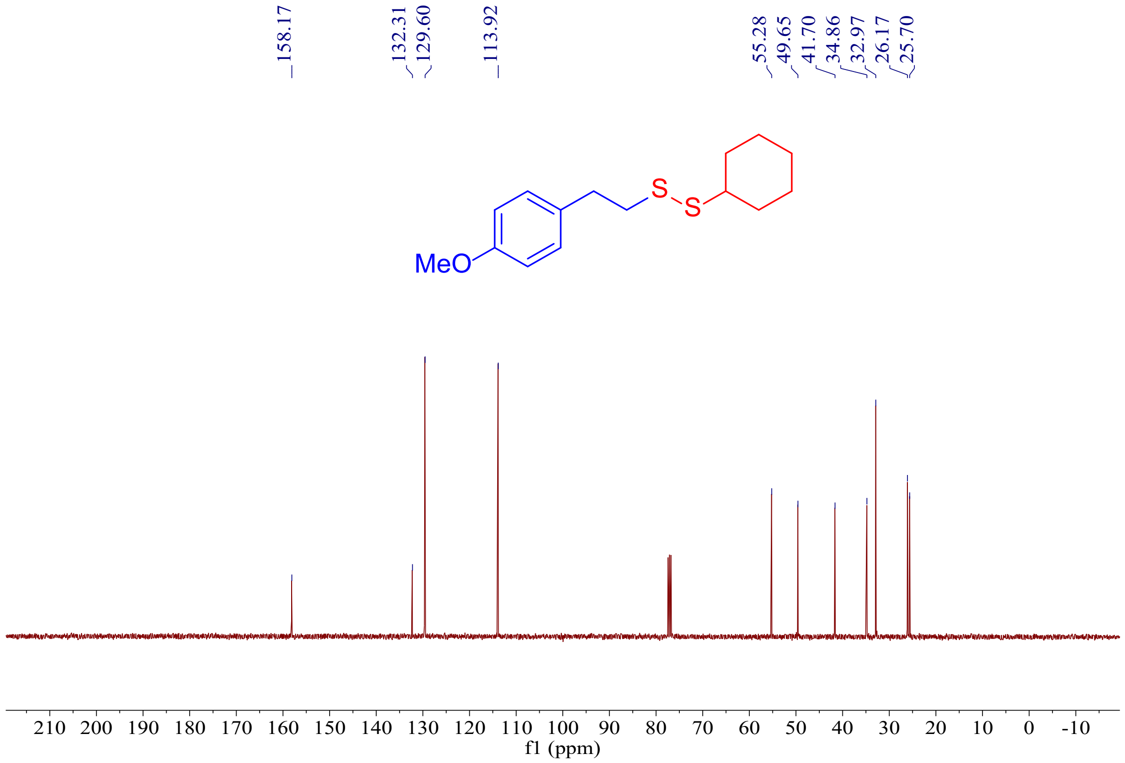

Supplementary Figure 43. ^{13}C NMR spectrum of **4b**.

^{1}H NMR Spectra of **4c** (400 MHz, room temperature, $CDCl_3$)

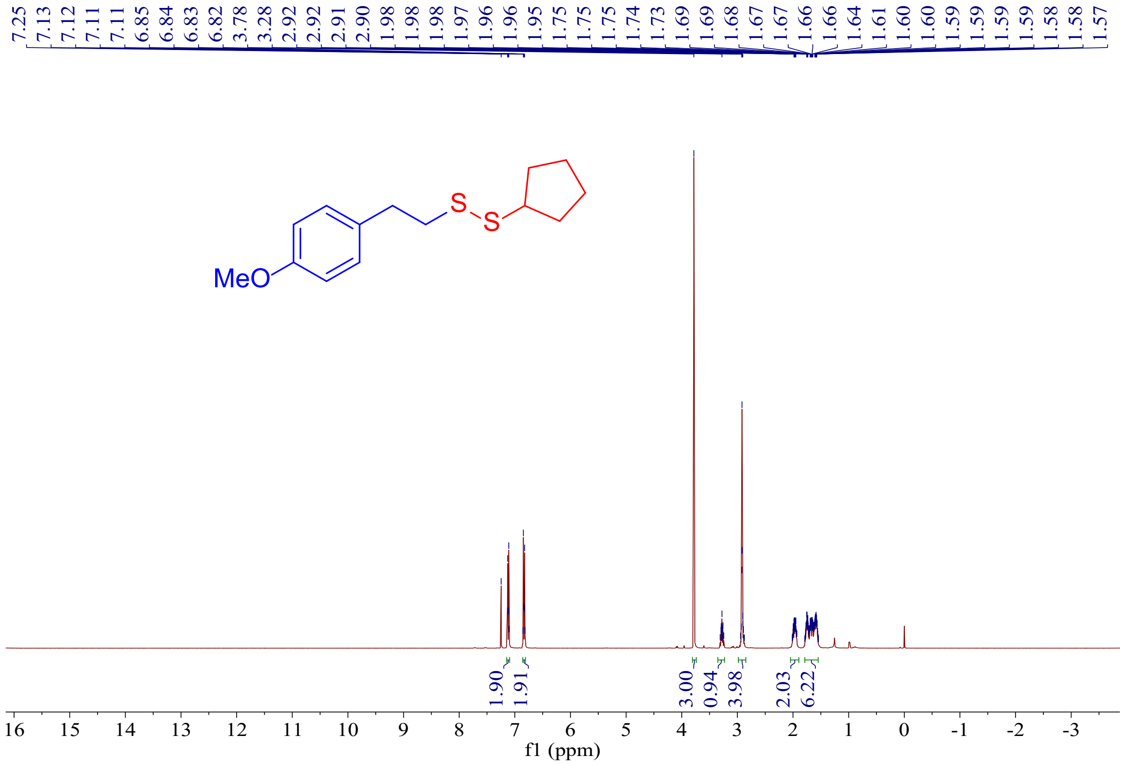

Supplementary Figure 44. ^{1}H NMR spectrum of **4c**.

^{13}C NMR Spectra of **4c** (100 MHz, room temperature, $CDCl_3$)

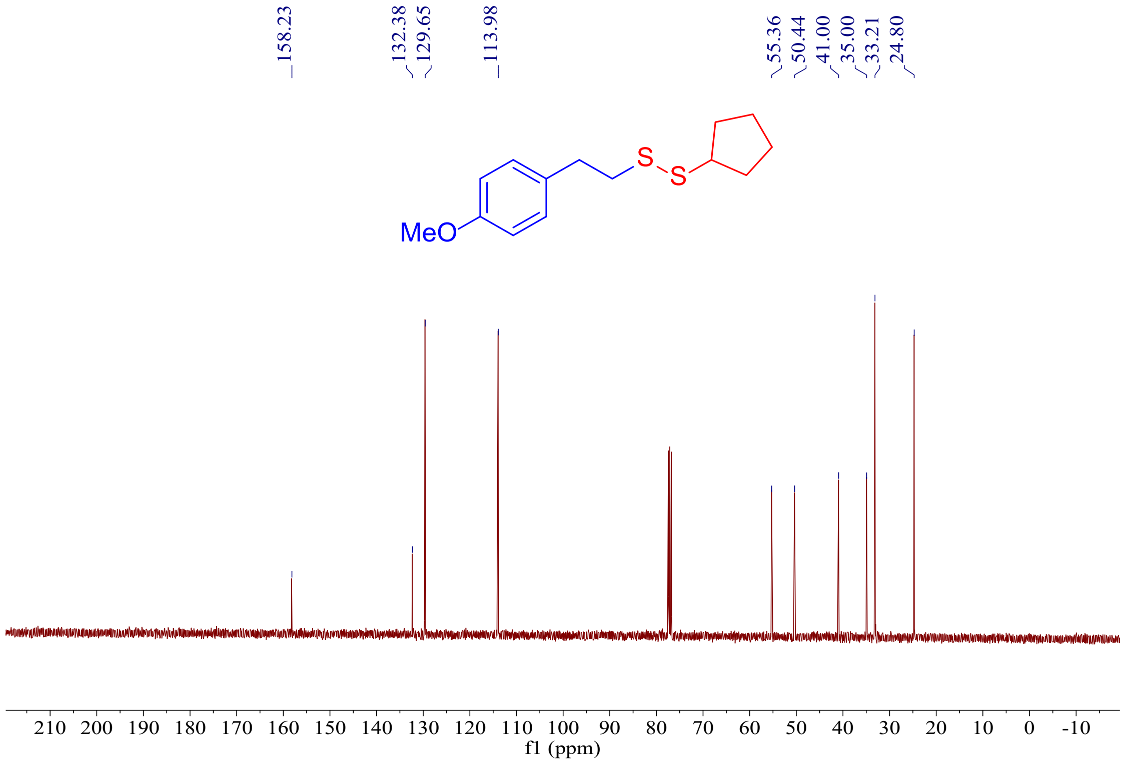

Supplementary Figure 45. ^{13}CMR spectrum of **4c**.

^{1}H NMR Spectra of **4d** (400 MHz, room temperature, $CDCl_3$)

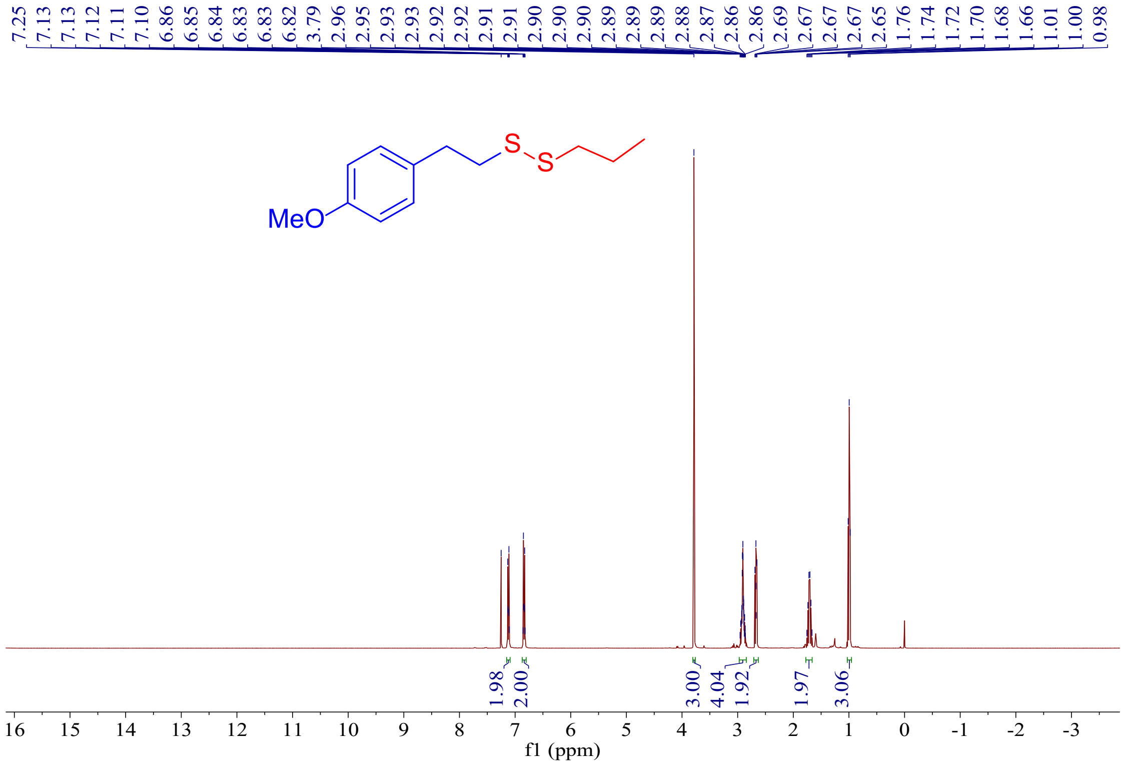

Supplementary Figure 46. ^{1}H NMR spectrum of **4d**.

^{13}C NMR Spectra of **4d** (100 MHz, room temperature, $CDCl_3$)

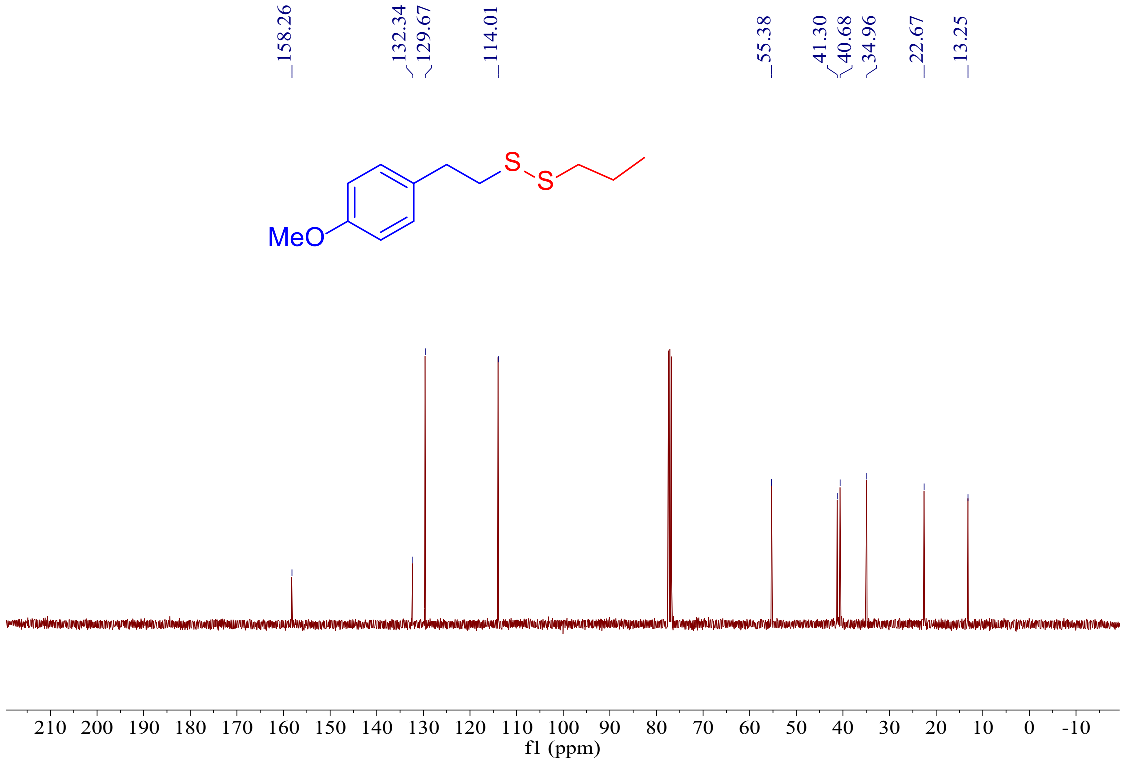

Supplementary Figure 47. ^{13}C NMR spectrum of **4d**.

^{1}H NMR Spectra of **4e** (400 MHz, room temperature, $CDCl_3$)

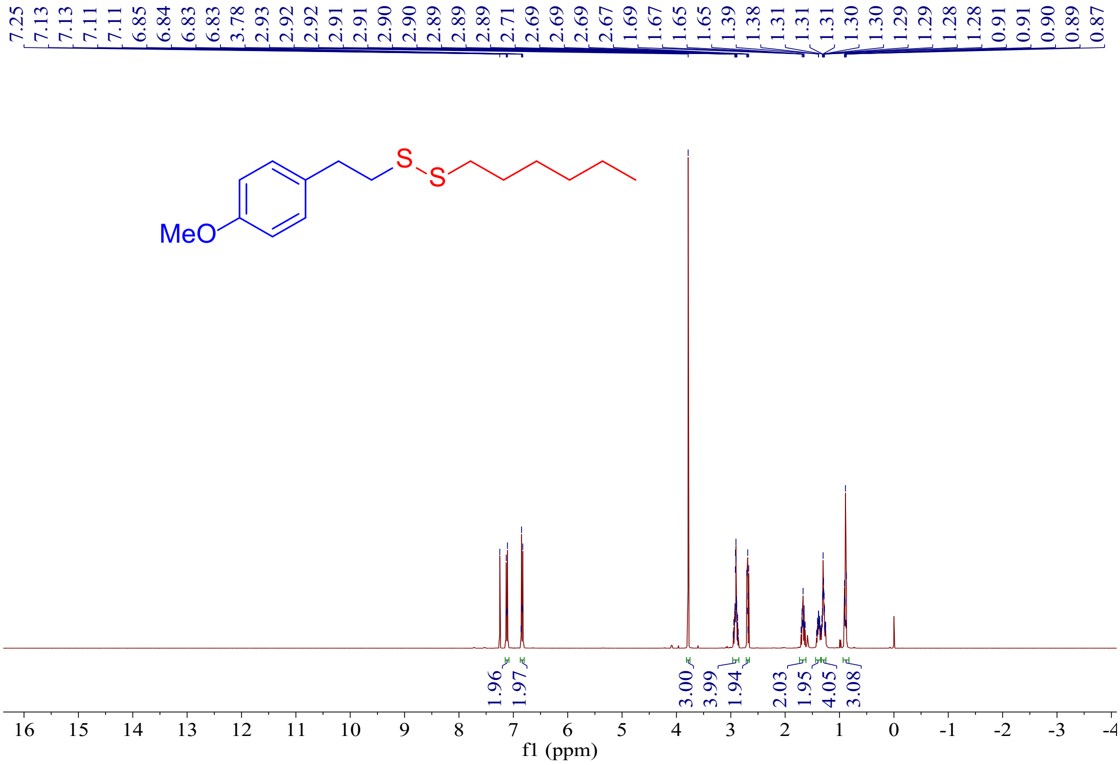

Supplementary Figure 48. ^{1}H NMR spectrum of **4e**.

^{13}C NMR Spectra of **4e** (100 MHz, room temperature, $CDCl_3$)

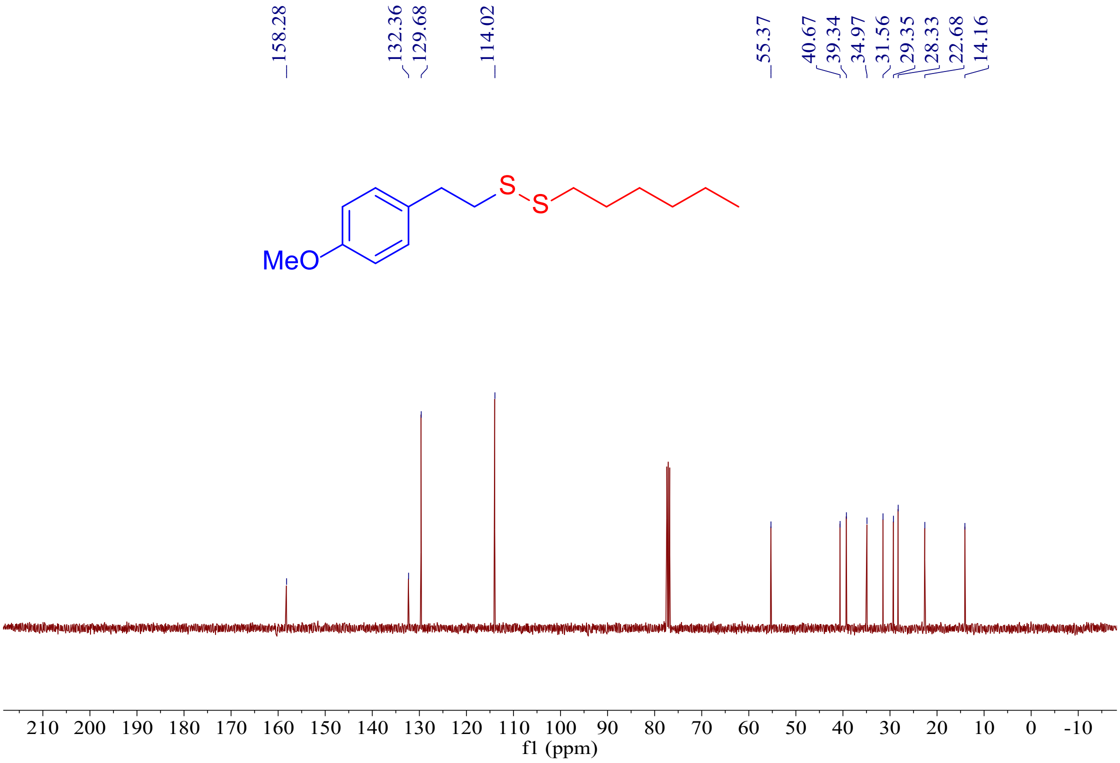

Supplementary Figure 49. ^{13}C NMR spectrum of **4e**.

^{1}H NMR Spectra of **4f** (400 MHz, room temperature, $CDCl_3$)

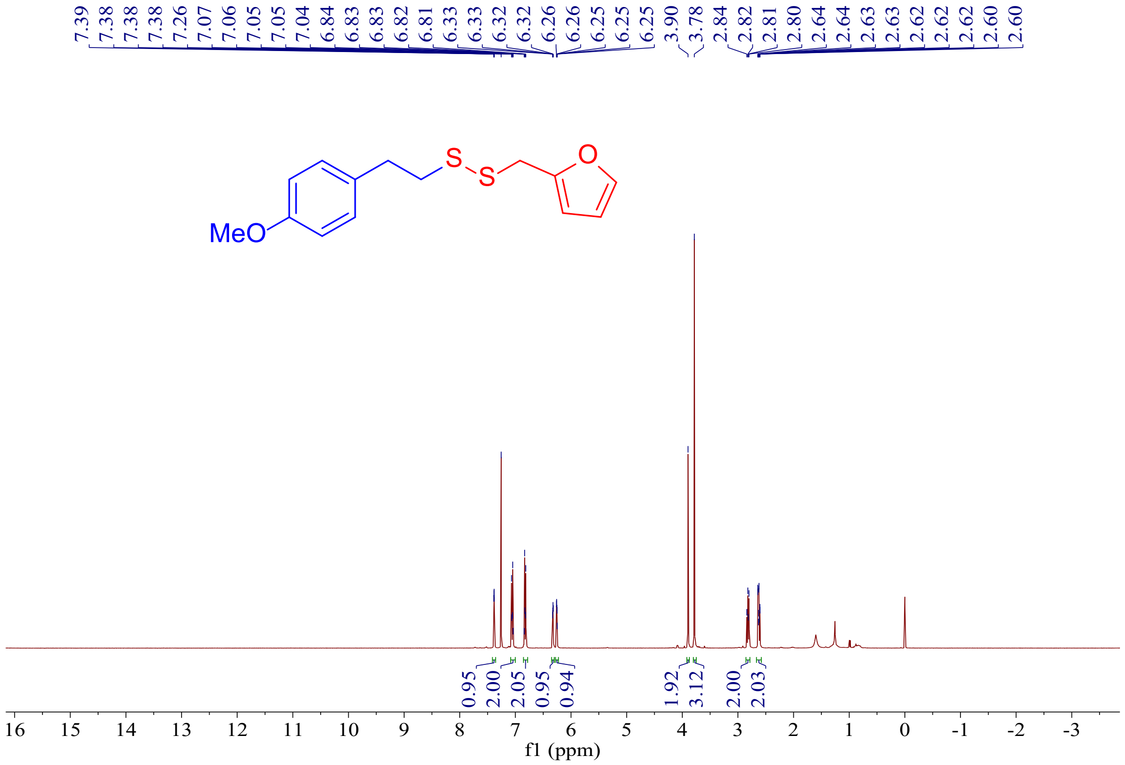

Supplementary Figure 50. ^{1}H NMR spectrum of **4f**.

^{13}C NMR Spectra of **4f** (100 MHz, room temperature, $CDCl_3$)

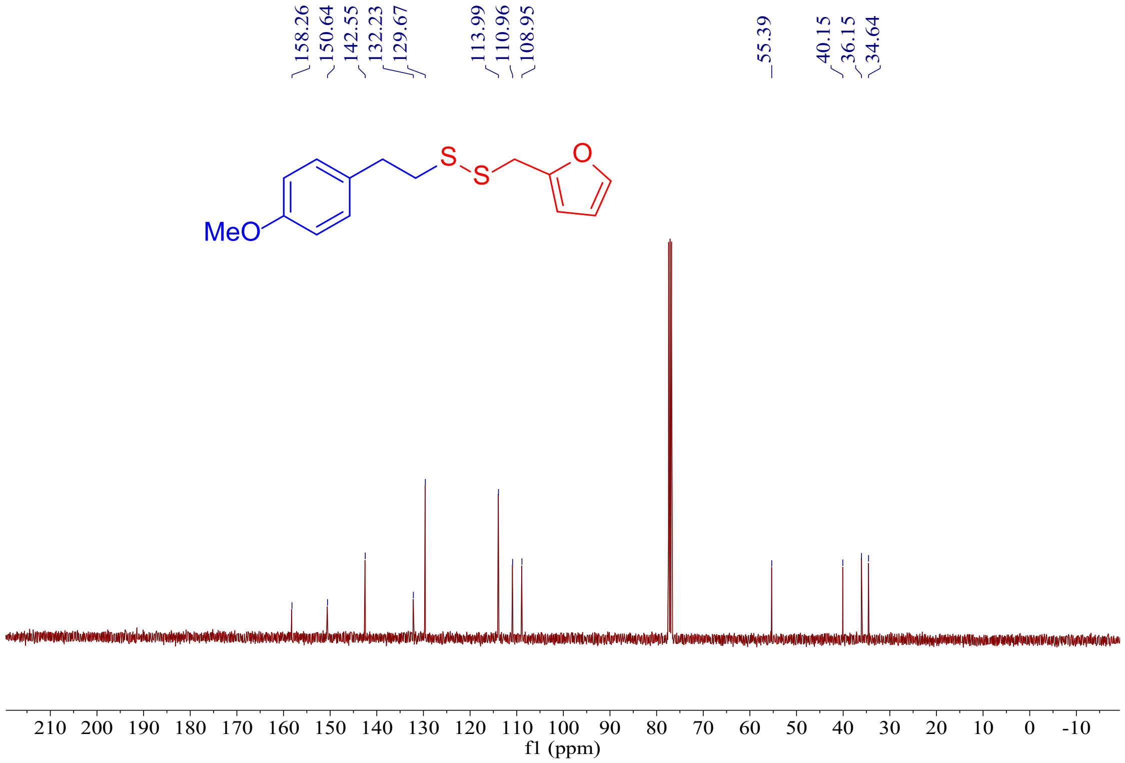

Supplementary Figure 51. ^{13}C NMR spectrum of **4f**.

^{1}H NMR Spectra of **4g + 4g’** (100 MHz, room temperature, $CDCl_3$)

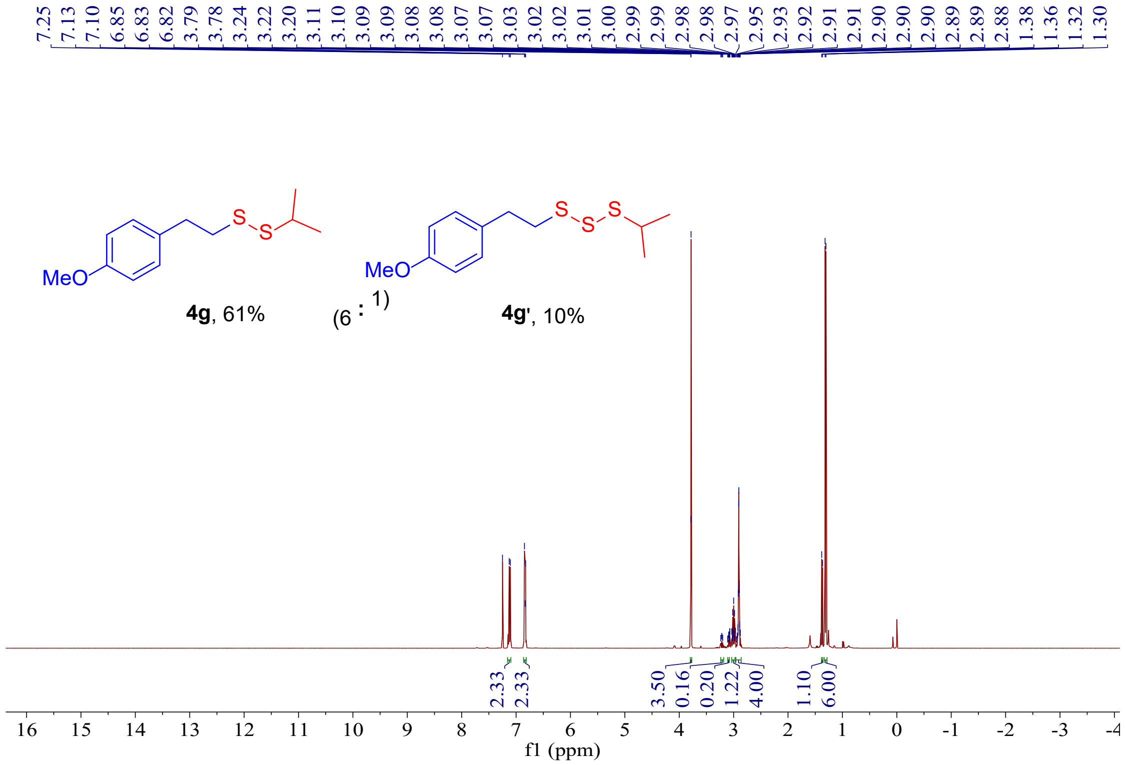

Supplementary Figure 52. ^{1}H NMR spectrum of **4g + 4g’**.

^{13}C NMR Spectra of **4g + 4g’** (100 MHz, room temperature, $CDCl_3$)

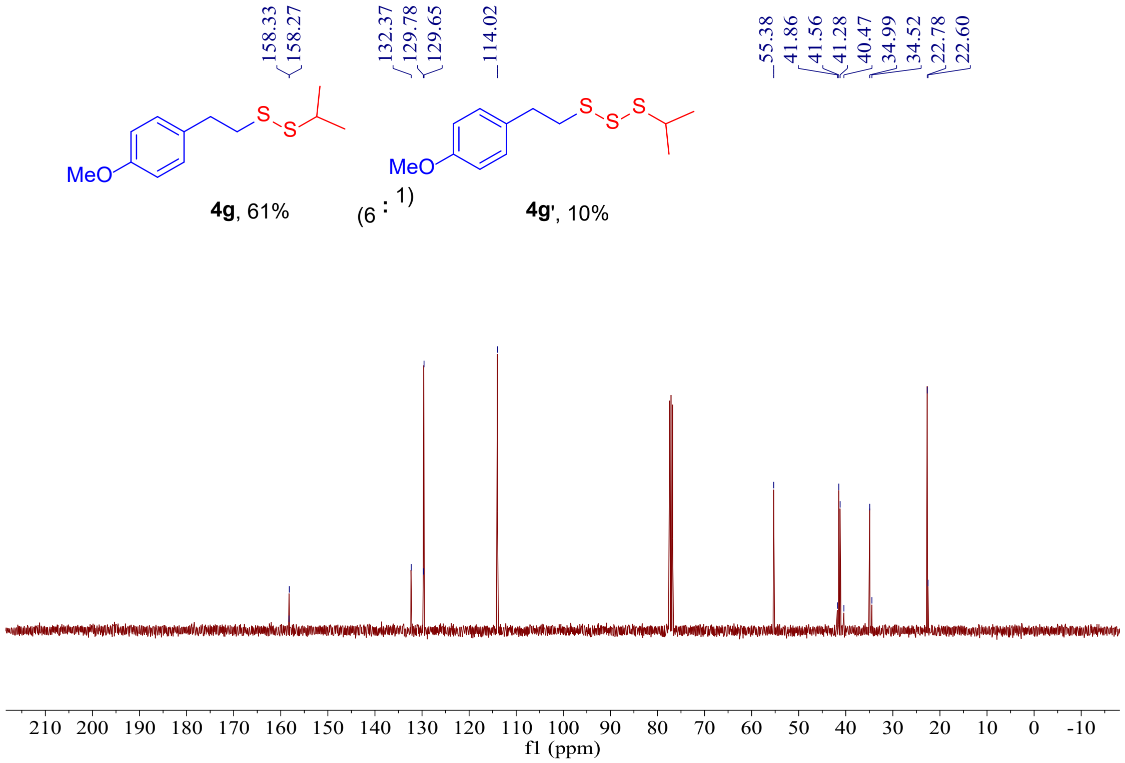

Supplementary Figure 53. ^{13}C NMR spectrum of **4g + 4g’**.

^{1}H NMR Spectra of **4h +4h’** (400 MHz, room temperature, $CDCl_3$)

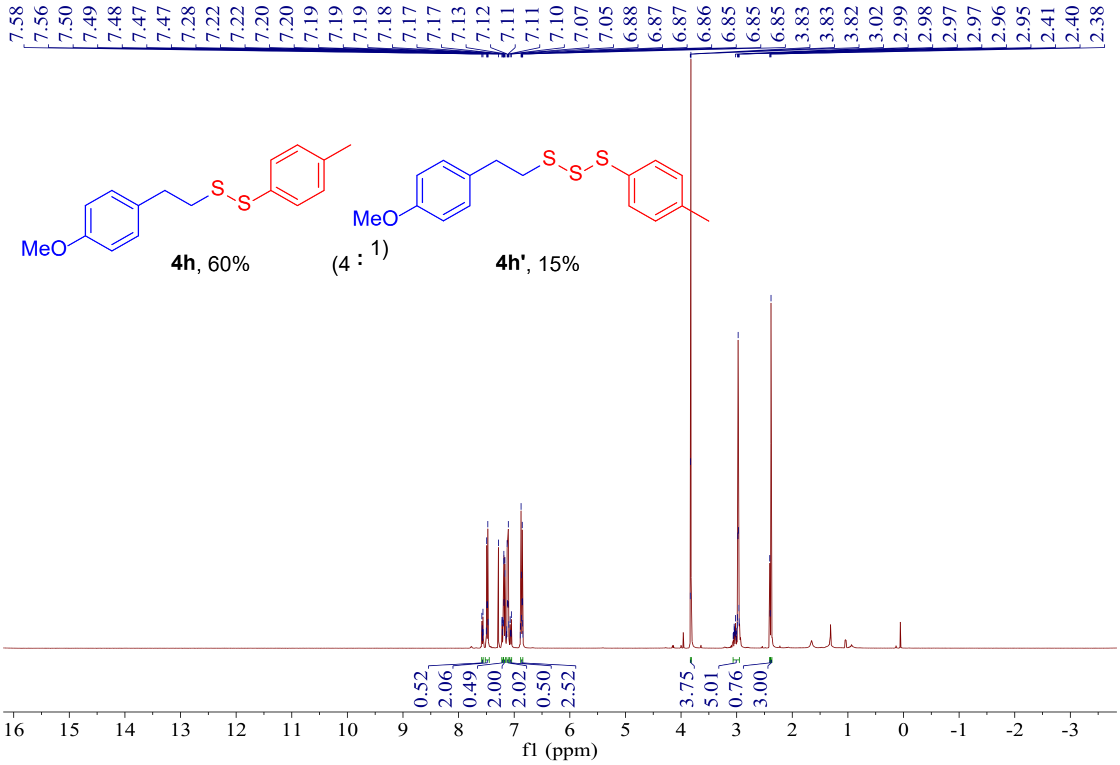

Supplementary Figure 54. ^{1}H NMR spectrum of **4h +4h’**.

^{13}C NMR Spectra of **5h** (100 MHz, room temperature, $CDCl_3$)

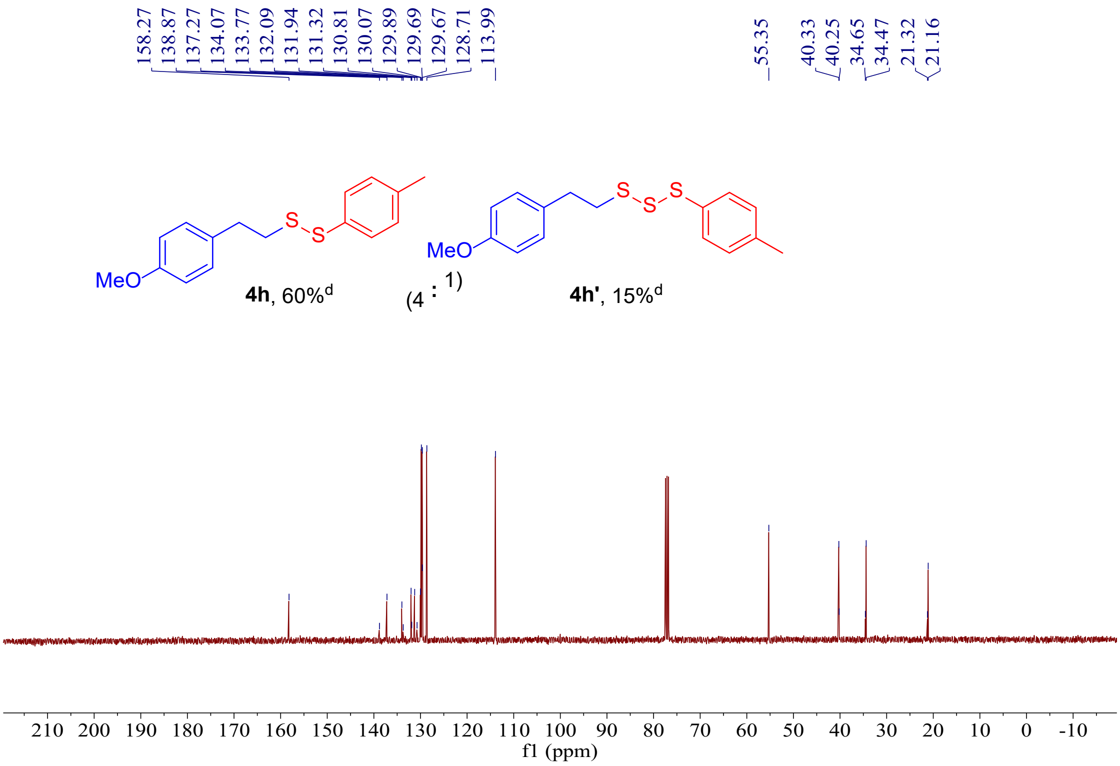

Supplementary Figure 55. ^{13}C NMR spectrum of **4h +4h’**.

^{1}H NMR Spectra of **7a** (400 MHz, room temperature, $CDCl_3$)

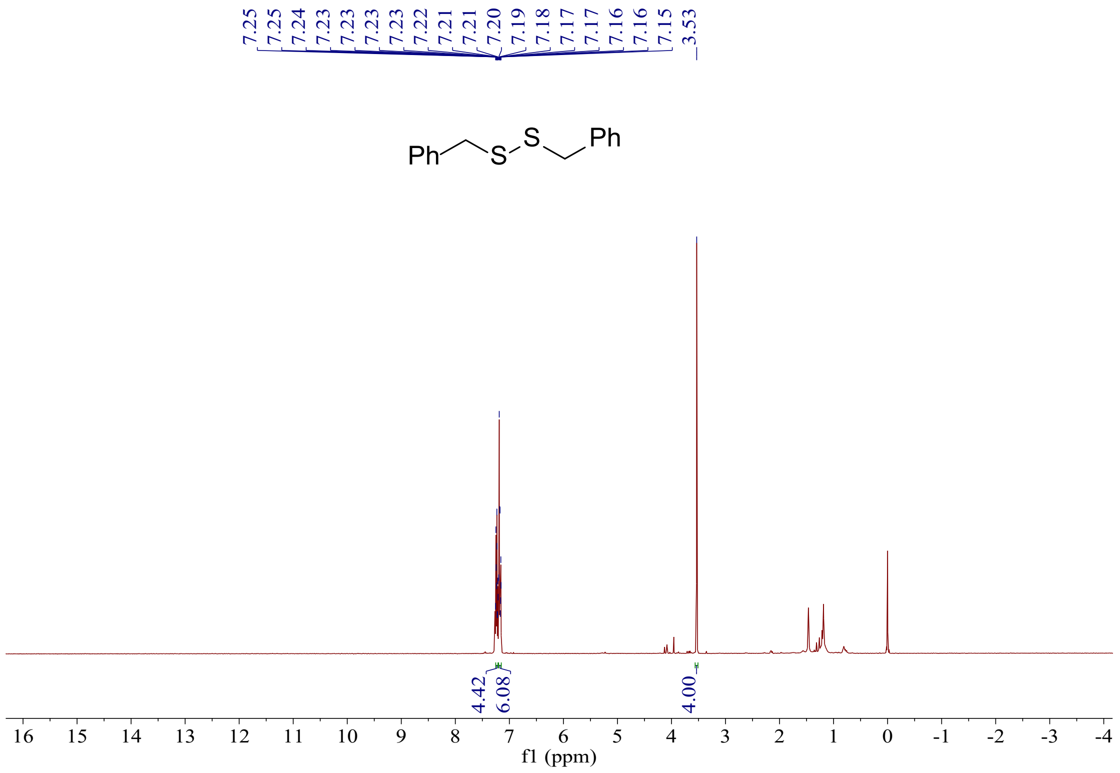

Supplementary Figure 56. ^{1}H NMR spectrum of **7a**.

^{1}H NMR Spectra of **8** (400 MHz, room temperature, $CDCl_3$)

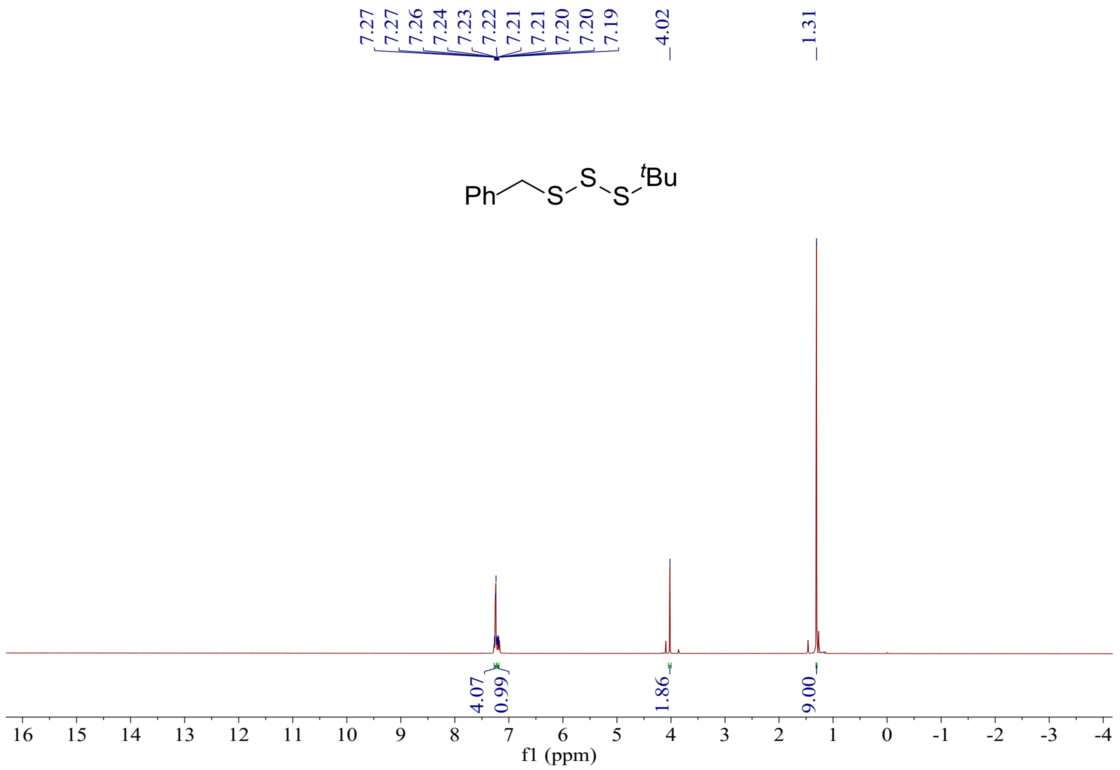

Supplementary Figure 57. ^{1}H NMR spectrum of **8**.

Supplementary References

(1) Chauvin, J. P. R.; Griesser, M.; Pratt, D. A. *Chem. Sci.* **2019***, 10,* 4999.

(2) Cerda, M. M.; Hammers, M. D.; Earp, M. S.; Zakharov, L. N.; Pluth, M. D. *Org. Lett.* **2017***, 19,* 2314−2317.

(3) Wang, W.-G.; Lin, Y.-Z.; Ma, Y.-D.; Tung, C. H.; Xu, Z.-H. *Org. Lett*. **2018**, *20*, 3829−3832.
